# Cryogenic Treatment of Martensitic Steels: Microstructural Fundamentals and Implications for Mechanical Properties and Wear and Corrosion Performance

**DOI:** 10.3390/ma17030548

**Published:** 2024-01-23

**Authors:** Peter Jurči, Ivo Dlouhý

**Affiliations:** 1 Department of Materials, Faculty of Material Sciences and Technology, Slovak University of Technology, Paulinska 16, 917 24 Trnava, Slovakia; p.jurci@seznam.cz; 2 Institute of Physics of Materials, Czech Academy of Sciences, Zizkova 22, 61662 Brno, Czech Republic; 3 Institute of Materials Science and Engineering, NETME Centre, Brno University of Technology, Technicka 2, 61669 Brno, Czech Republic

**Keywords:** steels, martensite, retained austenite, cryogenic treatment, carbides, microstructure, mechanical properties, wear performance, corrosion performance

## Abstract

Conventional heat treatment is not capable of converting a sufficient amount of retained austenite into martensite in high-carbon or high-carbon and high-alloyed iron alloys. Cryogenic treatment induces the following alterations in the microstructures: (i) a considerable reduction in the retained austenite amount, (ii) formation of refined martensite coupled with an increased number of lattice defects, such as dislocations and twins, (iii) changes in the precipitation kinetics of nano-sized transient carbides during tempering, and (iv) an increase in the number of small globular carbides. These microstructural alterations are reflected in mechanical property improvements and better dimensional stability. A common consequence of cryogenic treatment is a significant increase in the wear resistance of steels. The current review deals with all of the mentioned microstructural changes as well as the variations in strength, toughness, wear performance, and corrosion resistance for a variety of iron alloys, such as carburising steels, hot work tool steels, bearing and eutectoid steels, and high-carbon and high-alloyed ledeburitic cold work tool steels.

## 1. Introduction

Despite the rapid development of novel advanced material groups, traditional iron alloys still offer potential for new treatment processes to produce materials with enhanced performance. Thus, continued efforts have been directed towards developing newer materials to produce smarter products and improve the commonly used processing routes to improve the properties of existing materials [1]. In the last three decades, there has been a renewed focus on the use of cryogenic treatment (CT) to enhance the performance of engineering components [2,3].

Exploiting low temperatures to improve selected properties of tools and components is not a new approach [4,5]. For example, there are stories of Swiss watchmakers who stored their wear-resistant parts in caves high in the Alps to ‘stabilise’ the microstructure and to increase wear resistance [6]. Engine maker Pierce-Arrow from the United States, having a definite advantage in the technology due to their location in Buffalo, New York—where it is known to get quite cold in winter—used a cold treatment method for their engine blocks [7]. In the European machine industry, the history of CT can be traced as far back as the 1930s, when the German company Junkers used it for military aeroplane components. According to ex-Junkers engineer Luerker, it was a vital part of the engineering that went into their reliable Jumo 1000 HP V-12 aircraft engine [8]. After World War II, when he immigrated to the United States and ended up in California working for McCulloch Chain Saw Company in the mid-1950s, he suggested they use the process on chain saw blade links. They started cryogenically treating their chainsaw blades but kept it a secret so other manufacturers could not make better blades.

Cryogenic treatment is a process where the tools, components, or materials are immersed in a suitable cryogenic medium for an appropriate duration of time. The treatment is carried out at sub-zero temperatures, that is, from 0 to −269 °C. The components are brought down to sub-zero temperatures in cryogenic chambers of various designs. Cryogenic chambers can work on two basic principles, direct or indirect cooling. In direct cooling, the components or specimens are placed in the cryogenic chamber [9]. Then, the appropriate cooling medium is introduced into the chamber, where it is turned into cold gas to cool the materials down before they come into contact with liquefied gas. This method is the most efficient means of achieving very low processing temperatures. Indirect cooling can be realised in mechanical freezers. Liquid nitrogen or mechanical means can be used to cool the ‘secondary’ cooling medium in which the components are treated. This ‘secondary’ cooling medium can be ethanol or solid carbon dioxide (dry ice), or a mixture of the two. A general drawback of indirect cooling is the limited minimum processing temperature of around −100 °C.

Based on the lowest temperature of CT used, some authors have classified the treatments into three temperature ranges: ‘cold treatment’ (temperatures > −80 °C), ‘shallow cryogenic treatment’ (−80 to −160 °C), and ‘deep cryogenic treatment’ (<−160 °C) [10,11,12,13,14]. Different cryogenic media are used for the treatments. For temperatures > −80 °C, cold nitrogen gas has been used by some researchers [10,15], while others have employed mechanical freezers [16,17,18] filled with either dry ice [19] or a mixture of dry ice and ethanol [20]. For lower temperatures, down to −140 °C, cold nitrogen gas has been used [21,22,23,24]. In industrial applications as well as in laboratory experiments, the use of liquid nitrogen at its boiling temperature is widespread [16,19,25,26,27,28,29,30,31,32], while only a few authors have attempted to treat the specimens in liquid helium [33,34,35,36].

The application of specific temperatures for cryogenic treatment has also undergone notable development over the last decades [6]. In the 1950s and 1960s, it was commonly accepted that temperatures down to approximately −79 °C (−120 °F) were sufficient to transform a high portion of retained austenite (RA) into martensite, and that lower temperatures had no practical effect on steel microstructure. In addition, the acceptance of −79 °C (−120 °F) was a consequence of some trials which implemented direct soaking of the tools in containers of liquid nitrogen. The resulting thermal shock led to tool failure, and companies dropped this idea. A controlled treatment at the boiling point of liquid nitrogen (−196 °C) was suggested much later. This treatment further increased the performance of components, as demonstrated by examples of tools made of cryogenically treated AISI D2 or AISI D3 steel in real industrial performance (stamping dies, furniture manufacturing, powder compaction dies, and piercing or blanking punches) [1,6,37,38,39,40,41,42]. Consequently, CT has attracted the interest of scientists and has also found notable applications in different manufacturing industries such as automotive, aerospace, defence, mining equipment, and excavators.

The immersion time in the cryogenic medium is the second most important parameter (after temperature) affecting the microstructure and properties of metals. The first scientists who investigated the effect of immersion time on microstructures of carbon- and high-speed steels were Cohen [43] and Gordon and Cohen [44] in the 1940s. However, in further studies, immersion time was no longer considered an important factor in CT. Interest in CT was only rekindled in the 1990s. Since then, the effects of immersion time on the resulting microstructures and properties have been investigated for ball bearing steels [20,24,45], carburised steels [18,33,46,47], hot work tool steels [22,48,49,50,51,52], ledeburitic cold work tool steels [12,27,30,53,54,55,56,57,58,59,60], high-speed steels [61,62,63,64,65,66,67], and martensitic stainless steels [68,69,70,71,72].

The cooling rate is the third most important parameter affecting the microstructure and mechanical properties of materials, accounting for 9.34–14% of changes in them [73,74,75]. As mentioned above, industrial trials with direct immersion of treated parts in cryogenic media have failed. Direct immersion of laboratory specimens has also been used in pioneering investigations of cryogenic treatments [44,76]. Based on a number of studies on the effect of the cooling rate in the interval between room and cryogenic temperatures, slow cooling rates (in K s^−1^), namely, 0.5 [50,77,78], 0.75 [12,53], 1 [74,79,80], 2.5 [81], or 3 [3], have been recommended to prevent excessive deformation or cracking of treated components. The aforementioned phenomena can occur due to the significant differences in thermal expansion coefficients between austenite and martensite [82] and a volume change of up to 4% during the RA-to-martensite transformation [17,38]. Furthermore, some experimental trials indicate the most pronounced microstructural changes in steels cryo-treated at slow cooling rates (1–2 K s^−1^) [65]. Therefore, a general recommendation is that the cooling rate for most engineering iron alloys should be between 0.5 and 3 K s^−1^. This topic will not be discussed further in this review.

In some review articles over the last approx. two decades, authors have discussed the effects of cryogenic treatments on various metals and materials. They have focussed on specific topics and materials for which cryogenic treatment is used, for example, CT of cutting tools [5,83], the impact of CT on wear performance [4], and the use of CT in the automotive [84] and textile [85] industries. There are also general articles on the effects of CT on the microstructure and properties of metallic materials [1,2,42,86,87,88,89,90,91,92] and their weldments [93], as well as non-metallic [94,95] materials. Some of the general articles, however, were published many years ago (e.g., 2001 [1], 2008 [87]) or lack in comprehensiveness (short review papers [2,88,90]). There were also more comprehensive review articles published more recently but in lesser amounts. They cover a wide range of mechanical and other properties that are affected by cryogenic treatments, but the described microstructural alterations are limited to only the more complete austenite-to-martensite transformation and carbides precipitation [42,86,89,91,92]. A comprehensive review that covers not only the effect of cryogenic treatments but also the austenitising, quenching (prior to CT operations), and tempering of the microstructures (retained austenite transformation, martensitic microstructures, carbides precipitation, and formation of additional carbides), and their impact on the properties of iron alloys whose austenite is not completely transformed to martensite during conventional heat treatment (CHT) is still missing. 

The purpose of this review is to summarise the state of the art of CT of the main steel classes based on the effects of this treatment on microstructural alterations and associated changes in mechanical properties, wear performance, and corrosion resistance. Moreover, summarised findings in the field concerned can make a serious background for starting or continuing the research into phenomena in the field of cryogenic treatments of martensitic steels that were not clarified yet. Also, some of the principal outcomes can be utilised, for instance, in future research of CT of cast irons and non-ferrous metals and alloys.

## 2. Scope of the Review

**Figure 1:**
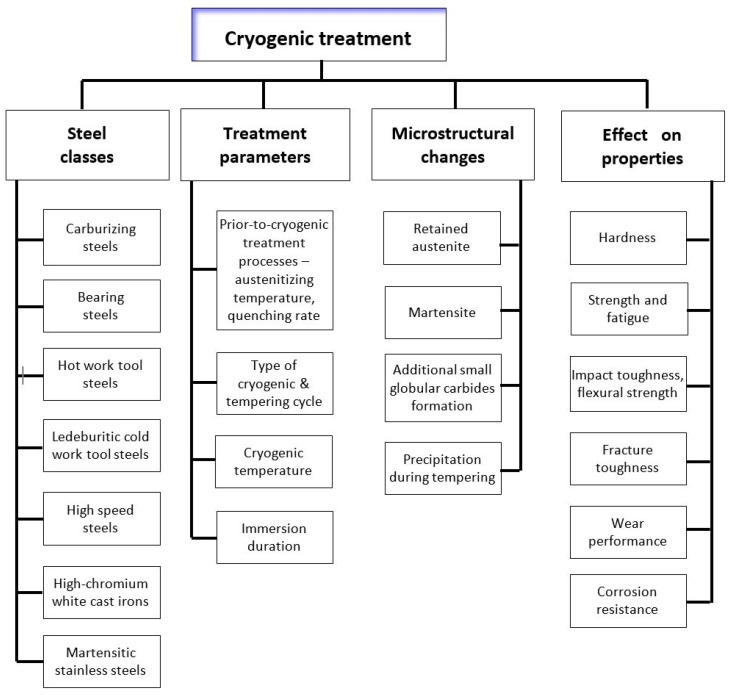
Steel classes, treatment issues, microstructural changes, and properties considered in this work.

Cryogenic treatment leads to various microstructural changes that differ from CHT in terms of the following features: (i) a reduction in the amount of retained austenite, (ii) a refinement of the martensite, (iii) changes in the precipitation kinetics of nano-sized transient carbides, and (iv) an increase in the number of additional small globular carbides. Figure 1 shows the classes of steels that are the subject of this study, the processing parameters discussed, and the effects of these processing parameters on the microstructural changes in and properties of these steels. This review includes the steel classes where the martensite is the main constituent of their as-quenched microstructures. These classes involve carburising steels (although the martensite is formed mainly in the carburised case), ball bearing steels, hot work tool steels, martensitic stainless steels, and ledeburitic tool steels (involves steels in which ledeburite appears in their as-solidified microstructures [96]). The latter concerns D-class tool steels, some newly developed powder metallurgy high-chromium high-vanadium steels, and high-speed steels. The processing parameters concern not only cryogenic treatment itself (temperature; immersion time) but also, where appropriate, the parameters prior to cryogenic treatment (austenitisation temperature; quenching). For the tempering treatments, the temperature and the sequence (before or after CT) are mainly discussed. The effects of the above processing parameters on retained austenite, martensite, quantitative parameters of carbides, and precipitation kinetics are described in detail in Section 3 of this review. The relationship between the above microstructural changes and mechanical properties such as hardness, strength, toughness, fracture toughness, and fatigue resistance (if applicable), as well as wear and corrosion resistance, are addressed in Section 4. 

**Figure 2:**
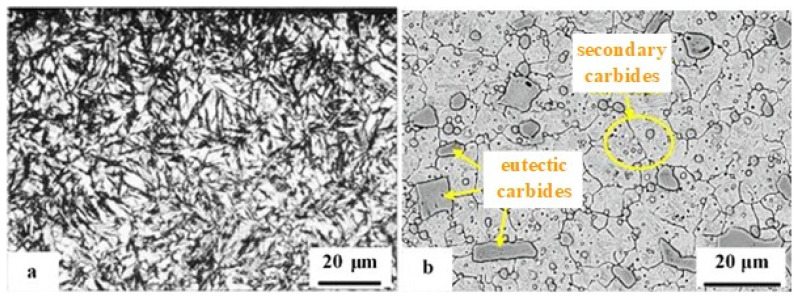
As-quenched microstructures of intrinsically homogeneous (**a**) and intrinsically non-homogeneous steels (**b**) involved in this study. A carburised case developed on 17CrNi6-6 steel is used as an example of intrinsically homogeneous steel [97], while as-quenched AISI D2 steel is used here as an example of an intrinsically non-homogeneous steel [98]. The microstructure of the carburised case contains the martensite and retained austenite as shown after 3% Nital etching, while undissolved carbides are shown in the as-quenched microstructure of AISI D2 steel besides the martensite and retained austenite after Villela–Bain etching.

Before starting cryogenic treatment, the steels must be quenched ‘conventionally’. The microstructures in the as-quenched state represent the initial microstructural states, which are further modified by CT. Depending on the nature of the microstructure in the as-quenched state, the steels discussed in this review can be divided into two groups: intrinsically homogeneous and intrinsically non-homogeneous. The first group includes mainly carburised steels (in the carburised surface region), hot work tool steels, and most martensitic stainless steels. These materials are fully austenitic after austenitising, and the austenite is transformed to a greater or lesser extent into martensite during quenching. Depending on the extent of the austenite-to-martensite transformation, the steels belonging to the first group contain martensite and certain amounts of retained austenite in their quenched structure (Figure 2a, [97]). The second group of steels investigated in this study comprises classes that, in addition to martensite and retained austenite in the quenched state, also contain undissolved carbides (eutectic; secondary) (Figure 2b, [98]). Ball bearing steels, ledeburitic cold work tool steels [99,100,101,102,103,104,105,106,107,108,109,110], or high-speed steels [111,112,113] are typical examples.

**Figure 3:**
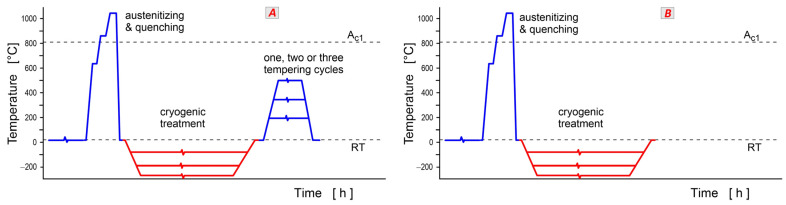

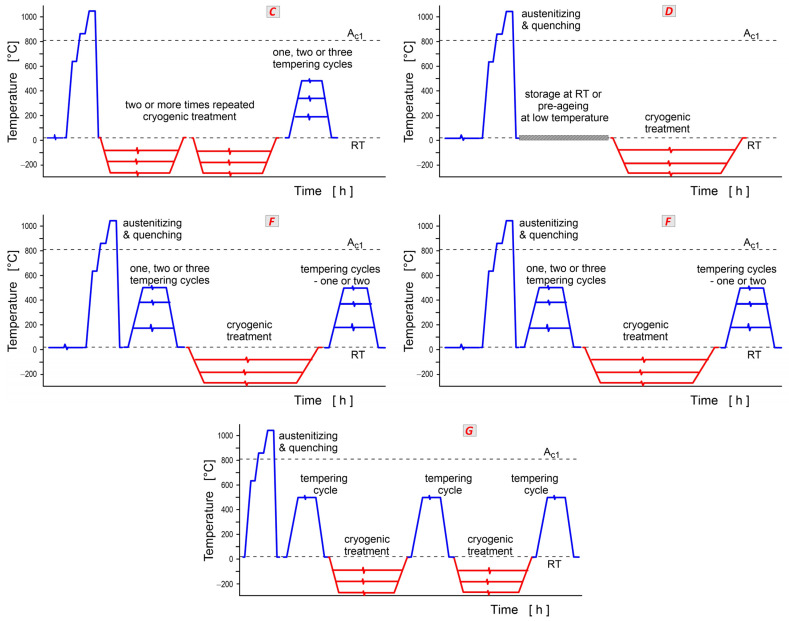
A schematic diagram of the heat treatment schedules used for the cryogenic treatment (CT) investigated in this work: (**A**) the ‘classical’ treatment sequence with post-tempering after CT; (**B**) the sequence without tempering after CT; (**C**) the sequence with repeated post-quenching and prior-to-tempering CT; (**D**) the sequence with interrupted cooling or pre-ageing prior to CT; (**E**) the sequence with CT after tempering; (**F**) the sequence with pre-tempering, CT, and post-tempering; (**G**) the sequence with multiple CT/tempering cycles. Legend: RT—Room temperature, Ac1—Temperature of the pearlite-austenite transformation.

There is a debate about heat treatment sequences and their effect on the result of final processing. A variety of sequences have been used. The ‘classical’ schedule with cryogenic treatment after quenching and prior to tempering, sequence *A* (Figure 3A), has been used the most, especially for martensitic stainless steels [16,17,69], high-speed steels [63,64,114,115], and ledeburitic cold work tool steels [12,15,116] but also for hot work tool steels [117,118]. To highlight the changes due to CT, some authors have examined specimens without tempering after CT, sequence *B* (Figure 3B) [17,30,114,116,119]. For experimental purposes, repeated CT after quenching and prior to tempering, sequence *C* (Figure 3C), has been used [17]. For the same reason, researchers have also utilised pre-ageing or interrupted cooling before immersion into the cryogenic medium, sequence *D* (Figure 3D) [98,120]. Sequence *E* (Figure 3E) with CT after tempering has also been used [62,64,114,121], mostly to compare the obtained results with the ‘classical’ schedule (sequence *A*). For the treatment of hot work tool steels, in particular, the cycles involving both pre-tempering and post-tempering, sequence *F* (Figure 3F), have been applied [22,51,122]. The number of pre- or post-tempering cycles may be one, two, or three. The last case, shown in Figure 3G, is represented by multiple CT/tempering cycles [62,114], sequence *G*. The temperatures and durations of individual tempering cycles may be quite different, from 100 up to 670 °C, and from 15 min to 4 h.

## 3. Microstructural Changes Due to Cryogenic Treatment

This section deals with microstructural changes due to the application of cryogenic treatments. In the first part, the general metallurgical background of these changes is described, namely, (i) variations in the retained austenite amount, (ii) alterations in the martensitic sub-structure, (iii) precipitation of nano-sized carbides during tempering, and (iv) additional small globular carbide (SGC) formation. Then, the changes are demonstrated for each steel class separately in further sub-sections. The reasons for this are that the extent of microstructural changes may be different for each of these classes, and also it may help the reader to understand better the interrelationships between microstructural changes and alterations in mechanical and other characteristics.

(i) Variations in the retained austenite amount and its other characteristics.

**Figure 4:**
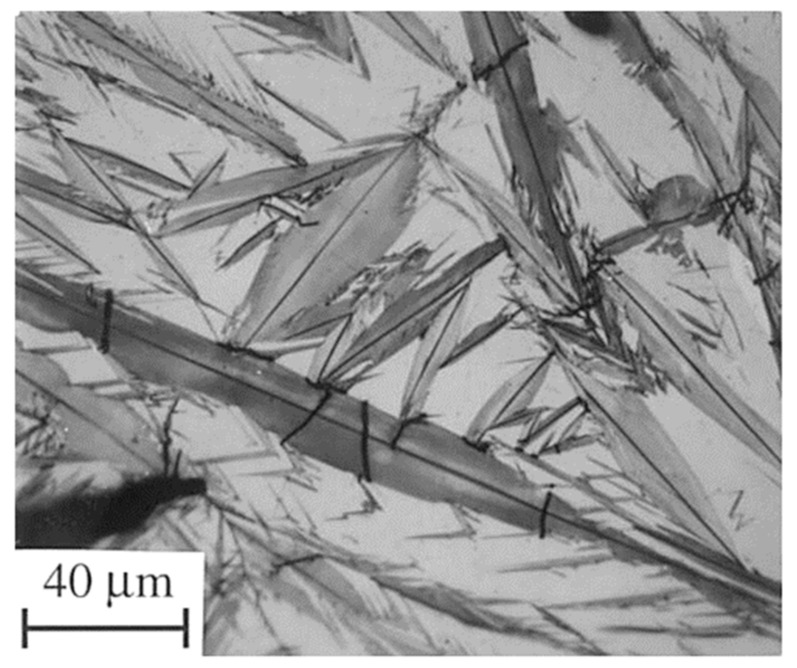
Light micrograph showing the microstructure of plate martensite with a high amount of retained austenite in an Fe-1.86 wt.% C steel, according to Krauss [124].

**Figure 5:**
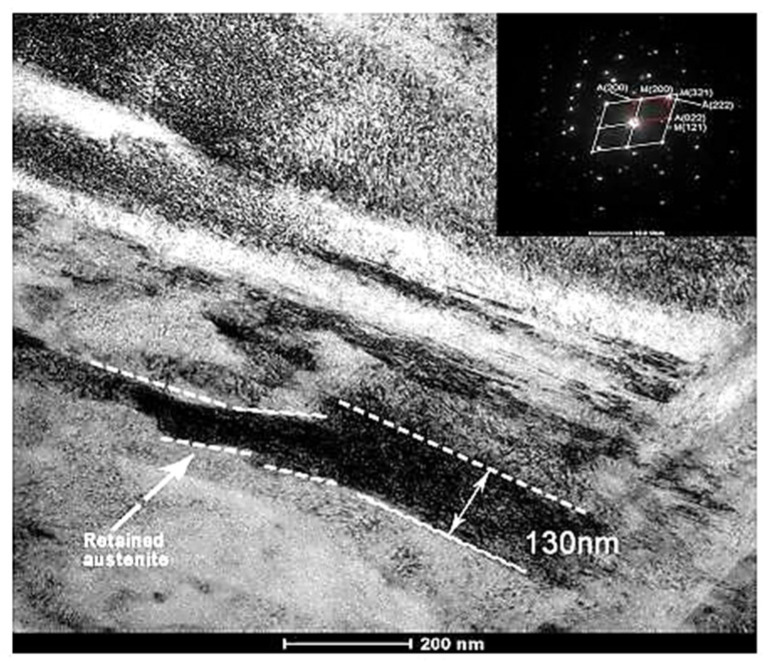
Transmission electron micrographs showing thin films of retained austenite between the martensite laths (interlath retained austenite) in conventionally heat-treated AISI H13 steel in an as-quenched state (austenitizing at 1040 °C for 40 min, followed by oil quenching) [125].

Hardening processes by quenching have been used to produce high-strength and wear-resistant tools and machine parts. In this process, martensite-containing microstructures are formed by diffusionless, shear-induced, martensitic transformation. The second phase present in hardened steels is retained austenite [123]. The retained austenite may be embedded between non-parallel martensitic plates in carburised regions in low-carbon steels, shown in Figure 4 [124]. In these cases, the RA is visible as white blocks in the room-temperature quenched microstructures. In high-carbon, high-alloy tool steels, the retained austenite usually appears as more or less thick films (several tens to >100 nm) between the martensitic laths, as an example in Figure 5 shows for conventionally heat-treated H13 steel [125]. In these steels, the amounts of retained austenite can be up to 20 vol.% [30].

The amount of retained austenite increases dramatically with increasing carbon content in room-temperature quenched carbon steel subjected to CHT. Increasing the austenitisation temperature has a similar effect on the RA amount [126,127].

There are several reasons to preserve/stabilise RA in medium- and high-carbon and/or high-alloy quenched steels. Both the martensite start temperature (M_s_) (Equation (1)) [128] and the martensite finish temperature (M_f_) decrease with increasing content of carbon and alloying elements dissolved in the parent austenite, and in many cases, the latter is in the sub-zero Celsius range [32,128,129,130].

(1) $$M_{s}\;\left[{}^{\circ}C\right]=539-423\times C-30.4\times Mn-12.1\times Cr-17.7\times Ni-7.5\times Mo+\left(10\times Co-7.5\times Si\right)$$

The austenite-to-martensite transformation has a positive volumetric effect; the extent of the volumetric change increases as the carbon content increases [131]. At the atomic level, this phenomenon is reflected by an increase in martensitic lattice tetragonality (c/a), which is proportional to the carbon content (Equation (2)) [132]:

c/a = 1 + 0.031 wt.% C. (2)

In most steels that are the subject of this review, a high amount of retained austenite is undesirable because this phase is soft and thus reduces the overall hardness of the steel [133]. Furthermore, RA is metastable at room temperature and therefore can be transformed—for example, under heavy load/stress during operation of the component [134,135]. Retained austenite transformation is associated with dimensional changes that can adversely affect the durability of tools and components [32,136,137]. Another effect of RA transformation is that the product of this process is martensite, which reduces the plasticity of steel [138]. Therefore, to obtain an appropriate functional performance of components or tools, it is highly desirable to remove RA from most steels before putting them into service.

It should be noted that the presence of a certain RA amount cannot be inevitably ‘undesirable’ in ball bearing steels. Mechanically induced transformation of austenite during rolling contact fatigue is considered beneficial in regions where the stresses or strains are localised [136,139,140]. Such a transformation can also lead to the development of favourable residual stresses. There is also evidence of a positive effect of RA on flexural fatigue [141] or fatigue crack propagation [142,143,144]. On the other hand, these benefits have to be weighed against other consequences such as lower hardness, lower elastic limit [145], or poorer dimensional stability [137,146]. Therefore, it is highly desirable to control the amount of RA by using a suitable heat treatment process. In many cases, CHT is not sufficient to convert much of the austenite to martensite or to bring its amount to the desired level. One way in which retained austenite could be removed/controlled is by tempering at high temperatures. However, some types of steel (e.g., carburised steels; ball bearing steels) cannot be tempered above ~200 °C because they would suffer a significant loss of hardness [147]. An alternative is to include CT in the processing procedure.

The RA-to-martensite transformation during cryogenic treatment has been a generally accepted and scientifically proven phenomenon since the 1920s [43,148,149,150]. Also, it is known that the use of −196 °C (or a lower temperature) is much more effective in RA reduction than the use of CT temperatures in the range from −70 to −120 °C [12,16,53,54,63,69,70,71,81,151,152,153,154,155,156,157,158,159,160,161,162,163,164,165,166,167,168,169,170,171,172], in most cases, for all steel classes presented in the current work. 

The RA-to-martensite transformation is isothermal and time-dependent during CT, as has been suggested [32] and proven experimentally for many materials such as ball bearing steels [45,173] and different ledeburitic cold work tool steels [57,98,116,119,174]. The temperature range in which the isothermal RA-to-martensite transformation is most active lies between −140 and −196 °C [30,57,98,119,174]. Lower CT temperatures are less effective because the isothermal transformation of retained austenite is always accompanied by plastic deformation of freshly formed martensite, and the plastic deformation rate decreases with decreasing temperature. Conversely, the upper temperature limit of the isothermal RA-to-martensite transformation can be determined as the temperature at which the interstitial atoms become mobile. This temperature can be quite different for each alloy and can be −33 °C for AISI D2 steel [98] or −65 °C for carbon steels [175].

The retained austenite is already in a high state of compression after conventional quenching. This is because the retained austenite domains are ‘encapsulated’ between martensitic domains and are compressively stressed as a result of the positive volume change in the RA-to-martensite transformation [176,177]. Lu et al. [20] established that quenching 100Cr6 steel from the austenitisation temperatures of 860, 920, and 1150 °C produced compressive macro-stresses (phase-dependent) in RA of 60, 80, and 230 MPa, respectively. The application of CT at −65 °C doubled these stresses at the given austenitisation temperatures. In high-alloyed tool steels, the stresses may be even higher (exceeding a value of 1000 MPa) [15,21].

The higher compressive macro-stresses obtained by CT act against further martensitic transformation; this treatment is an effective method to stabilise retained austenite. During tempering, these stresses were partially relieved, a phenomenon related to the RA transformation. However, this transformation requires a volume increase, which is only possible if the tetragonality of the surrounding martensite decreases. It is known that a reduction in the martensite tetragonality is accompanied by carbide precipitation [178]. Therefore, the precipitation of transient carbides seems to be a prerequisite for stress relief in RA and its decomposition. The stress relief is more pronounced during high-temperature tempering and thus destabilises the RA. This was demonstrated in an example of Vanadis 6 steel [21,116,179], where it was proven that cryogenic treatment followed by tempering at >450 °C accelerated RA decomposition compared to the post-CHT state. 

Carbon partitioning from martensite to retained austenite also occurs during CT. Qiao et al. [31] observed that the quenched samples of 100Cr6 steel contained 1.01 wt.% C in the RA. When the duration of CT (at −196 °C) reached 240 h, there was 1.26 wt.% C in the RA. One might expect that the enrichment of austenite by carbon would lead to its greater stability and thus contribute to stabilising this phase together with the high compressive stresses. However, as mentioned in the text, the situation is more complex, and the (probable) enhanced precipitation rate of nano-sized carbides counterbalances different stabilising effects on the retained austenite.

(ii) Alterations in the martensitic sub-structure.

The formation of refined martensite is one of the key features generated by the cryogenic treatment of iron alloys. The refinement of martensitic domains has been reported independently by many investigators for carburised steels [180,181], ball bearing and near-eutectoid steels [180,182,183], chromium–vanadium (Cr-V) ledeburitic cold work tool steels [30,184], different high-speed steels [10,61,164,168,185,186], and martensitic stainless steels [17,69,177,187]. Moreover, Xu et al. [186] established that refinement concerns not only the size of martensitic laths/needles but also the width of the internal twins inside them. 

There are two phenomena that can plausibly explain the martensite refinement caused by CT. The first phenomenon is based on the fact that the matrix is fully austenitic before reaching the M_s_ temperature; therefore, the martensitic domains grow freely at the beginning of the transformation. After room-temperature quenching, RA formations are encapsulated within the existing martensite [177]. During CT, the martensitic transformation progresses within these austenitic formations, but the growth of martensitic domains is limited by their size.

The second phenomenon is based on the fact that the martensitic transformation is athermal in conventional quenching [188], while the process that occurs at a very low temperature may manifest symptoms of thermal activation [187,189,190]. Virgin (or freshly formed, soft, and ductile) rather than aged martensite is formed at cryo-temperatures [191,192]. Virgin martensite can deform plastically [175,193], a phenomenon that is reflected by a considerably enhanced density of crystal defects such as dislocations and twins within martensitic domains [116,173,178,184]. The plastic deformation of virgin martensite originates from several sources: (a) There is a considerable contraction of both martensite and austenite while cooling to the cryo-temperature. The extent of this contraction is distinct for each phase because they manifest clear differences in thermal expansion coefficients (23.0 × 10^−6^ K^−1^ for austenite vs. 11.5 × 10^−6^ K^−1^ for martensite [82]). (b) There is volume expansion resulting from the RA-to-martensite transformation; the extent of expansion mainly depends on the carbon content in the parent austenite [17]. (c) High compressive stresses are generated in retained austenite [21,176,177], while martensite is tension strained. In addition, plastic deformation is associated with dislocation movement (albeit slow at low temperatures) and with the capture of carbon atoms by these dislocations [30,79,119,184,194]. In other words, the isothermal part of the martensitic transformation may be accompanied by mass transfer, which is responsible for the growth control of martensitic domains and thus for significant refinement of martensite formed at cryo-temperatures.

Cryogenic treatments modify the tetragonality of the martensitic lattice, but there is no consensus on the extent of this change in the scientific community. For instance, Villa et al. [152,176,177] reported almost no change in tetragonality for cryogenically treated 1% C–1.5% Cr steel. Other investigators [21,125,184,194] have proven experimentally the very low tetragonality of the martensitic lattice for cryogenically treated Vanadis 6, X220CrVMo13-4, DC 53, and AISI H13 steels, while Das et al. [12,53] assumed and Pellizzari et al. [195] experimentally proved increased lattice tetragonality in AISI D2 steel (note that these steels will be discussed in Section 3.4). 

For the steels with very low martensitic lattice tetragonality after cryogenic treatment, partial recovery of the tetragonality, which occurs during low-temperature tempering [21,119], is explained by the effect of precipitation of nano-sized coherent carbides (as a final stage of martensite pre-ageing [191,192]). This is supposed to generate distortion at the interface between the carbon-depleted matrix and the carbon-rich particles, contributing to an increase in tetragonality. It has also been demonstrated that the tetragonality of martensite is due, among other things, to a coherent bond between the secondary phases (inclusions; precipitates) and the matrix [196].

(iii) Influence of cryogenic treatment on the precipitation of nano-sized carbides during tempering.

Altered precipitation kinetics of nano-sized carbides during tempering is the third typical consequence of the cryogenic treatment of steels that contain martensite and retained austenite in their as-quenched microstructures. The precipitation kinetics were found to be enhanced in most of the experimental works dealing with cryogenic treatments of carburised steels [180], ball bearing steels [152,178,197,198], hot work tool steels [51,122,199,200], ledeburitic cold work tool steels [21,116,120], high-speed steels [61,64], or martensitic stainless steels [16,69].

A plausible explanation for the enhanced precipitation rate of nano-sized transient carbides could be based on the fact that during plastic deformation of freshly formed martensite at cryo-temperatures, carbon atoms are trapped by gliding dislocations. The trapped C atoms form clusters at the dislocations, which are preferential sites for further carbide precipitation. Evaluation of temperature-dependent internal friction spectra of cryogenically treated tool steels has confirmed that more carbon atoms clustered at dislocations before tempering than was achieved by conventional room-temperature quenching [79,116,194,201,202,203]. Transmission electron microscopy confirmed accelerated precipitation of transient ε-carbides or cementite in the same studies. 

Alternatively, some early scientific reports claimed ‘almost no effect’ of cryogenic treatments on the decomposition of iron–carbon martensite in high-carbon steels [82,192,204] or that this decomposition is delayed [191]. Suppressed and delayed precipitation of transient ε- or η-carbides was also reported by Gavriljuk et al. [119,205,206] for AISI D2 steel after CT at either −150 or −196 °C. These investigators considered a possible higher binding enthalpy between carbon and dislocations compared to the formation enthalpy of transient carbides as the main sources of suppressed and delayed precipitation of carbides at low temperatures. Therefore, they assumed that the carbon clusters formed at dislocations during the cryogenic period could not act as nuclei for the precipitation of transient carbides. 

On the contrary, there is a consensus on significantly suppressed precipitation of stable carbides in cryogenically treated high-chromium and high chromium–vanadium steels. This suppression is one of the possible sources of the disappearance of the secondary hardness peak of these materials when tempered around 500 °C [21,116,119,179,206].

(iv) Additional small globular carbide (SGC) formation.

Thorough investigations of various steel grades after different cryogenic treatments over the last three decades have brought in the first sight “surprising” result. Some steels contained an enhanced number and population density of carbide particles, while other materials did not contain any such particles. These particles were mostly of a regular shape, with sizes of 0.5 μm or lower, and they were more or less uniformly distributed throughout the matrix. Also, it is interesting that even though many research groups (e.g., [12,21,61,65,67,159,207,208,209,210]) have provided clear and statistically relevant evidence for the presence of these carbides in cryogenically treated steels, some other investigators did not report these carbides even in the same or similar steel grades [98,119,120,195,211].

First, the terminology of the carbides described in this section needs to be clarified. Many authors have detected more carbides in tempered states, which can be traced back to cryogenic treatment. Since the first discovery by Collins and colleagues [81,156], these particles have been called ‘precipitates’ or ‘precipitated secondary carbides’ [12,54,159,160,208]. It has been suggested that these carbides are formed during tempering of more or less unspecified ‘pre-conditioned’ martensite formed at cryogenic temperatures [12,54,56,78,81,156,160,161,212]. However, in these works, the tempering regimes were kept constant; therefore, it was not possible to observe that (a) the additional small globular carbides appear in the microstructures of steels already before tempering, shortly after CT [21,210], and that (b) the number and population density of these particles decreases with the tempering temperature. Furthermore, secondary cementite (or secondary carbides in general) is defined as cementite (or carbides) formed in hypereutectoid steels when cooled below the characteristic A_m_ temperature due to decreasing carbon solubility in austenite [213], rather than cementite formed by the thermally activated decomposition of supersaturated solid solutions. Moreover, ‘precipitation’ is defined as a new phase formation from a supersaturated solid solution by a thermally activated process [214], during tempering, for instance. Therefore, the term additional small globular carbides (SGCs) is used in this review. 

The initial attempts to explain the formation of additional carbides led to the hypothesis that these particles are formed during tempering. Carbon atoms were expected to segregate to nearby dislocations during CT, where they form clusters that act as nuclei or grow into nuclei during tempering up to 210 °C [12,27,53,54,55,56,78,158,159,160]. The number of these clusters increases when the CT temperature decreases and the treatment time is prolonged. The major drawback of this hypothesis is that carbon atoms are essentially immobile at temperatures below −100 °C [119]. Therefore, they are unlikely to diffuse (or segregate) into nearby crystal defects. Furthermore, it is highly unlikely that particles typically 100–500 nm in size could be formed by a thermally activated process (e.g., by precipitation from martensite) during low-temperature tempering. For comparison, transient precipitates of η- or ε-carbides or cementite identified by various authors [21,119,120,215] in different low-temperature tempered steels are very thin needle-like particles with a length of a few tens of nanometres.

An alternative concept for the formation of additional SGCs in cryogenically treated ledeburitic steels has been recently proposed [216]. This concept is based on the findings that the SGCs appear in the microstructures of these steels already prior to tempering and manifest clear indications of plastic deformation (note that this is only possible when the deformation rate is very low [217,218,219,220,221]), and their chemistry does not differ from that of the matrix [21]. Therefore, the formation of SGCs during cryogenic treatment could be considered a by-product of the more complete martensitic transformation, and they are formed at cryo-temperatures.

### 3.1. Carburised Steels

Typically, 30 vol.% or more of austenite can be retained in the martensitic microstructure of high-carbon steels or carburised steels with a carbon content of 0.8 wt.% [222]. Steels with higher additions of nickel are especially prone to stabilisation of retained austenite in the areas near the carbon-enriched surface [223].

Moreover, attempts were made to understand the enhancement of retained austenite amounts due to the presence of secondary cementite in carburised cases [224]. However, the results did not allow us to make a conclusive statement in this respect yet. 

Various research groups have also reported extensive RA transformation during cryogenic treatment for carburised steels (see the overview of investigated steels in Table 1). Furthermore, these results are mostly consistent with the abovementioned general tendency; the use of −196 °C (or a lower temperature, combined with sequence *A*) is much more effective than the use of CT temperatures in the range from −70 to −120 °C [33,180,225,226,227,228]. For nickel-free grades such as 1.7131 [46] or 20MnCr5 [33], the RA can be reduced to practically an immeasurable amount. A substantial retained austenite reduction is also possible by applying CT at either −196 or −269 °C to carbon-supersaturated carburised cases. Cryogenic treatment is also an effective way to reduce the retained austenite in nickel-containing carburising steels. Even though they contain more than 20 vol.% of RA after conventional quenching, the retained austenite can be reduced to one-half [47,225,227,229] or one-fourth [180] by applying sequence *A*. A typical example is the work by Yan et al. [229], who reported retained austenite reduction from 18.15% (CHT) to 12.92, 10.73, and 9.45% for CT at −80, −150, and −196 °C. On the other hand, the effect of cryogenic treatment on retained austenite reduction is suppressed when steels are pre-tempered prior to CT (sequences *E* or *F*), which is due to the stabilisation of retained austenite [230,231]. 

**Figure 6:**
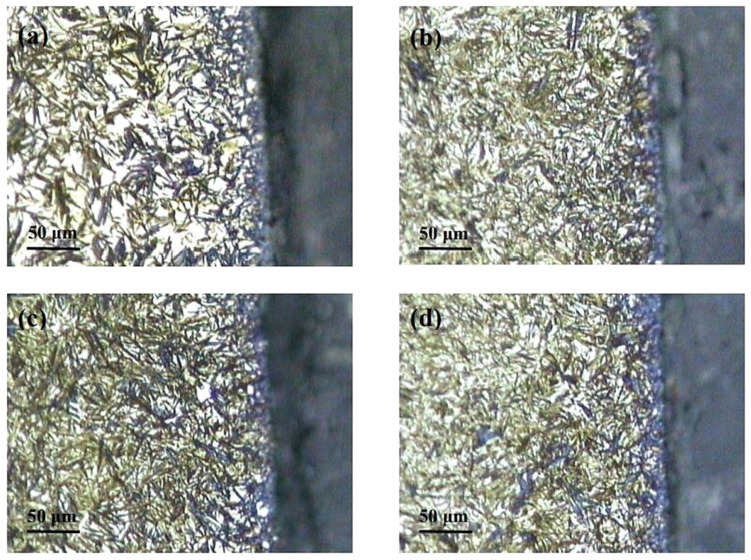
Cross-sectional light micrographs showing the microstructures of specimens made from SNCM 415 steel subjected to (**a**) CHT—gas carburising at 925 °C for 1 h followed by diffusing at 870 °C for 30 min, subsequent oil quenching, and 200 °C tempering for 1 h; (**b**) CT (inserted between quenching and tempering, sequence *A*) at −85 °C for 1 h, (**c**) CT at −85 °C for 12 h, and (**d**) CT at −85 °C for 24 h. Adapted from [225].

**Figure 7:**
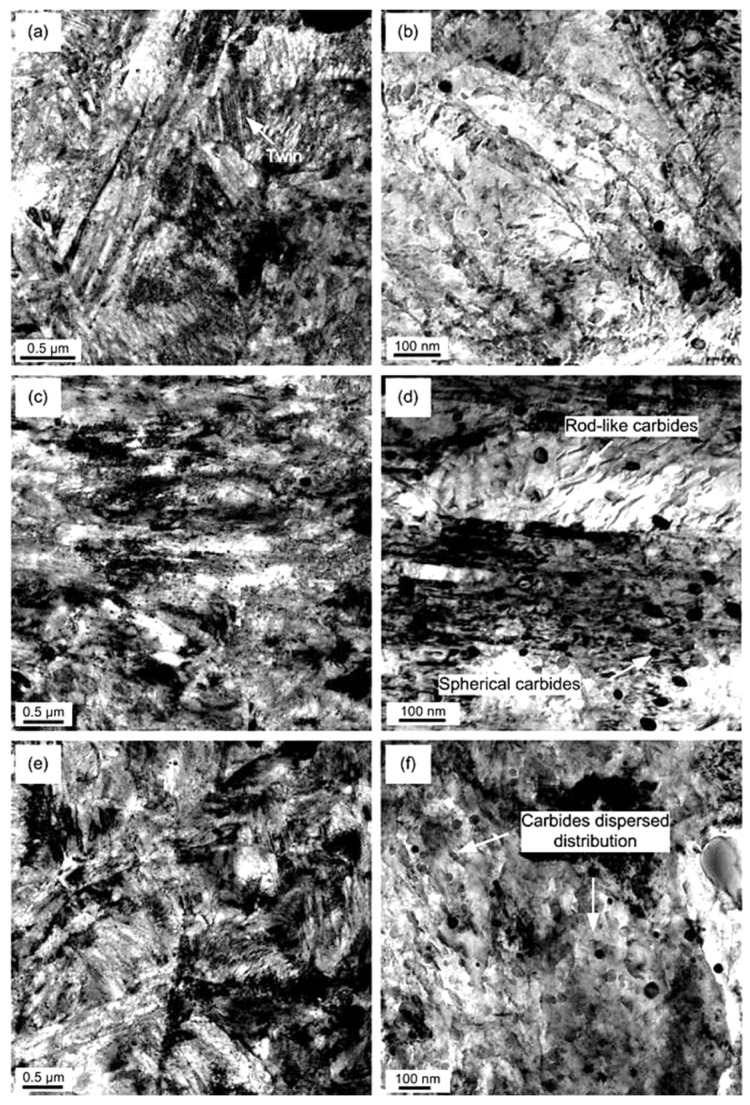
TEM micrographs showing the microstructures of samples made from 20CrNi2MoV after subjecting them to different heat treatment strategies: (**a**,**b**) CHT—carburising at 935 °C for 6 h followed by diffusion at 880 °C for 4 h, oil quenching, and 180 °C tempering for 2 h, (**c**,**d**) CT at −80 °C for 4 h, and (**e**,**f**) CT at −196 °C for 4 h. Cryogenic treatment was inserted between quenching and tempering (sequence *A*). Adapted from [180].

Figure 6 shows microstructural development in the carburised case of nickel-containing SNCM 415 steel that was subjected to cryogenic treatments at −85 °C for different durations. Visual inspection of the micrographs shows a clear retained austenite reduction due to cryogenic treatments. Moreover, the martensitic microstructure of cryogenically treated specimens manifests clear refinement as compared with the state after CHT. This is in line with other observations by Li et al. [180] and Ghosch and Dhokey [181], who observed refined martensite in 20CrNi2MoV and SAE 8620 steels. Changes in the precipitation kinetics of nano-sized carbides have been studied by Li et al. [180]. They reported a significantly enhanced number and population density of nano-sized precipitates, which was attributed to subjecting the material to CT, shown Figure 7. 

### 3.2. Ball Bearing Steels

**Figure 8:**
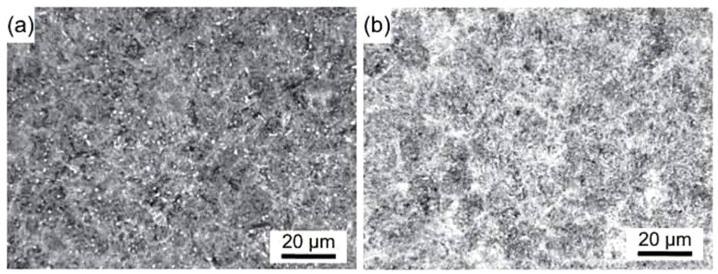
As-quenched microstructures of AISI 52100-type steels: light micrograph showing the martensite and retained austenite in the steel after oil quenching from (**a**) 820 °C and (**b**) 860 °C (etched with 2% Nital reagent). Adapted from [242].

**Figure 9:**
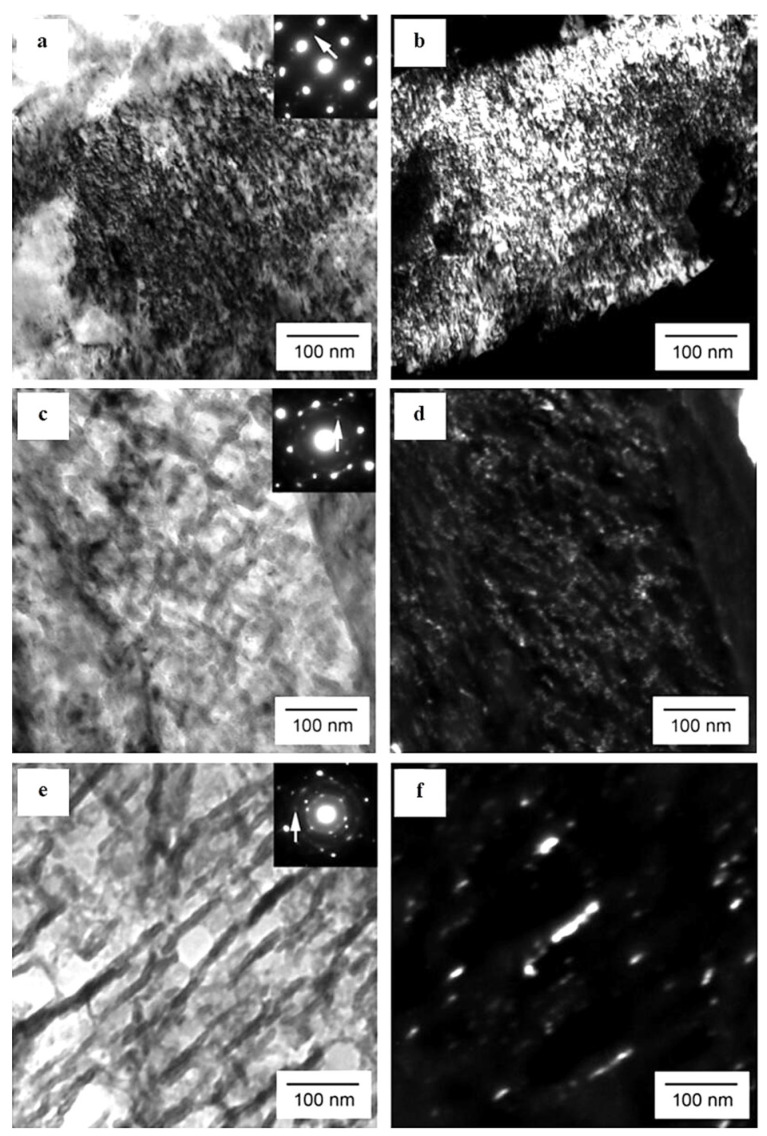
TEM micrographs showing a precipitation sequence of carbides in conventionally heat-treated (austenitizing at 860 °C for 30 min, followed by oil quenching) 100Cr6 with tempering at 160 °C for different durations. Legend: ε-carbide after tempering for 0.25 h where (**a**) is the bright-field image and (**b**) is the dark-field image (taken from the [1102]_ε_ reflection indicated by the arrow), η-carbide after tempering for 2 h where (**c**) is the bright-field image and (**d**) is the dark-field image (taken from the [110]_η_ reflection indicated by the arrow), and cementite after tempering for 4 h where (**e**) is the bright-field image and (**f**) is the dark-field image (taken from the [060]_C_ reflection indicated by the arrow). Adapted from [243].

Ball bearing steels (the overview is in Table 2) are steels with carbon contents in the range of 0.8–1.1 wt.% and a total substitutional solute content of less than 3 wt.% [240]. They are mostly made from martensitic domains by quenching in oil or salt from a temperature where the material is mostly austenitic. Then, they are subjected to low-temperature tempering in order to balance strength, hardness, and toughness. Among many steel compositions, the 1C–1.5Cr-type alloys (AISI 52100, 100Cr6, and En31) have become extremely popular. Quenching these steels from the standard austenitisation temperatures leads to a microstructure containing martensite, 7–16 vol.% of retained austenite [20,24,45,207,241,242], and 3–4 vol.% of cementite particles which failed to dissolve during austenitisation, shown in Figure 8. These particles are normally uniformly distributed, have a size of around 0.5 μm, and help to improve the wear resistance of steels. The steels are then tempered at temperatures up to approximately 200 °C, a process which may lead to the precipitation of a variety of transitions or more stable iron carbides from the supersaturated martensite. These carbides include ε-, η-carbide, and cementite, shown in Figure 9 [243].

**Table 2:** Overview of ball bearing steels and eutectoid steels and their cryogenic treatment covered by this review showing key investigations carried out: M—microstructure (p in the column—includes phase transformations); A—retained austenite; C—carbide precipitation; H—hardness (t in the column includes tensile properties); R—residual stresses; W—wear resistance and tribology; O—corrosion resistance. The designation “x” means that the particular microstructural feature/mechanical property was investigated in the referenced paper.

Steel Grade/Designation	Main Element Content (wt.%)	Conditions of Cryogenic Treatment	M	A	C	H	R	W	O	Reference
100Cr6	1.05 C and 1.46 Cr	−65 °C/0.5 h	x			t				[20]
AISI 52100	0.90 C and 1.49 Cr	−185 °C/24 h	x			x		x	x	[24]
AISI 52100	0.99 C and 1.42 Cr	−196 °C/0.5–240 h	x	x						[31]
AISI A2	1 C, 5 Cr, 1 Mn, 1 Mo, and 0.15–0.50 V	−196 °C/24 h or −269 °C/0.5 h	x					x		[34]
AISI 52100	1.03 C and 1.53 Cr	−145 °C/12–60 h	x			x				[45]
80CrMo12 5	0.8 C, 3.06 Cr, and 0.5 Mo	−80 °C/24 h or −196 °C/1 min–168 h	x			x		x		[80]
N/A	0.86 C and 0.99 Mn	−190 °C/12 or 36 h	x			x		x		[151]
AISI 52100	0.96 C and 1.6 Cr	−150 °C/1 min or 72 h; −110 °C/24 h	x		x					[152]
100Cr6	1 C and 1.4 Cr	−196 °C/24 h	x			x		x		[173]
AISI 52100	0.96 C and 1.6 Cr	−140 or −196 °C/1 min–7 h	p							[176]
AISI 52100	0.96 C and 1.6 Cr	−140 or −196 °C/1 min–7 h	x				x			[177]
100Cr6	1 C and 1.5 Cr	−196 °C/5 min or 24 h	p							[178]
N/A	0.85 C, 0.4 Mn, and 0.15 C	−40 °C/24 h	x					x		[182]
ABNT 52100	0.93–1.05 C, and 1.35–1.60	−196 °C/4 or 24 h		x						[183]
exp. steel	0.97, 1.20, or 1.59 C	−196 °C/different durations	p							[189]
AISI 52100	0.93–1.05 C or 1.35–1.60	−196 °C/6 h, one, two, or three times	x			x		x		[207]
AISI 52100	0.93–1.05 C or 1.35–1.60	−196 °C/24 h	x	x		x			x	[210]
100Cr6	0.91 C, 0.47 Cr, 0.29 Cu, and 0.33 Mn	−120 °C/2 h	x			x		x		[234]
100Cr6	0.97 C and 1.43 Cr	−185 °C/36 h	x			x		x		[244]
100Cr6	0.97 C and 1.43 Cr	−185 °C/36 h	x		x	x		x		[245]
En 31	0.99 C, 1.45 Cr, 0.25 Ni, and 0.3 Cu	−196 °C/24 h	x			x		x		[246]
AISI 52100	0.93 C and 1.4 Cr	−196 °C/24 h	x						x	[247]
100Cr6	0.97 C and 0.43 Cr	−100 °C/3.5 h	x			x		x		[248]

**Figure 10:**
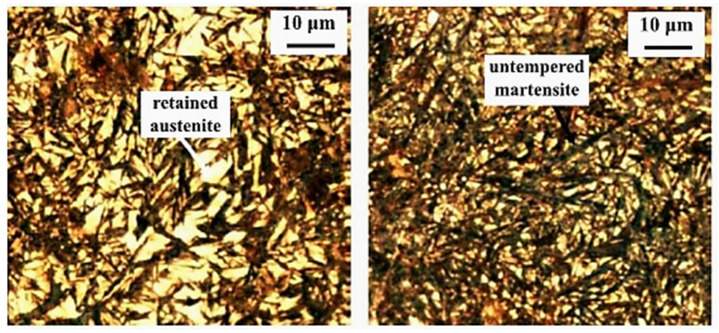
Light micrographs showing the microstructures of En 31 ball bearing steel after austenitizing at 820 °C for 1 h followed by room-temperature (conventional) quenching (**left**) and after subsequent cryogenic treatment at −196 °C for 24 h (**right**). Etched by 2% Nital reagent. Adapted from [249].

Cryogenic treatments foster the RA-to-martensite transformation in ball bearing steels. An overview of ball bearing steels and their cryogenic treatments can be found in Table 2. According to Lu et al. [20], treatment at −65 °C for 30 min reduced the retained austenite amount to one-half. However, treatments at lower temperatures were found to be more effective in retained austenite reduction. For instance, Gunes et al. [45] reported reducing the RA amount from 7.1 to 2.6 vol.% as a result of −145 °C treatment for 60 h (sequence *F*). The use of liquid nitrogen at its boiling temperature (or close to this point, sequence *A*) reduced the RA amount to values that are close to the detection limit of XRD [24,31,173]. Zhou et al. [207] carried out a cyclic cryogenic treatment (sequence *C*) to treat a 100Cr6 and found that most of the RA was eliminated after the first CT cycle at −196 °C, and subsequent cycles had little effect. It was also found that the RA-to-martensite transformation manifests clear evidence of time-dependency when the steels are treated at either −145 or −196 °C [31,45]. Figure 10 provides a clear insight into retained austenite reduction in EN 31 bearing steel due to cryogenic treatment at −196 °C for 24 h.

For ball bearing and near-eutectoid steels, the refinement of martensitic domains has been reported independently by Li et al. [180], Putu Widiantara et al. [182], and Moreira et al. [183]. Moreover, Xu et al. [186] established that refinement concerns not only the size of martensitic laths/needles but also the width of the internal twins inside them.

**Figure 11:**
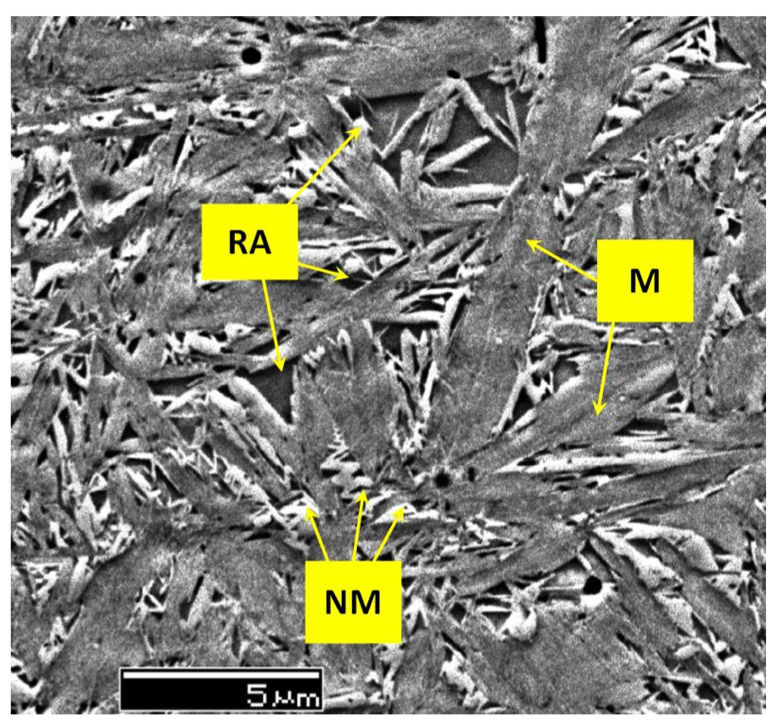
Backscattered electron micrograph of AISI 52100 steel after quenching, cryogenic treatment at −196 °C, and instant re-heating to room temperature. There was electrolytic etching in Struers A-2 solvent at 30 V for 40 s on a surface area of 5 mm^2^. Retained austenite regions appear darker and embedded between the martensitic plates. New martensite (NM) is fine and appears lighter than martensite (M) developed prior to cryogenic treatment. RA is the retained austenite. Adapted from [177].

A common feature of the aforementioned refinements is that they occur over a wide temperature range (from −72 to −196 °C) and at different CT durations. The refinement of martensite affects the domains that had grown during CT. In contrast, the martensite formed during conventional quenching (at room temperature) remains unaffected by CT (the martensite refinement contributes to strengthening that can be expressed by the Hall–Petch relation [250]). Figure 11 shows the microstructure of AISI 52100 steel obtained by CT at −196 °C. The newly formed martensite (during cryogenic treatment) shows multiple refinements in both the length and width of its domains compared with athermally formed martensite.

Early results on the decomposition of iron–carbon martensite of high-carbon steel treated by either CHT or CT (usually in liquid nitrogen) were contradictory. Some authors claimed that CT either had ‘almost no effect’ on the decomposition kinetics [82,192,204] or delayed the initial stages of decomposition [191]. In contrast, others noted a rather accelerated decomposition rate of the martensite at low temperatures due to the lower activation energy required for this process [197,198]. The results obtained in later works have supported the claim about the increased precipitation rate of transient carbides in cryogenically treated high-carbon steels [152,178].

**Figure 12:**
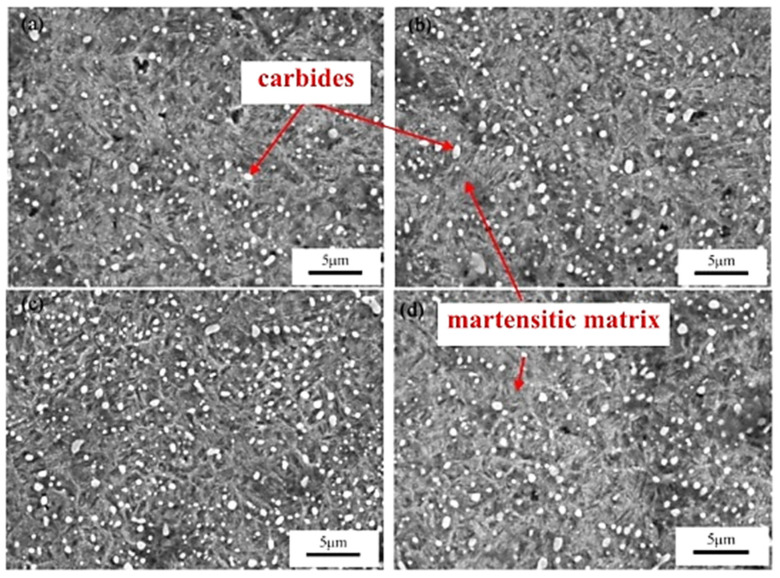
A series of secondary electron micrographs of AISI 52100 steel after (**a**) conventional heat treatment, (**b**) after one cryogenic cycle at −196 °C for 6 h, (**c**) after two cryogenic cycles, at −196 °C for 6 h each, and (**d**) after three cryogenic cycles, at −196 °C for 6 h each. Adapted from [207].

Properly heat-treated bearing steels contain a certain portion of secondary carbides (cementite) in their microstructures. Some remarks on the modification of secondary carbide populations in ball bearing steels due to cryogenic treatments were reported. Gunes et al. [45], Siva et al. [75], and Wang et al. [210] reported “higher volume fraction” and/or “better uniformity” of additional carbides in cryogenically treated bearing steels (at either −185 or −196 °C) but without providing relevant statistical data. On the other hand, Paydar et al. [173] quantified the increment of carbides counted in cryogenically treated (−196 °C/24 h) 100Cr6 steel to be 300–400%. Zhou et al. [207] thoroughly analysed secondary carbides in cryogenically treated (−196 °C/6 h, repeated cycles) AISI 52100 steel. The results show that the given CT refines the carbides and makes their population 60–70% higher. This is seen in a couple of SEM micrographs, Figure 12, where the area fraction of carbides increases with the number of cryogenic treatment cycles, up to a 68.6% increase [207].

### 3.3. Hot Work Tool Steels

**Figure 13:**
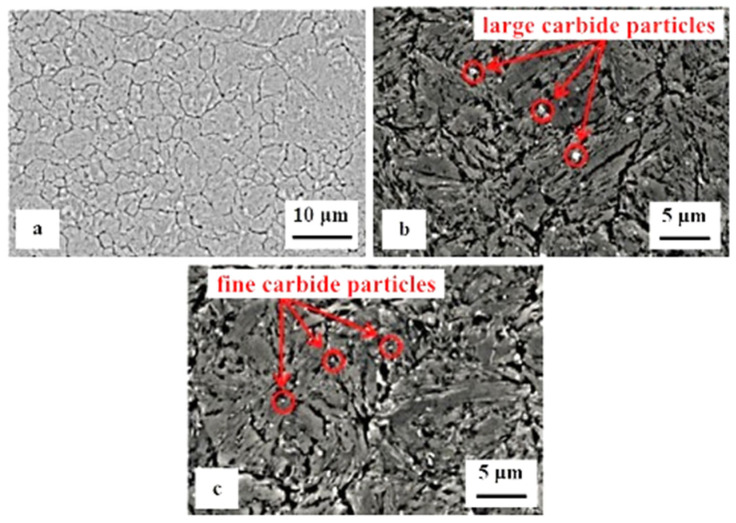
SEM micrographs showing as-quenched (**a**), as-tempered at 565 °C (**b**), and as-cryogenically treated at −145 °C for 24 h and tempered at 560 °C (**c**) microstructure of AISI H13 steel. Adapted from [253] (**a**) and [48] (**b**,**c**).

Hot work tool steels (see the overview in Table 3) are generally employed as tool materials in hot forging, die casting of lightweight metals, etc. They are usually used in a quenched and tempered condition, showing a martensitic matrix with the dispersion of fine precipitates of carbides. The steels are generally employed as tool materials in hot forging, die casting of lightweight metals, and other applications. Since the hot work tool steels usually contain only ~0.4% C (and ~5% Cr, ~1.4% Mo, ~0.9% V, and other minor elements), their susceptibility to maintain high amounts of retained austenite in as-quenched microstructures is expected to be low. Indeed, various investigators [251,252,253,254,255] have reported very low (up to 5 vol.%) or no presence of retained austenite in microstructures of hot work tool steels obtained by conventional quenching. On the contrary, there were some groups of investigators (e.g., [22,118,153,155,256,257]) who have reported significant amounts (up to 13%) of retained austenite after high-temperature pre-tempering prior to CT. This phase was eliminated only by subjecting pre-tempered specimens to a cryogenic cycle, followed by some post-tempering treatments (sequence *F*). Some of the most recent works were carried out via sequence *A*. In these works, the retained austenite was quantified in the prior-to-tempered state. However, the outcomes manifested clear differences. For instance, Li et al. [125] and Lopez-Leyva et al. [257] reported a significant reduction (but not complete elimination) of retained austenite, while Amini et al. [258] claimed complete elimination of this phase by cryogenic treatment at −196 °C. Figure 13 shows the differences between conventionally quenched, conventionally quenched and tempered, and cryogenically treated and tempered AISI H13 steel. It is seen that conventionally quenched and untempered steel contains well-visible primary austenite grains, whereas the microstructure is martensitic, shown in Figure 13a. High-temperature tempering evokes precipitation of nano-sized carbides inside the martensite, shown in Figure 13b. The application of cryogenic treatment enhances the number of precipitates and makes them finer overall, shown in Figure 13c.

Even though martensite refinement is one of the typical features of cryogenically treated steels, there is almost no evidence in the scientific literature for this phenomenon in hot work tool steels. One exception is the work by Koneshlou et al. [155], who mentioned that martensite laths are smaller and distributed more uniformly in the microstructure after holding the samples of AISI H13 steel for a long time at deep cryogenic temperatures.

The presence of additional small globular carbides was not identified in any available scientific papers, suggesting that this phenomenon is not associated with the cryogenic treatment of hot work tool steels. 

On the other hand, there is clear evidence of a higher number and a more uniform distribution of nano-sized precipitates in cryogenically treated and high-temperature tempered AISI H11 [122,259], H13 [51,118,125,199,256], and H21 [200] steels. The maximum populations of carbides were found for treatment durations between 16 and 24 h. However, it should be noted that this feature appeared in steel microstructures independent of the heat treatment strategy used. The use of sequence *A* had a very similar impact on the characteristics of precipitates as the use of strategies with pre-tempering prior to CT (*E* or *F*). This may be because the results were influenced/distorted by the pre-tempering of the materials prior to cryogenic treatment, which makes it impossible to judge the effect of the cryogenic treatment itself. 

### 3.4. Ledeburitic Steels and Eutectic Iron Alloys

**Figure 14:**
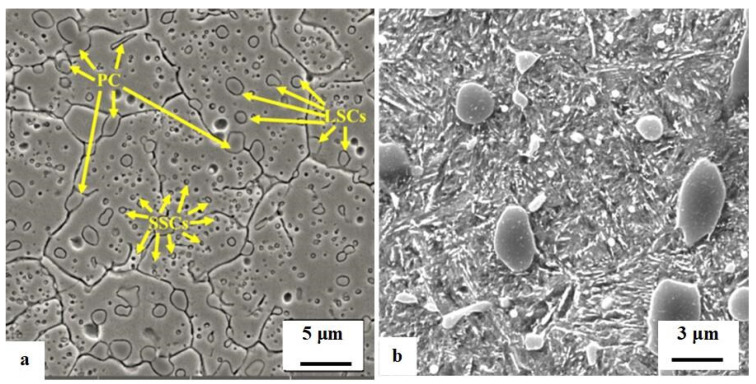
SEM images showing the microstructures of AISI D2 in an as-quenched state (**a**) and in an as-tempered state (**b**). The microstructure (**a**) was prepared by 2% Nital etching (adapted from [280]), and the microstructure (**b**) was pre-etched with Villela–Bain reagent (adapted from [308]). Legend: PC—primary carbides, LSCs—large secondary carbides, SSCs—small secondary carbides.

High-carbon, high-chromium, ledeburitic steels (the overview is in Table 4) were first developed as a substitute for high-speed steels, but they were found to be of limited use due to insufficient hot hardness. However, these steels proved useful in applications where high wear resistance and non-deforming properties were required, e.g., in cold-forming tooling [273,274,275]. The wear resistance can be improved further by adding vanadium into the alloys (high-carbon high Cr-V ledeburitic steels), producing hard primary or eutectic MC carbides. The steels involved in this class contain martensite, retained austenite (~20 vol.%), and undissolved carbides in their as-quenched microstructures [105,106,276], shown in Figure 14a. Subsequent tempering leads to the precipitation of nano-sized carbides, softening of the martensite, and in the case of high-temperature tempering, also to the decomposition of retained austenite [110,215,277], shown Figure 14b. However, some amount (up to ~5 vol.%) of retained austenite can be left in the steel microstructures even after 600 °C tempering [105].

**Table 4:** Overview of ledeburitic steels and eutectic iron alloys including their cryogenic treatment covered by this review showing key investigations carried out: M—microstructure (p in the column—includes phase transformations); A—retained austenite; C—carbide precipitation; H—hardness (t in the column includes tensile properties); N—notch/tooth root fracture resistance; K—fracture toughness; W—wear resistance and tribology; O—corrosion resistance. The designation “x” means that the particular microstructural feature/mechanical property was investigated in the referenced paper.

Steel Grade/Designation	Main Element Content (wt.%)	Conditions of Cryogenic Treatment	M	A	C	H	N	K	W	O	Reference
AISI D2	1.49 C, 11.48 Cr, 0.80 Mo, and 0.68 V	−75 °C/5 min; −125 °C/5 min; −196 °C/36 h	x			x		x	x		[12,53,56,278]
AISI D2	1.49 C, 11.48 Cr, 0.80 Mo, and 0.68 V	−196 °C/36 h or 84 h	x			x		x	x		[26,27]
AISI D2	1.49 C, 11.48 Cr, 0.80 Mo, and 0.68 V	−196 °C/5 min–132 h	x						x		[29]
AISI D2	1.49 C, 11.48 Cr, 0.80 Mo, and 0.68 V	−75 or −125 °C/5 min; −196 °C/5 min–84 h	x						x		[54,55]
AISI D2	1.55 C, 12 Cr, 0.8 Mo, and 0.9 V	−70, −100 −130, or −196 °C/2, 4, 8, 18, 24, or 48 h	x	x		x	x		x		[81]
AISI D2	1.55 C, 11.3 Cr, 0.8 Mo, and 0.8 V	−193 °C/24 h	p								[98]
AISI D2	1.55 C, 12 Cr, 0.8 Mo, and 0.9 V	−40, −100, or −196 °C/38 min–20 h	x	x		x					[156]
AISI D2	N/A	−90, −120, or −150 °C/25 min or 24 h	x								[157]
AISI D2	1.55 C, 11.3 Cr, 0.8 Mo, and 0.8 V	−196 °C/5 min, 29 h, or 72 h	x	x	x						[195]
AISI D2	1.5 C, 12 Cr, 0.5 V, and 0.4 W	−196 °C/48 h	x						x		[212]
AISI D2	1.51 C, 11.39 Cr, 0.84 Mo, and 0.25 V	−196 °C/20 h	x	x	x	x	x		x		[279]
AISI D2	1.54 C, 11.67 Cr, 0.75 Mo, and 0.93 V	−196 °C/4 h	x			x		x			[280]
AISI D2	1.54 C, 11.88 Cr, 0.76 Mo, and 0.75 V	−196 °C/4 h	x			t					[281]
AISI D2	1.59 C, 11.84 Cr, 0.8 Mo, and 0.95 V	−185 °C/36 h				x			x		[282]
AISI D2	1.58 C, 11.51 Cr, 0.9 Mo, and 0.74 V	−160 °C/5, 10, or 15 h	x			x			x		[283]
AISI D2	1.55 C, 11.3 Cr, 0.8 Mo, and 0.8 V	−145 °C/4 or 24 h	x			x					[284]
AISI D2	1.47 C, 11.54 Cr, 0.8 Mo, and 0.23 V	−196 °C/5 min	x	x		x					[285]
AISI D2	1.55 C, 11.5 Cr, 0.9 Mo, and 0.68 V	−196 °C/12 h	x	x	x	x		x			[286]
X153CrMoV12	1.55 C, 11.90 Cr, 0.70 V, and 0.85 Mo	−150 or −196 °C/15 min—24 h; −170 °C/15–240 min; −100 °C/30 min	p	x	x						[119]
X153CrMoV12	1.55 C, 11.90 Cr, 0.70 V, and 0.86 Mo	−150 or −196 °C/15 min—24 h	p								[205]
X153CrMoV12	1.55 C, 11.90 Cr, 0.70 V, and 0.86 Mo	−150 or −196 °C/24 h	x								[206]
X153CrMoV12	1.4 C, 12 Cr, 0.8 Mo, and 0.7 V	−140 °C/120 min	x			x			x		[287]
X153CrMoV12	1.52 C, 11.38 Cr, 0.87 Mo, and 0.85 V	−120 °C/20 h or −196 °C/40 h	x			x		x	x		[288]
X155CrVMo121	1.55 C, 11.55 Cr, 0.88 V, and 0.8 Mo	−40, −80, −130, or −196 °C/15 min		x		x					[32]
X155CrMoV12 1	1.55 C, 11.5 Cr, 0.7 Mo, and 1.0 V	−196 °C/14 or 35 h				x			x		[289]
X165CrV12	1.6 C, 11.65 Cr, and 0.5 V	−40, −80, −130, or −196 °C/15 min		x		x					[32]
X165CrCoMo12	1.71 C, 11.1 Cr, 0.96 Co, and 0.56 Mo	−40, −80, −130, or −196 °C/15 min		x		x					[32]
1.4C12CrMoV	1.44 C, 12.2 Cr, 0.84 Mo, and 0.43 V	−50 or −180 °C/duration N/A	x						x		[120]
AISI D3	2.2 C, 12 Cr, 0.5 V, and 0.4 W	−195 °C/24 or 48 h	x			x					[14]
AISI D3	2.2 C, 12 Cr, 0.5 V, and 0.4 W	−196 °C/36 h	x			x		x			[25,290]
AISI D3	2.00–2.35 C, 11.00–13.50 Cr, and 1 V	−196 °C/10 h	x			x			x		[40]
AISI D3	2.09 C and 12.35 Cr	−196 °C/36 or 84 h	x								[41]
AISI D3	2.2 C, 12 Cr, 0.5 V, and 0.4 W	−195 °C/24, 36, or 48 h	x						x		[159,161]
AISI D3	2.2 C, 12 Cr, 0.5 V, and 0.4 W	−196 °C/8 or 24–120 h	x			x					[160,162]
AISI D3	1.8 C, 12 Cr, 1 W, and 0.5 V	−195 °C/24, 36, or 48 h	x							x	[291]
AISI D3	1.98 C, 12.78 Cr, and 0.4 V	−185 °C/8 h	x			x			x		[292]
AISI D3	N/A	−180 °C/6 h or −110 °C/24 h or −140 °C/24 h							x		[293]
AISI D3	2.0 C and 10.43 Cr	−196 °C/12, 24, or 36 h	x			x		x			[294]
AISI D3	2.07 C and 12.4 Cr	−196 °C/24 h	x							x	[247]
AISI D3	2–2.3 C, 11–13.5 Cr, and 1 V	−145 °C/24 or 36 h								x	[295]
AISI D5	1.52 C, 11.57 Cr, 0.88 Mo, and 2.99 Co	−185 °C/36 h	x			x				x	[296]
AISI D6	2–2.3 C, 11–13 Cr, and 0.6–0.8 W	−180 °C/24 h	x						x		[13]
AISI D6	2.1 C, 12 Cr, and 0.748 W	−63 °C/20 or 40 h, or −196 °C/10 h	x			x			x		[78]
AISI D6	2.1 C, 12 Cr, and 0.748 W	−63 °C/20 h	x			x			x		[158]
X210Cr12	2.05 C and 12.09 Cr	−40, −80, −130, or −196 °C/15 min		x		x					[32]
X210CrW12	2.13 C, 12.18 Cr, and 0.88 W	−40, −80, −130, or −196 °C/15 min		x		x					[32]
X190CrVMo20-4	1.99 C, 19.70 Cr, 1.05 Mo, and 4.02 V	−120 °C/20 h or −196 °C/40 h	x			x		x	x		[288]
X190CrVMo20-4	1.99 C, 19.70 Cr, 1.05 Mo, and 4.02 V	−196 °C/15 min	x							x	[297]
X220CrMoV13-4	2.2 C, 13 Cr, 4 V, and 1 Mo	−150 or −196 °C/15 min–24 h; −170 °C/15–240 min; −100 °C/30 min	p	x	x						[119]
X220CrVMo13-4	2.2 C, 13 Cr, 4 V, and 1 Mo	−150 or −196 °C/24, 36, or 48 h	x								[184]
X290Cr12	2.97 C and 11.46 Cr	−40, −80, −130, or −196 °C/15 min		x		x					[32]
Vanadis 6	2.1 C, 6.8 Cr, 1.5 Mo, and 5.4 V	−75 °C/4–48 h	x		x	x		x			[15]
Vanadis 6	2.1 C, 6.8 Cr, 1.5 Mo, and 5.4 V	−140 °C/4–48 h	p		x	x					[21]
Vanadis 6	2.1 C, 6.8 Cr, 1.5 Mo, and 5.4 V	−196 °C/4 or 10 h, or −90 °C/4 or 10 h	x			x		x	x		[28]
Vanadis 6	2.1 C, 6.8 Cr, 1.5 Mo, and 5.4 V	−196 °C/4 h	x			x					[30,179]
Vanadis 6	2.1 C, 6.8 Cr, 1.5 Mo, and 5.4 V	−269 °C/17 h	x			x					[35,298]
Vanadis 6	2.1 C, 6.8 Cr, 1.5 Mo, and 5.4 V	−75, 140, −196, or −269 °C/17 h	x	x						x	[36]
Vanadis 6	2.1 C, 6.8 Cr, 1.5 Mo, and 5.4 V	−196 °C/4–48 h	x			x					[57]
Vanadis 6	2.1 C, 6.8 Cr, 1.5 Mo, and 5.4 V	−196 °C/4 or 17 h	p		x	x		x			[58]
Vanadis 6	2.1 C, 6.8 Cr, 1.5 Mo, and 5.4 V	−140 °C/17 h	x			x		x			[59]
Vanadis 6	2.1 C, 6.8 Cr, 1.5 Mo, and 5.4 V	−196 °C/4 or 10 h; −90 °C/4 h	x			x					[60]
Vanadis 6	2.1 C, 6.8 Cr, 1.5 Mo, and 5.4 V	−196 °C/10−48 h	x	x	x	x					[116]
Vanadis 6	2.1 C, 6.8 Cr, 1.5 Mo, and 5.4 V	−196 °C/24 h	x			x		x			[299]
Vanadis 6	2.1 C, 6.8 Cr, 1.5 Mo, and 5.4 V	−196 °C/4 or 10 h, or −90 °C/4 or 10 h	x			x			x		[300]
Vanadis 6	2.1 C, 6.8 Cr, 1.5 Mo, and 5.4 V	−75, 140, or −196/17 h	x	x		x			x		[301,302]
Vanadis 8	2.3 C, 4.8 Cr, 3.6 Mo, and 8 V	−140 °C/24 h	x	x		x					[303]
HVAS steel	1.95 C, 6.1 Cr, 7.3 V, 5.1 Mo, and 3.1 Ni	−196 °C/12, 24, or 48 h	x			x		x	x		[304]
X110CrMoV8 2	1.10 C, 8.30 Cr, 2.10 Mo, and 0.50 V	−196 °C/14 or 35 h				x			x		[211]
DC53	0.91 C, 8.6 Cr, 1.47 Mo, and 0.5 V	−196 °C/4 h	x								[194]
DC53	0.91 C, 8.6 Cr, 1.47 Mo, and 0.5 V	−196 °C/40 h	x								[201,202]
DC53	0.91 C, 8.6 Cr, 1.47 Mo, and 0.5 V	−196 °C/40 h	p								[203]
DC53	0.98 C, 8.6 Cr, 2 Mo, and 0.5 V	−196 °C/2 or 24 h	x	x		x	x				[305]
Sleipner	0.9 C, 7.7 Cr, 2.5 Mo, and 0.5 V	−180 °C/24 h	x	x		x					[248]
Sleipner	0.9 C, 7.7 Cr, 2.5 Mo, and 0.5 V	−80 °C/12 or 24 h; 180 °C/12–36 h	x			x			x		[306]
16Cr1Mo1Cu	2.77 C, 16.38 Cr, and 0.9 Cu	−196 °C/3 h	x						x		[307]

An overview of ledeburitic steels and/or eutectic iron alloys and their cryogenic treatments incorporated in this review can be found in Table 4. Even though all of the trials available in the scientific literature were conducted by using sequence *A*, the data on retained austenite reduction are inconsistent. Das et al. [12,26,27,29,53,54,55,278] reported almost complete removal of retained austenite from the AISI D2 steel after cryogenic treatment at −125 °C and below. Very similar results were published by Akhbarizadeh and his collaborators for AISI D3 and D6 steels [14,25,159,160,161,162,212,291] and by Kumar et al. [294] for AISI D3 steel. On the other hand, many investigators have pointed out that even though the application of CT reduces the RA amount in different steels, namely, AISI D2 [120,157,279,280], DC53 [201,202,203], X220CrVMo13-4 [184], or Vanadis 6 [21,30,36,59,116], some measurable amounts of RA are left in the steels (up to ~5 vol.% in most cases). Moreover, the reduction in RA manifested a clear indication of time-dependency in the case of AISI D2 steel [12], AISI D6 steel [78], or Vanadis 6 steel [15,21,116].

The formation of refined martensite was also reported for cryogenically treated ledeburitic cold work tool steels. One of the works where martensitic domain refinement was identified was the paper by Tyshchenko et al. [184]. Martensite refinement by visual inspection of TEM images of differently cryogenically treated Vanadis 6 steel was also recorded in investigations of Jurci et al. [21,30]. It should be mentioned that an exact quantification of this phenomenon is practically impossible, mainly due to small transparent areas in thin foils made for TEM observations.

As mentioned above, there is a great scientific debate on the presence of additional small globular carbides in cryogenically treated steels. The class of ledeburitic tool steels is the most typical example where the presence of these particles has been proven. Starting with findings by Collins and his co-workers [81,156,309], through to careful and statistically conclusive examinations by Das et al. [12,26,27,29,53,54,55,56,278], and up to some independent reports by Moscoso et al. [280], Surberg et al. [157], Akcinoglu et al. [287], or Ghasemi-Nanesa et al. [281], there is strong evidence for the increment of carbide particles in AISI D2 steel resulting from CT by using sequence *A*. These increments may be several tens of per cent [280] but also hundreds of per cent [12,53], depending on the temperature of cryogenic treatment and its duration. It is also worth noting that these results have been used to explain the dramatic increase in the wear performance of D-class tool steels treated in this way, as determined either by laboratory examinations [13,14,25,158,159,160,282,290,292,293,294] or by industrial tests [38,39,40]. 

**Figure 15:**
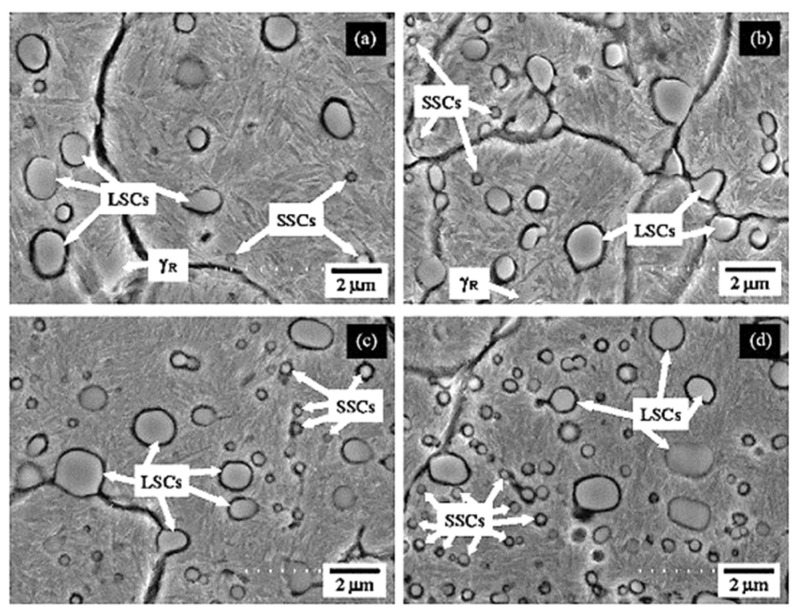
Typical SEM micrographs of steel that is (**a**) conventionally heat treated, (**b**) cryogenically treated at −75 °C for 5 min, (**c**) cryogenically treated at −125 °C for 5 min, and (**d**) cryogenically treated at −196 °C for 36 h. Note that secondary carbides are denoted here as LSCs and additional SGCs as SSCs, and γ_R_ is the retained austenite. Adapted from [12].

**Figure 16:**
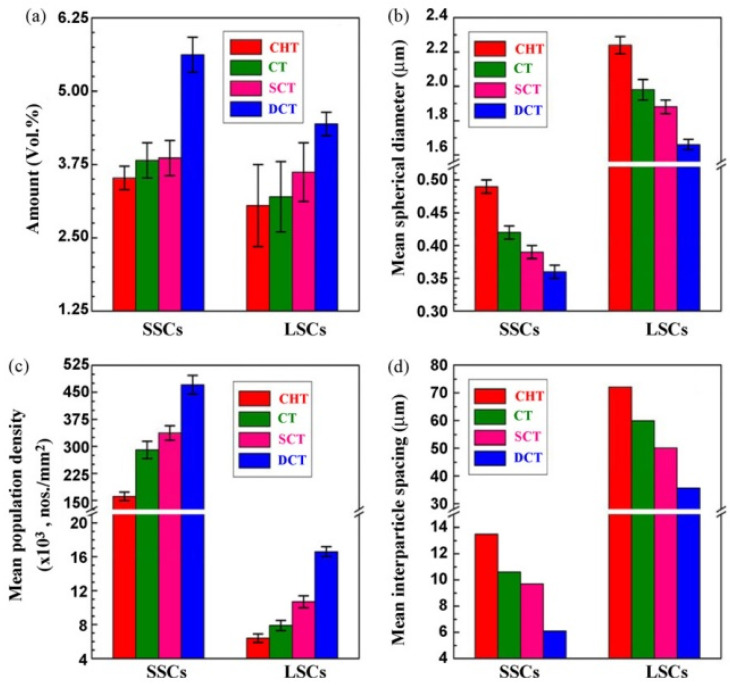
Results of image analyses of carbides in AISI D2 steel with different cryogenic treatments: (**a**) amount, (**b**) mean spherical diameter, (**c**) mean population density, and (**d**) mean interparticle spacing of additional small globular carbides (here, denoted as small secondary carbides, SSCs) and secondary carbides (here, denoted as large secondary carbides, LSCs) in conventionally heat treated (CHT), cryogenically treated at −75 °C (here, denoted as cold treated, CT), cryogenically treated at −125 °C (here, denoted as shallow cryogenically treated, SCT), and cryogenically treated at −196 °C (here, denoted as deep cryogenically treated, DCT) specimens. Adapted from [12].

Figure 15 provides clear evidence of carbide’s increment in AISI D2 steel due to cryogenic treatments. SEM images of the steel that experienced CHT (a) and CT under different conditions (b–d) [12] show that the matrix is mainly martensitic, with small amounts of retained austenite (visible only in Figure 15a). Two types of carbides are present in the microstructures—namely, secondary carbides (denoted as LSCs) and additional small globular carbide particles (here, denoted as SSCs). A comparison of the micrographs in Figure 15 reveals that the population density of SSCs increases with decreasing CT temperature. Results of image analyses, shown in Figure 16, confirm that CT increases the amount and population density of carbide particles and makes them finer overall [12]. Conversely, the interparticle spacing of carbides decreases with the application of cryogenic treatments. The maximum carbide counts were determined after 36 h of treatment in liquid nitrogen, while the use of higher CT temperatures resulted in lower carbide populations [53]. 

**Figure 17:**
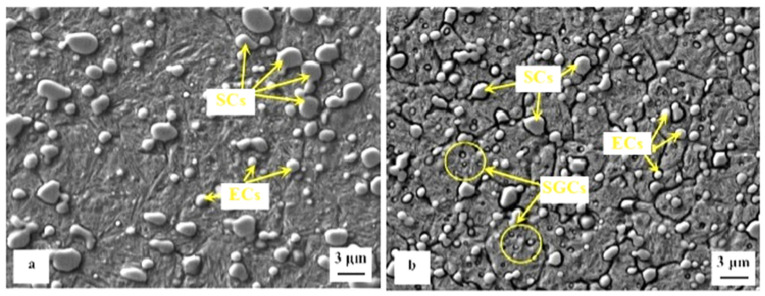
Scanning electron micrographs showing the microstructure of prior-to-tempered Vanadis 6 steel after conventional heat treatment (untampered) (**a**) and after cryogenic treatment at −140 °C for 17 h (**b**). The ECs, SCs, and SGCs are the eutectic, secondary, and small globular carbides, respectively.

**Figure 18:**
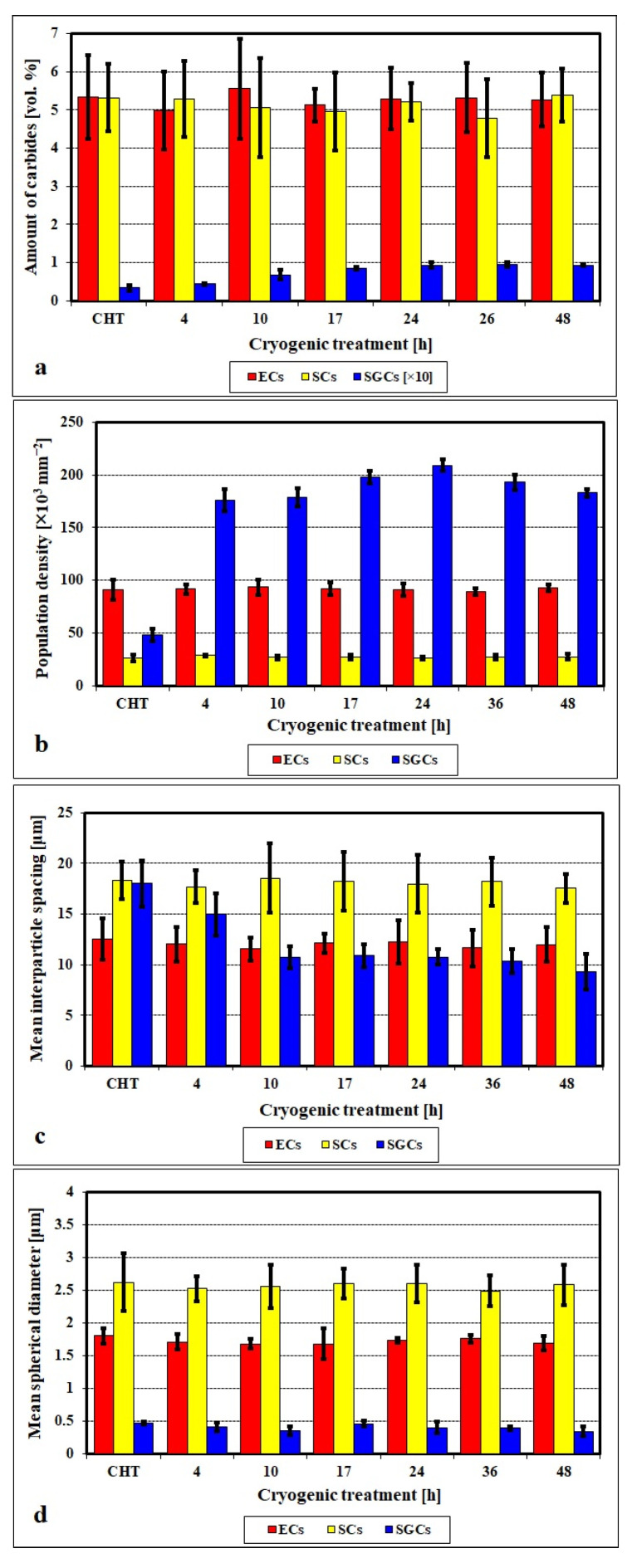
Dependence of (**a**) the number of different carbides, (**b**) their population density, (**c**) interparticle spacing, and (**d**) the mean spherical diameter on the duration of cryogenic treatment at −140 °C for Vanadis 6 steel in the prior-to-tempered state. The ECs, SCs, and SGCs are the eutectic, secondary, and small globular carbides, respectively. Adapted from [21].

**Figure 19:**
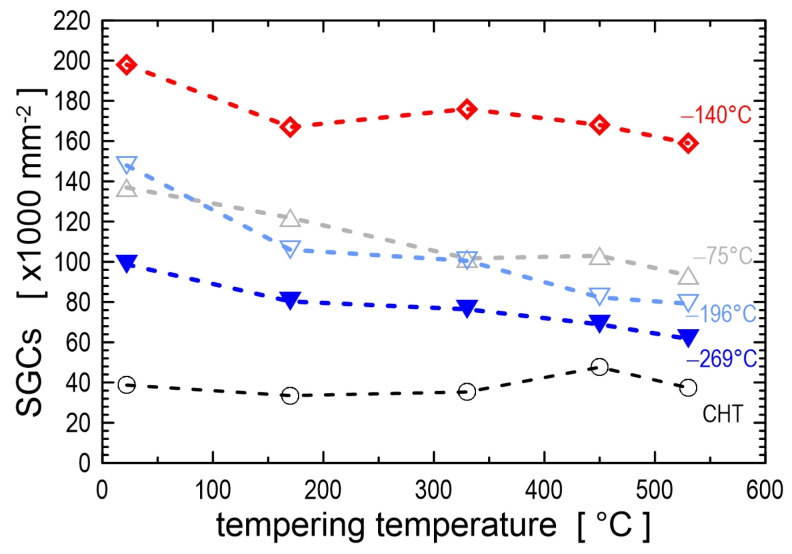
The effect of the cryo-temperature (shown on separate curves) and the tempering temperature (horizontal axis) on the population density of small globular carbides (SGCs, vertical axis) in cryogenically treated and differently tempered Vanadis 6 steel specimens. Cryogenic treatments were carried out for 17 h, while tempering was performed twice, each cycle for 2 h. The values are from published studies as follows: CHT—conventional heat treatment [15,21,116], −75 °C [15], −140 °C [21], −196 °C [30,116], and −269 °C [35].

Examinations of cryogenically treated AISI D3 [14,25,159,160,161,162,292,294], AISI D6 [78,158], or Vanadis 6 steel [15,21,36,116,179] gave very similar results, including the finding of temperature- and time-dependency for the extent of carbide count increase. Figure 17 demonstrates that the additional SGCs are present in the microstructures of cryogenically treated Vanadis 6 prior to tempering [21]. Details of quantitative microstructural analyses of carbides in CT (at −140 °C, for different durations) are shown in Figure 18. It is seen that cryogenic treatment does not modify the characteristics of eutectic and secondary carbides (Figure 18a) but increases the amount of additional small globular carbides (Figure 18b), with the maximum value at 17–24 h of treatment. Conversely, the dependence of the mean interparticle spacing on the CT duration (Figure 18c) manifests an opposite tendency, since this characteristic is inversely proportional to the number of particles. And finally, the effect of CT on the mean spherical diameter of all of the carbide classes is minimal, shown in Figure 18d. As a summary, the diagram in Figure 19 reflects the significant effect of the cryo-temperature on the overall level of small globular carbides and shows the highest population density for CT at −140 °C and also that the number of SGC particles tends to decrease with tempering [15,21,30,35,116]. 

It should be noted, however, that there are scientific works where no “additional” carbides have been reported. This especially concerns the AISI D2 steel, where Meng et al. [120], Pellizzari and Molinari [211], Gavriljuk et al. [119,205], Pellizzari et al. [195], and Villa et al. [98] did not mention any variations in carbide characteristics after cryogenic treatments.

**Figure 20:**
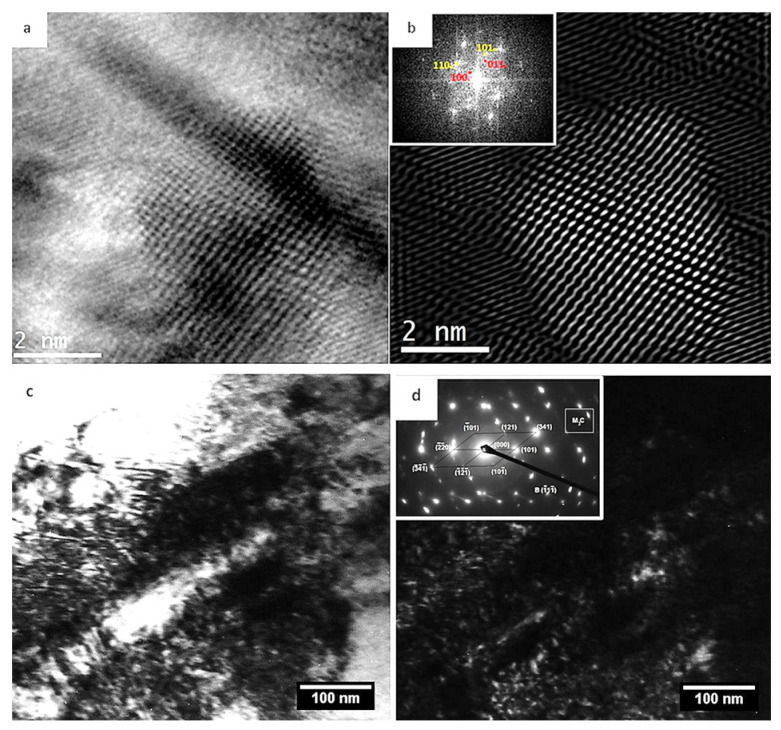
High-resolution transmission electron micrographs showing ε-carbide (**a**,**b**) and TEM micrographs of cementite (**c**,**d**) precipitate nano-particles in cryogenically treated (at −140 °C for 17 h) and prior-to-tempered Vanadis 6 steel: (**a**) bright-field image and (**b**) dark-field image with corresponding diffraction patterns of ε-carbide; (**c**) bright-field image and (**d**) corresponding dark-field image with diffraction patterns of cementite. Image 20a,b are adapted from [21].

Precipitation of transient and stable carbides during tempering of cryogenically treated steels has been extensively studied for AISI D2 and Vanadis 6 steel. The examinations of AISI D2 steel gave contradictory results. Meng et al. [120] reported that cryogenic treatment at −196 °C accelerated the precipitation of transient η-carbides and made their distribution more homogeneous, while Gavriljuk et al. [119,206] claimed the opposite results after cryogenic treatment at either −150 or −196 °C. In the case of Vanadis 6 steel, the results indicate an acceleration of the precipitation kinetics of transient carbides at low tempering temperatures but suppression of precipitation of stable carbides at high tempering temperatures, around 500 °C [21,116,179,206]. Figure 20 shows examples of nano-sized ε-carbides and cementite particles found in cryogenically treated (prior-to-tempered) Vanadis 6 steel. In conventionally heat-treated samples of the same steel, the nano-sized transient carbides were not found in the prior-to-tempered state but only after low-temperature tempering. 

### 3.5. High-Speed Steels

High-speed steels (an overview of examined steels is in Table 5) are used for applications requiring long life at relatively high operating temperatures, such as for heavy cuts or high-speed machining. They contain relatively high amounts of carbon (around 1 wt.%), Cr (typically 4 wt.%), tungsten, molybdenum, and vanadium; some of them also contain cobalt. High-speed steels are the most important tool steels in metal cutting applications because of their very high hardness and good wear resistance in the heat-treated condition and their ability to retain high hardness at the elevated temperatures often encountered during the operation of the tool at high cutting speeds. 

All high-speed steels are rated in the ledeburite class. In the cast state, they have the structure of white hypoeutectic cast iron [96]. Since the high-speed steels are austenitised at very high temperatures, their austenite is highly saturated with carbon and alloying elements resulting from the dissolution of eutectoid and secondary carbides. The M_f_ temperature of these steels lies far below zero degrees Celsius, and the materials may contain more than 20 vol.% of retained austenite in their as-quenched microstructures [63,64,114]. Other microstructural constituents are martensite and different undissolved carbides. In conventional heat treatment, the as-quenched steels are subjected to several tempering cycles (at around 550 °C), which leads to almost complete retained austenite removal and precipitation of carbide nano-particles (secondary hardening effect) [96]. 

An alternative way to reduce the retained austenite in high-speed steels is by applying cryogenic treatment. For instance, Leskovšek et al. [63] reported almost complete removal of retained austenite due to cryogenic treatment at −196 °C (sequence *A*) for AISI M2, AISI M35, and AISI M3:2 grades. Similar effects have also been recorded by Yun et al. for W6Mo5Cr4V2 steel [64] and by Candane et al. [170] for AISI M2 steel. If the temperature of −70 °C was applied for treatment, then around 7 vol.% of retained austenite was left in the microstructure of AISI M2 steel. However, prior-to-CT tempering (sequence *F*) leads to stabilisation of the RA, and subsequent CT at −70 °C is less effective in RA reduction [114]. And finally, several investigators have compared the RA amounts after conventional heat treatment and CT followed by tempering, and they did not record any significant differences [164,166,167,168], suggesting that CT has only a minimal effect on RA when the steels are subsequently high-temperature tempered.

**Figure 21:**
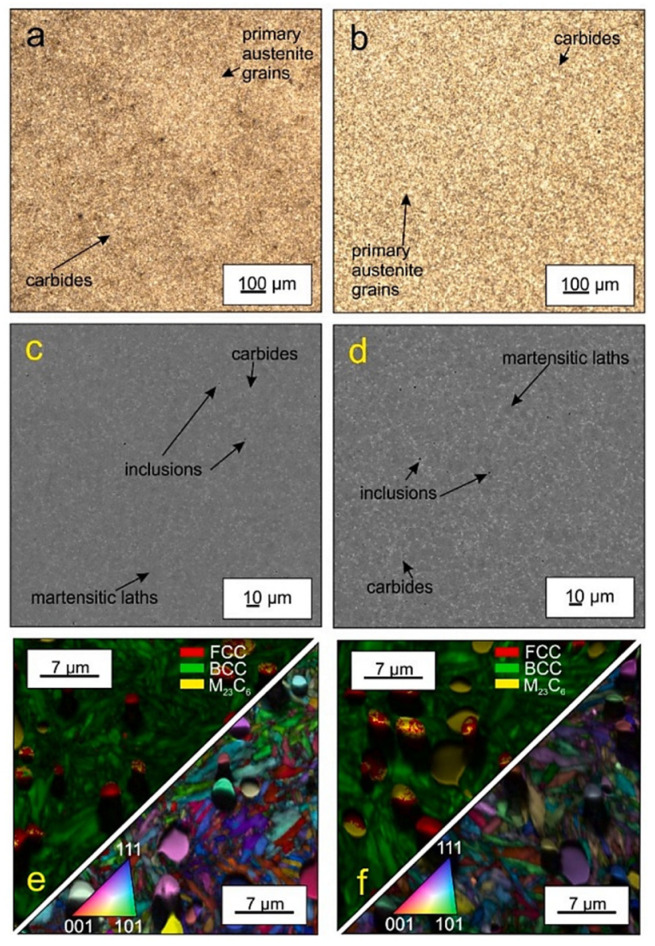
Images obtained by light microscope (**a**,**b**), scanning electron microscope by secondary electrons (**c**,**d**), and electron backscattered diffraction (EBSD) and orientation (inverse pole figures) (**e**,**f**) for AISI M3:2 high-speed steel after conventional heat treatment (**a**,**c**,**e**) (austenitisation at 1180 °C for 2 h followed by 3 cycles of 540 °C tempering) and after cryogenic treatment (**b**,**d**,**f**) at −196 °C for 4 h, sequence *A*. Adapted from [208].

Various authors have detected a more or less significant refinement of martensite in high-speed steels due to cryogenic treatments, mostly carried out at −196 °C. This finding concerned the most popular AISI M2 grade [67,185,208], W9Mo3Cr4V steel [10], 5% Co-containing AISI M35 grade [67,186,208], and PM grade AISI M3:2 [168,208]. Figure 21 provides clear evidence of microstructural refinement and better carbide uniformity in an example of powder metallurgy AISI M3:2 steel due to the application of cryogenic treatment at −196 °C for 24 h. Light microscope images (Figure 21a,b) provide only an overall picture of the microstructure of CHT and cryogenically treated steel. Both microstructures are martensitic, with clearly visible primary austenite grains boundaries and with the presence of fine and uniformly distributed carbides. Secondary electron images (Figure 21c,d) show that the carbides are mostly spherical and are identified as MC, M_6_C, and M_2_C (eutectic carbides) and M_23_C_6_ (secondary carbides) [208]. In cryogenically treated steel, the carbides are more homogenously distributed and their volume fraction is determined to be increased by about 4%, as compared with CHT specimens. The EBSD results (Figure 21e,f) show that the matrix is lath martensite and contains different carbides. On average, martensitic laths in the CHT samples are 10% larger than those found in cryogenically treated specimens [208]. EBSD also indicates that no retained austenite is present in either CHT or CT specimens. This is due to high-temperature tempering that was used as the final heat treatment step in both cases. The martensite laths orientation of the CHT specimens is mainly random, while the laths are oriented mostly along the [101] and [001] directions in cryogenically treated specimens.

**Figure 22:**
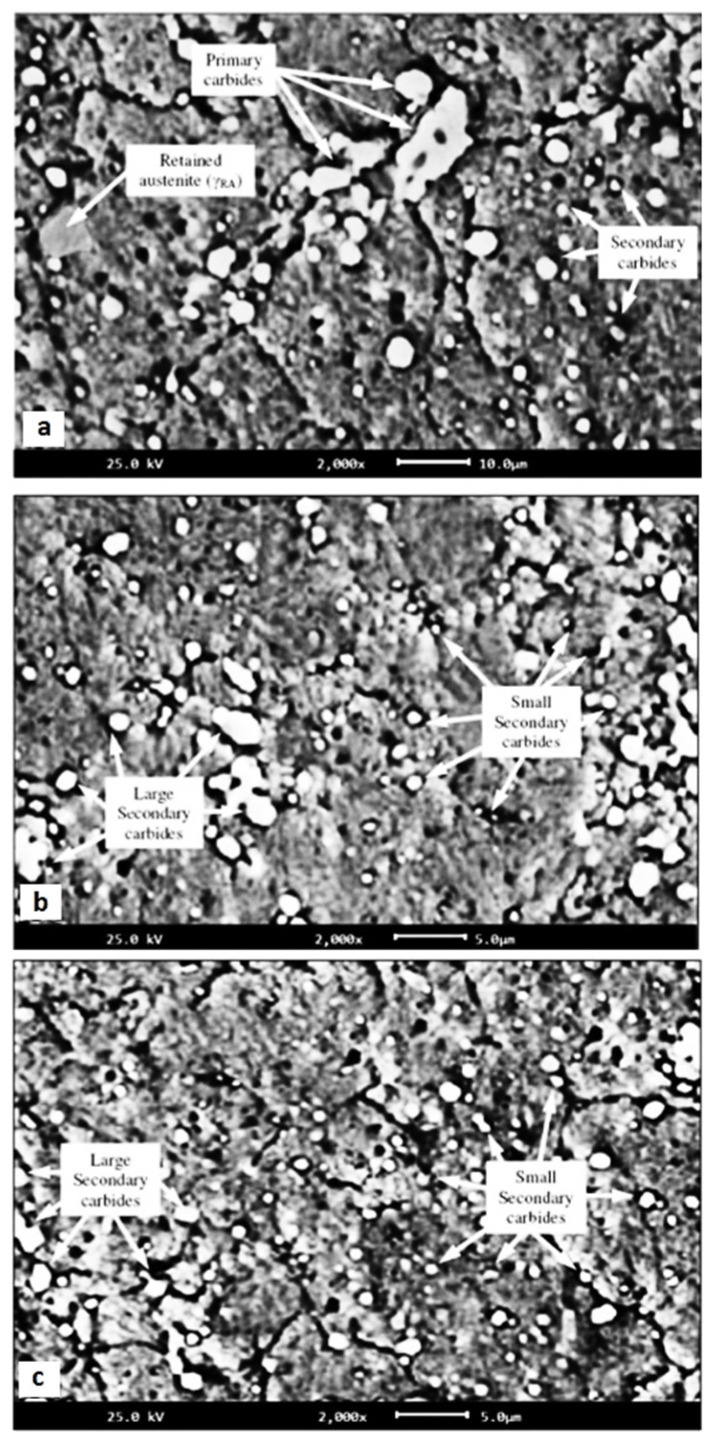
SEM microstructures of AISI M2 steel after (**a**) conventional heat treatment (austenitising at 1210 °C and tempering at 150 °C), (**b**) cryogenic treatment at −110 °C for 18 h (sequence *A*), and (**c**) cryogenic treatment at −196 °C for 38 h (sequence *A*). Adapted from [115].

Numerous studies have focussed on determining the changes in carbide counts in high-speed steels due to CT. Experimental investigations carried out on AISI M2 steel (treated following sequence *A*, after high-temperature tempering) [61,67,115,169,208] have produced great variability in terms of the carbide populations obtained, an increase from 25% to 100%. However, it is undeniable that a lower cryogenic temperature (e.g., −196 °C) increases the carbide number more effectively than treatment at −120 or −70 °C [65,209]. There is no clear consensus on the optimal duration of cryogenic treatment. Some authors recommend a duration in the range of 4–12 h [65,310], while others have achieved the highest carbide counts with a 24 h treatment [61,67,115,208]. The three SEM images in Figure 22 show the microstructures of conventionally heat treated AISI M2 steel and the same steel after being subjected to cryogenic treatments at either −110 or −196 °C (for 4 h both), following sequence *A*. All of the microstructures reveal the strong presence of carbide particles in the tempered martensite matrix. The carbides are eutectic particles (here, denoted as “primary”) and secondary carbides. Moreover, cryogenically treated steel contains an enhanced number of additional SGCs (here, denoted as “small secondary carbides”). Still, the carbides are not evenly distributed throughout the bulk of the material after cryogenic treatment at −110 °C. Meanwhile, the microstructure of −196 °C-treated steel reveals the presence of SGCs which are evenly distributed in the entire bulk of the material and their number is much higher than in the case of −110 °C-treated steel.

Examinations of other high-speed steels, such as AISI M3:2 [61,170], AISI M35 [61,256,311,312,313], AISI T42 [209,311], or S390 Microclean [165], have not provided a specification for the optimal combination of CT parameters to obtain the highest possible number of carbides because the results differ greatly. Moreover, no effect of CT on the carbide population was reported in some cases for AISI M3:2, S390 Microclean [167,168], or AISI M35 grade [121].

**Figure 23:**
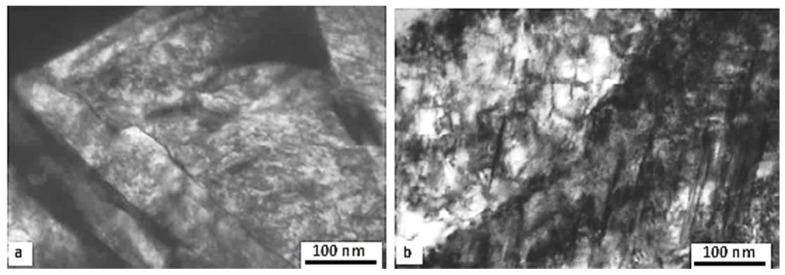
TEM images of AISI M2 steel after (**a**) conventional hardening (austenitising at 1200 °C and quenching) and (**b**) subsequent cryogenic treatment at −180 °C for 24 h. Adapted from [185].

Few studies have focussed on an attempt to quantify nano-sized carbide precipitation by using TEM. Jovičevič-Klug et al. [61] concluded that cryogenic treatments at −196 °C increased the density of precipitation of carbides up to 30% in AISI M2, AISI M3:2, and AISI M35 steels. In another study, Yun et al. [64] and Jeleńkowski et al. [185] examined the precipitation of carbides in cryogenically treated W6Mo5Cr4V2 and W18Cr4V high-speed steels. They arrived at the general observation of accelerated precipitation of nano-sized carbide particles due to treatments at either −80 or −196 °C. The same applies to AISI M3:2 steel after cryogenic treatments at −196 °C for 24 h, where an increased precipitation rate of M_23_C_6_ nano-sized carbides was established [314]. The two TEM images in Figure 23 show the microstructures of HS 6-5-2 (AISI M2) steel after being subjected to a hardening procedure from 1200 °C (a) and after subsequent cryogenic treatment at −180 °C for 24 h (b). In the matrix of the conventionally hardened sample, there are clusters–globules with a diameter of 10–15 nm located at dislocations, and plates situated at the grain boundaries and within the martensite twins, with a thickness of about 10–15 nm. In sample after CT, the globules and plates have clearly defined contours. Local configurations of some plates resemble the morphology of the tweed-like structure, i.e., the morphology of precipitations formed by the spinodal decomposition. This finding may indicate an accelerated precipitation of transient carbides in cryogenically treated steel, which is demonstrated by the presence of spinodal decomposition, while no presence of such a decomposition is present in CHT steel.

### 3.6. Martensitic Stainless Steels

Martensitic stainless steels (an overview of the steels examined here is in Table 6) are widely used in the manufacture of mechanical bearings in automotive engines, gas turbines, and aerospace vehicles. Traditional bearing steel often fails in these applications because of complex and variable environments such as impact stress, high temperature, wear, and corrosion [319]. Therefore, properties such as high temperature and corrosion resistance are the key factors for service. However, it was found that martensitic stainless steels often contain more retained austenite after quenching and tempering due to the high-alloying element content [320]. The main reason for cryogenic treatment is a reduction in retained austenite because it is metastable and can easily transform into brittle martensite, resulting in a deviation in the size of the workpieces or their failure.

**Figure 24:**
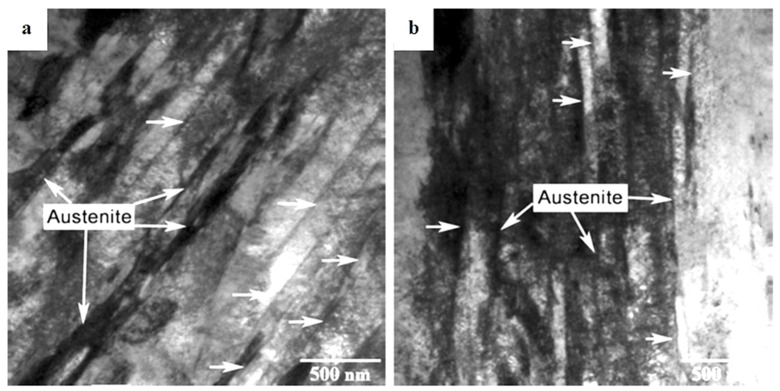
TEM microstructures of retained austenite in 0.15% C, 14% Cr, 12.5% Co, 4.5% Mo, and 2% Ni steel after different heat treatment processes. (**a**) Austenitisation at 1050 °C for 40 min, followed by oil quenching; (**b**) austenitisation at 1050 °C for 40 min, followed by oil quenching and CT at −196 °C for 2 h. Adapted from [70].

**Figure 25:**
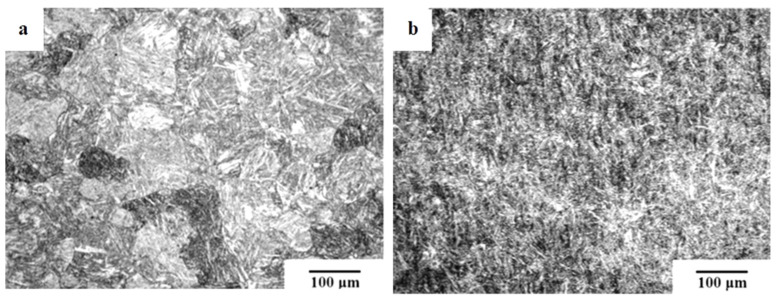
Optical microstructures of 0.15% C, 14% Cr, 12.5% Co, 4.5% Mo, and 2% Ni steel after different heat treatment processes. (**a**) Austenitisation at 1050 °C for 40 min, followed by oil quenching; (**b**) austenitisation at 1050 °C for 40 min, followed by oil quenching and CT at −196 °C for 2 h. Adapted from [70].

**Figure 26:**
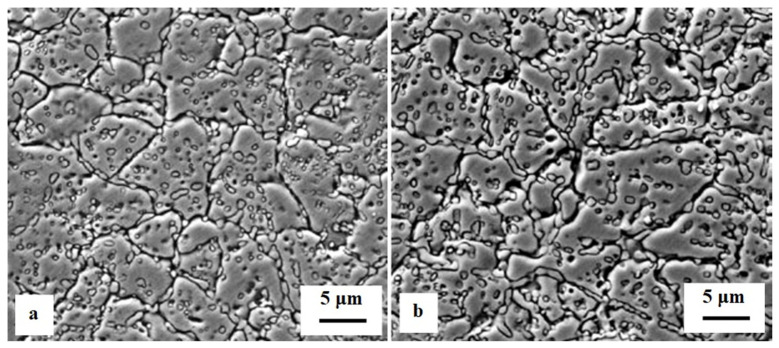
SEM images of conventionally oil-quenched from the austenitisation temperature of 960 °C for 30 min (**a**), and cryogenically treated (at −196 °C for 24 h) (**b**) AISI 420 martensitic stainless steel. Adapted from [210].

After conventional quenching, the retained austenite amounts often exceed 20 vol.% in steels containing different carbon and alloying elements [16,17,69,70,71]. Cryogenic treatment is an effective way to reduce retained austenite to an acceptable level. For instance, it was found that CT at −196 °C (following sequence *A*) reduced the RA from 26 vol.% to one tenth of this value in the case of the steel with 0.15% C, 14% Cr, 13% Co, 2.4% Ni, and 4.8% Mo [69]. Similar results were also obtained through cryogenic treatment of X30CrMoN15 1 steel [71], an experimental steel with 0.17% C, 15% Cr, 11% Co, 3.3% Mo, 2.5% Ni, and 2% W [321], or AISI 440C steel [16], both treated via sequence *A*. Repeated CT cycles may further decrease the RA, but only to a very limited extent [17]. On the other hand, pre-tempering prior to cryogenic treatment (sequence *C*) stabilises the retained austenite, which makes subsequent cryogenic treatment less effective in its reduction [70]. Figure 24 provides clear evidence of retained austenite reduction due to the application of CT. Another consequence of CT application is the accelerated precipitation rate of carbides during tempering. This phenomenon was experimentally proven for different steels like those containing 0.15% C, 14% Cr, 13% Co, 2.4% Ni, and 4.8% Mo [69,70], or 0.17% C, 15% Cr, 11% Co, 3.3% Mo, 2.5% Ni, and 2% W [321], or AISI 440C [16]. Also, overall microstructural refinement, see the example in Figure 25, is a typical feature of cryogenically treated martensitic stainless steels. Wang et al. [210] performed thorough investigations of the microstructures obtained through cryogenic treatment (−196 °C/24 h) of AISI 420 steel. In addition to the retained austenite reduction, they recorded a significantly increased amount of additional small globular carbides in the microstructure of the cryogenically treated material, as shown in Figure 26. 

## 4. The Impact of Cryogenic Treatment on the Mechanical Properties and Wear and Corrosion Performance of Steels

The impact of CT parameters and microstructural changes on the mechanical properties, wear and corrosion resistance, and properties important for manufacturing is analysed in the following sub-sections.

### 4.1. Carburised Steels

In carburised steels, Table 1, after final quenching and low-temperature tempering (normally not exceeding 200 °C to prevent a substantial hardness decrease on the surface), the surface layers exhibit microstructural states similar to those of bulk martensitic steels. Cryogenic treatment results in a useful improvement in hardness and wear resistance. 

Cryogenic treatment via sequence *A* increased the hardness of carburised steels without Ni (En353, 1.7131, IS 2062, and 20MnCr5) by 25–100 HV [18,33,46]. The extent of the hardness increase was only marginally affected by CT temperature and duration. However, CT of IS 2062 steel at −77 °C progressively increased microhardness, depending on the duration, by 50 HV0.1 after 3 h of treatment and up to 100 HV0.1 after 24 h of treatment [233]. A lower CT temperature (−196 °C) led to a microhardness increase greater than 300 HV0.1. This result is consistent with the microstructural observation that CT reduces the retained austenite (soft phase) and that the extent of retained austenite reduction increases with the decreasing cryogenic temperature and/or longer treatment duration.

**Figure 27:**
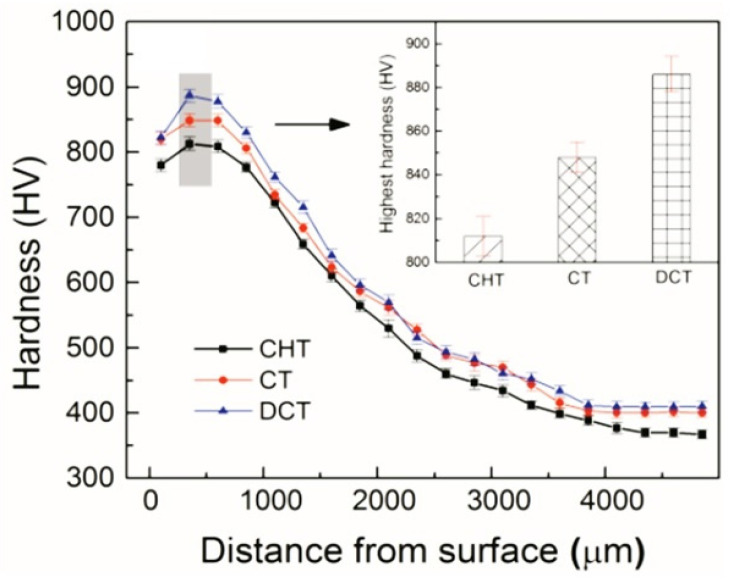
Hardness gradient below the carburised surface of 20CrNi2MoV steel under different heat treatments. CHT—carburised at 935 °C for 27 h, oil quenched from 880 °C, and tempered at 180 °C for 2 h; CT—cryogenic treatment (−80 °C for 4 h) inserted between quenching and tempering; DCT—cryogenic treatment (−196 °C for 4 h) inserted between quenching and tempering. Adapted from [180].

The application of cryogenic treatment (sequence *A*) led to a greater hardness increase in carburised steels in cases when there was a higher retained austenite content present in their microstructures due to supersaturation of the surface. This is particularly relevant for Ni-containing steels. In SNCM 415, a hardness increase of almost 200 HV was observed due to CT at −85 °C for 24 h [225]. For 17Cr2Ni2MoVNb, 21NiCrMo2, and 20Cr2Ni4A steels, there was a hardness increase of almost 40, 80, and 120 HV1, respectively, after CT at −80, −150, or −196 °C (each of them for 1 h) [229,230] or after CT at −120 °C for 2 h [234]. The hardness increase due to cryogenic treatments is a result of a 30 to 50% reduction in retained austenite as well as more extensive precipitation of fine carbides due to cryogenic treatment [229]. For Ni-containing steels, the hardness increase showed clear cryo-temperature dependence for 20CrNi2MoV steel [180] and time dependence for 18NiCrMo5 steel [235]. For example, there was a temperature-dependent hardness increase of 36 and 74 HV for 20CrNi2MoV steel subjected to 4 h of treatment at −80 and −196 °C, respectively (Figure 27) [180]. Furthermore, not only is the hardness increase in the carburised case due to CT evident, but the hardness, in this case, is also higher at a greater depth below the surface, as can be seen from a comparison of the hardness depth profiles in Figure 27.

Tempering prior to cryogenic treatment (sequence *E* or *F*) provides only marginal hardness increments. CT increased hardness by 0.6 HRC for 18NiCrMo5 steel [235], but there was almost no effect for gear wheels made of 18CrNiMo7-6 steel treated at −30, −40, −80, or −196 °C [226]. There were also negligible hardness increments observed for AISI 8620 steel (CT at −40 °C for 1 h) [231] and for 17Cr2Ni2MoVNb and 20Cr2Ni4A steels (CT at −196 °C for 1 h) [230].

The principal explanation for the CT-controlled hardness increase is that cryogenic treatment reduces the retained austenite amount. This reduction is greater for Ni-containing steels [226,229,230] than for steels without Ni [225]. Tempering prior to CT thermally stabilises retained austenite [126,324]. Hence, sequences *E* and *F* act less effectively on RA reduction, and a minimal hardness increment due to CT is a logical consequence. A minor contribution to the overall hardness increase in cryogenically treated carburised cases could be expected from greater precipitation of transient carbides. However, this eventuality requires further clarification through careful and systematic investigations.

Carburised steels are low-carbon and low-alloy steels; hence, their bulk toughness is usually very high. However, carburising results in the formation of a hard high carbon–containing carburised case on their surfaces, a factor that negatively affects their toughness [325]. Carburised 20CrNi2MoV steel (with a 2 mm case depth) was subjected to cryogenic treatment at either −80 or −196 °C for 4 h (following sequence *A*), and there was a 9.8% reduction in Charpy V-notch (CVN) impact energy [232]. For IS 2062 steel carburised to a depth of 0.5 mm, CT at −77 or −196 °C for 3–24 h (sequence *F*) slightly (at −77 °C) or substantially (at −196 °C) worsened CVN impact energy [233]. The toughness deterioration was dependent on the CT duration, that is, the longer the CT, the more remarkable the reduction in toughness. The observed decrease in the toughness of CT steels can generally be attributed to the decrease in RA. Since the RA decrease depends on the CT duration, the decrease in toughness should also be time dependent. Retained austenite is a face-centred cubic (FCC) structure with a high strain hardening exponent [326,327]. Consequently, austenite is a tougher phase and has a higher strain hardening rate. Both the strain hardening rate and toughness of the austenite phase increase with increasing carbon content [328] in the austenite. Therefore, as the austenite volume fraction increases, the fracture toughness (K_IC_) of the material should also increase [329].

**Figure 28:**
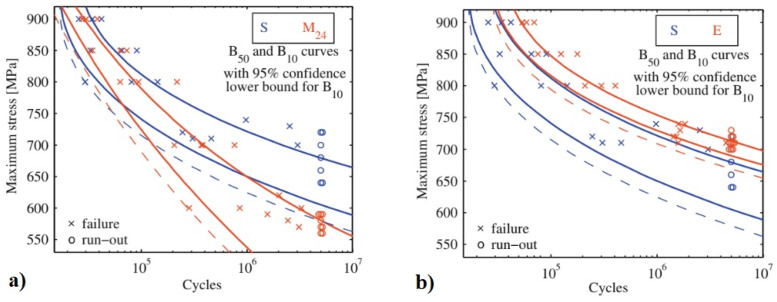
Variations in fatigue performance of cryogenically treated 18NiCrMo5 steel demonstrated upon examples of relevant SN (stress vs. number of cycles) curves: comparison between S—carburised to the carburised case depth of 1 mm, quenched, and tempered at 180 °C for 2 h and M_24_—carburised to the carburised case depth of 1 mm, quenched, cryogenically treated at −185 °C for 24 h, and tempered at 180 °C for 2 h (**a**) comparison between S and E—carburised to the carburised case depth of 1 mm, quenched, tempered at 180 °C for 2 h, and cryogenically treated at −185 °C for 24 h (**b**). Adapted from [236].

Cryogenic treatment of carburised steels leads to variations in their fatigue performance. For 18NiCrMo5 steel, CT at −185 °C for 1 or 24 h, according to sequence *A,* reduced fatigue performance, while sequence *E* improved this property up to 25% [236]. This is illustrated in Figure 28. From the comparison of the SN (stress vs. number of cycles) curve slopes, it is evident that the specimens cryogenically treated by sequence *E* show a fatigue behaviour similar to conventionally treated ones, although transposed horizontally (enhanced fatigue limit) and with an ~82% reduction in scatter. Meanwhile, the specimens treated by using sequence *A* behave in a definitely different way. The fatigue life at higher stress levels appears to be comparable to conventionally treated steel, while the negative impact of cryogenic treatment (sequence *A*) becomes evident at lower stress levels.

Two Ni-containing carburised steels (SAE-4320 and SAE-9310) were treated at −73 or −196 °C following sequence *A* [237]. The bending fatigue endurance limits for the SAE-4320 specimens were 1310 MPa for the carburised condition, 1170 MPa for CT at −73 °C, and 1280 MPa for the −196 °C condition. The endurance limits for the SAE-9310 specimens were 1170 and 1070 MPa for those carburised and cryogenically treated at −73 °C, respectively. To explain the variations in fatigue performance of differently treated carburised steels, it should be noted that the retained fraction of ductile austenite can act as a crack arrestor in the fatigue crack propagation stage. Therefore, retained austenite reduction [18,330] should have a detrimental effect on the final stage of fatigue life. The application of treatment via sequence *A* reduces RA more significantly than sequence *E*; this is logically reflected in the reduced bending fatigue endurance limits of cryogenically treated steels. Furthermore, the whole fatigue process, from nucleation to propagation, is strongly influenced by the presence of residual stresses in the material. These stresses are compressive in a carburised case [331,332], but they are more significantly reduced when tempering is included in the final heat treatment step (sequence *A*) than when sequence *E* is applied. Last but not least, the role of minor but expected precipitation of dispersed nano-carbides [238] should be considered, although this point deserves further careful investigation.

**Figure 29:**
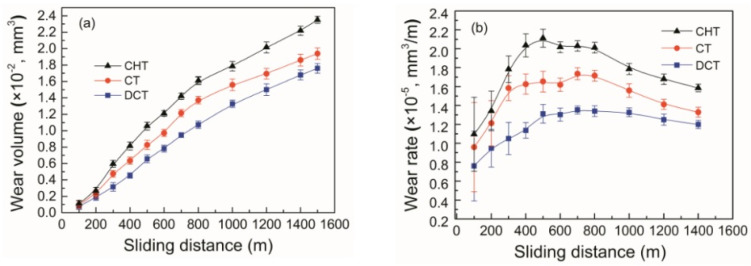
Variations in wear volume (**a**) and wear rate (**b**) with sliding distance of differently treated specimens made of 20CrNi2MoV carburised steel: CHT means conventional heat treatment, CT is cryogenic treatment at −80 °C for 4 h, and DCT is cryogenic treatment at −196 °C for 4 h. Adapted from [180].

Cryogenic treatment following sequence *A* improves the wear resistance of almost all carburised steels. The extent of the improvement depends on both the CT temperature and the duration. The maximum wear resistance for En353, 20CrNi2MoV, 16MnCr5, 20MnCr5, and 17Cr2Ni2MoVNb steels was obtained by treatments at −196 °C (or close to this temperature) [18,33,46,180,229,239], even though the use of higher temperatures (e.g., between −80 and −103 °C) can also significantly improve this property [181,225,228,239]. Two diagrams in Figure 29 clearly delineate an enhanced wear performance of cryogenically treated 20CrNi2MoV carburised steel as compared with the same steel without applying cryogenic treatment. It is also seen that cryogenic treatment at −196 °C (here, denoted as “DCT”) provides the steel with better wear performance than what can be obtained by cryogenic treatment at −80 °C (denoted as “CT”). Treatment at the boiling point of helium (−269 °C) had a positive effect on the wear resistance of 20MnCr5 steel, but this improvement was smaller compared to treatment at −196 °C [33]. Alternatively, CT at −40 °C (following sequence *A* or *F*) had no effect on the wear performance of AISI 8620 steel [231]. As for CT duration, immersion in a cryogenic medium for 24 h resulted in maximum wear performance in most cases [18,33,46,239]. The improvement is commonly attributed to the higher degree of RA-to-martensite transformation (and thus higher hardness) due to cryogenic treatment [18,33,46]. However, the role of the still unclear ‘refinement and better distribution’ of carbides [46,239] should also be considered and investigated systematically.

Concluding remarks: Cryogenic treatments increase the hardness of all carburised steels. Sequence *A* with treatments at or close to −196 °C is more effective than other sequences due to a higher extent of RA-to-martensite transformation. The same applies to the variations in wear resistance. However, an increase in hardness is always accompanied by a decrease in the toughness and fatigue strength of steels. This decrease is small after the application of sequences *E* or *F* but more pronounced when sequence *A* is applied. 

### 4.2. Ball Bearing Steels

For ball bearing steels, shown in Table 2, the retained austenite content and its control are key parameters that determine the final properties and durability of bearing rings and balls. An increase in the retained austenite content contributes to greater fracture resistance of the ring body. In contrast, a decrease in the RA content affects rolling contact fatigue resistance and thus, the total lifetime (durability) of bearings. Rolling contact fatigue is controlled primarily by the volume fraction of carbides and their distribution characteristics and, at the same time, the inherent toughness of the matrix in terms of the carbon content and martensite morphology. 

**Figure 30:**
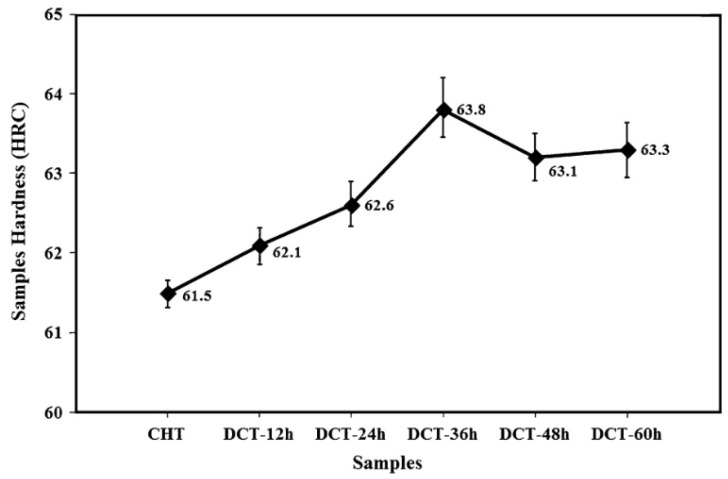
Hardness of conventionally treated (austenitizing at 870 °C, oil quenching, and tempering at 180 °C for 2 h) and cryogenically treated (sequence *E*), at −145 °C for different durations, samples made from AISI 52100 steel. Adapted from [45].

For AISI 52100 steel in the low-temperature tempered condition, cryogenic treatment at −196 °C for 24 and 35 h (sequence *A*) increases the hardness by 60 to 100 HV [24,173]. However, the treatments at higher temperatures can also bring undisputable benefits with respect to the hardness values obtained. Treatment at −100 °C for 210 min (following sequence *A*), for instance, resulted in a 60 HV hardness increase for the given steel grade [228]. Cryogenic treatment at −120 °C for 2 h increased the hardness by 60 to 70 HV [234]. For treatment via sequence *A* applied to near-eutectoid steel (0.86 wt.% C) at −190 °C for either 12 or 36 h, the longer treatment duration resulted in a greater hardness increase [151], mainly due to a more complete RA-to-martensite transformation. On the other hand, repeated CT (cyclic, sequence *C*) was not effective. A maximum hardness value of 64 HRC (CHT resulted in 62.7 HRC) was achieved after the first cycle at −196 °C for 6 h; however, the second and third cycles slightly reduced the hardness [207]. Sequence *E* (with pre-tempering at 180 °C for 2 h before CT at −145 °C for 12–60 h) was less efficient in increasing the hardness of AISI 52100 steel (max. increase of 2.3 HRC at a CT time of 36 h, Figure 30) than sequence *A* [45]. Despite that, the hardness increment is remarkable in this case and can be referred (according to the authors of Ref. [45]) to as the more complete martensitic transformation (even though incomplete due to RA stabilisation by pre-tempering) with the maximum additional SGC count, which occurred at 36 h duration.

**Figure 31:**
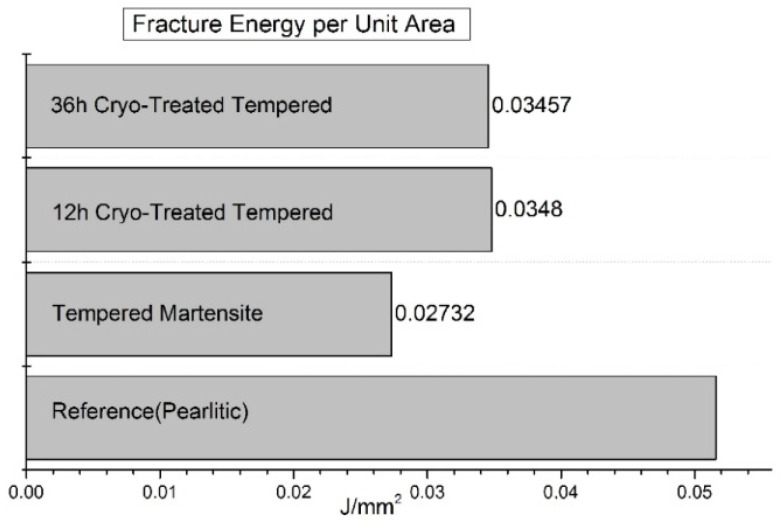
Average impact toughness of differently treated samples made from near-eutectoid steel (0.86% C). Legend: reference (pearlitic) is the initial (as-delivered) microstructure, tempered martensite is the microstructure obtained by austenitising at 860 °C, quenching, and tempering at 200 °C for 2 h, and cryogenic treatment was conducted at −190 °C for either 12 or 36 h (sequence *A*). Adapted from [151].

**Figure 32:**
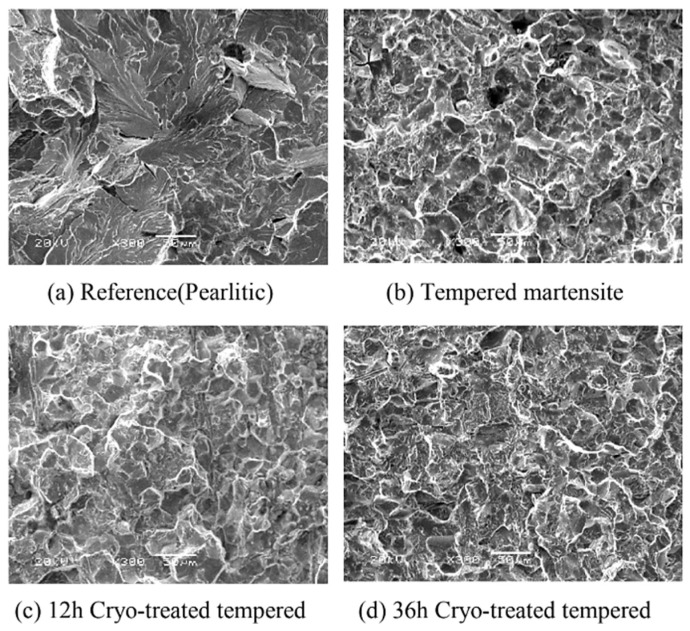
SEM images of fracture surfaces of differently heat-treated samples from Figure 31. Adapted from [151].

Compared to CHT, the CVN impact toughness of near-eutectoid steel (0.86 wt.% C) was improved by almost 27% after CT at −190 °C for 12 or 36 h, as shown in Figure 31 [151]. Changes in CVN impact toughness are reflected in the appearance of fractured surfaces, as shown in Figure 32. The SEM image of the initial (pearlitic) structure shows a ductile–brittle fracture, while for the quenched samples, the fracture model is more likely brittle fracture. Cryo-treated samples have more microcracks, which is a sign of improved toughness. Cryogenic treatment more likely causes a higher amount of additional SGCs, which may act as plastic deformation preventive barrier points. The crack cannot propagate through carbides but only at the carbide/matrix interfaces, which increases the plastic deformation energy until the fracture. One can summarise that cryogenic treatment helps to improve fracture toughness on some level.

In other work [182], Widiantara et al. have reported that retained austenite reduction by half in 0.86% C-containing steel leads to a hardness increase of 2 HRC and to an increase in fracture toughness from 29 MPa·m^1/2^ after CHT to 43 MPa·m^1/2^ after CT (at −40 °C for 24 h, sequence *A*). These results indicate that it is possible to increase the material toughness without sacrificing hardness through CT, owing to overall microstructural refinement (martensitic domains and η-carbide precipitates). Nevertheless, Karaca and Kumruoğlu [234] reported opposite results. The CVN impact toughness of AISI 52100 steel notably decreased with the application of CT at −120 °C for 2 h. This is rather surprising at first glance, but fractographic analyses revealed completely brittle fractures of cryogenically treated samples. One of the basic reasons for this behaviour is the reduction in retained austenite (soft and ductile phase). In various steels, this effect is more or less counterbalanced by the general refinement of the microstructure, the presence of a large number of additional small globular carbides, and the enhanced precipitation of transient carbides. However, these phenomena become active only after a much longer hold at cryo-temperatures, usually 24 h or longer [173,178,180], while the duration of CT in this particular case was only 2 h.

The enhancement of hardness and toughness (when treated at −196 °C) through cryogenic treatment improves wear resistance, a crucial factor for the service life of bearings. For AISI 52100 steel exposed to CT at either −185 °C for 24 h or −195 °C for 36 h (following sequence *A*), a 37–50% (or even slightly more) reduction in wear rate was found [24,244,245]. For steel subjected to CT at −196 °C for 24 h, this reduction was slightly higher (60%) [246]. In other work [228], Karaca et al. reported that an application of −100 °C cryogenic treatment for 3.5 h improved wear performance by only 10%, but the treatment at −120 °C gave a 40% improvement in wear rate [234]. A CT duration of 24–36 h (at −185 or −196 °C) was recommended as optimal to achieve the best wear performance of bearing steels [24,151,244]. Note, however, that the test conditions play an important role in the extent of wear performance. According to Paydar et al. [173], the wear rate of AISI 52100 decreased by about 50% at a sliding speed of 0.05 ms^−1^ but only 25% at a sliding speed of 0.15 ms^−1^. Also, the wear performance of near-eutectoid 80CrMo12 5 steel was improved after CT at −196 °C for 0–168 h or −80 °C for 24 h (sequence *A*) due to a considerable retained austenite reduction and the formation of additional carbides [80]. The maximum improvement in wear behaviour occurred after a 48 h treatment at −196 °C; it coincided with the maximum number of additional carbides and the maximum hardness.

**Figure 33:**
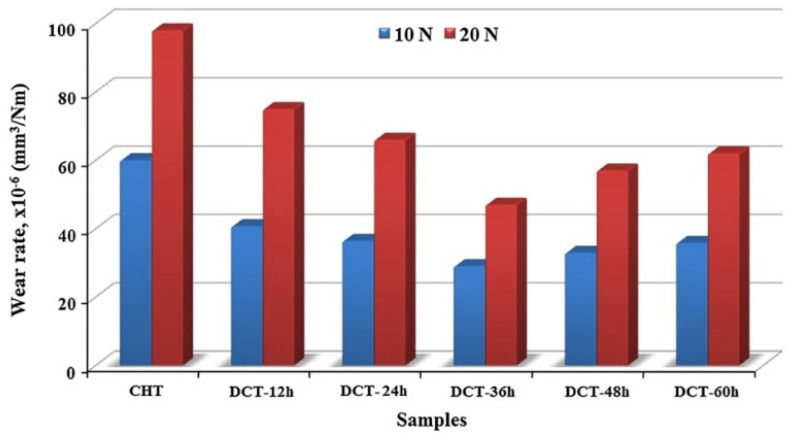
Wear rate of conventionally treated (austenitizing at 870 °C, oil quenching, and tempering at 180 °C for 2 h) and cryogenically treated (sequence *E*), at −145 °C for different durations, samples. The values are recorded and calculated for two different loads, namely, 10 and 20 N. Adapted from [45].

Although sequence *E* is less efficient in increasing the hardness of AISI 52100 steel, there was a relatively large improvement in wear resistance after CT at −145 °C for 12–60 h was applied to pre-tempered steel. The decrease in wear rate was greatest (50%) after 36 h of treatment, shown in Figure 33, and the friction coefficient reached the lowest value for the same treatment duration [45]. 

Corrosion resistance can be of some importance when bearings are operated in harsh environments containing acids, seawater, or other chemical substances. For AISI 52100 steel cryogenically treated at −185 °C for 24 h (following sequence *A*), corrosion resistance was tested in salt spray; cryogenic treatment improved the corrosion resistance by about 50% [24]. This change could be attributed to the transformation of most retained austenite to martensite (an 11% reduction in RA) and the precipitation of fine carbides in the cryogenically treated steels. For the same material cryogenically treated at −196 °C for 24 h, the corrosion resistance was tested in a borate buffer (alkaline environment) using potentiodynamic measurements [247]. The average corrosion rate of the CT samples was 65% lower than that of the CHT samples. The reason for this change was thought to be a lower carbide-to-carbide (interparticle) distance in the cryogenically treated steels. However, rather opposite results were obtained by Wang et al. [210] when tested using a potentiodynamic method in a 3.5% aqueous NaCl solution. Almost no effect on the corrosion resistance was reported in this study for treatment at −196 °C for 24.

Concluding remarks: A hardness increase is an undeniable benefit of using the cryogenic treatments on ball bearing steels. Sequence *A* with treatments at −196 °C for 12–36 h can be recommended to achieve the highest hardness values. The increase in hardness generally results in better wear performance of the cryogenically treated steels, with maximum wear resistance achieved after CT at −196 °C for 24–36 h. This is due to sustained retained austenite, presumably greater additional carbide formation, and enhanced precipitation of nano-sized carbides. Unlike the carburised class of steels, cryogenic treatments may not necessarily reduce toughness, probably due to the general refinement of the microstructure. The effects of cryogenic treatments on corrosion resistance are still unclear. 

### 4.3. Hot Work Tool Steels

For hot work tool steels, shown in Table 3, bulk hardness is a crucial parameter as it controls the wear performance and thermal fatigue. However, the increase in hardness should not be at the expense of toughness. Hot work tool steels are medium-carbon steels. Therefore, they cannot retain a high amount of retained austenite after quenching to room temperature. They are also subjected to high-temperature tempering (570–620 °C) in CHT, which almost completely eliminates retained austenite [252,333]. For treatment of these steels, cryogenic treatment was implemented after performing some (tempering) ‘pre-treatments’ involving heating to temperatures above 500 °C (after CT without tempering (sequence *E*) or followed by high-temperature tempering (sequence *F*)) [19,22,49,51,122,199,260,261,262]. In these cases, there was a minimal effect on the hardness of AISI H13 steel [199,262] or a marginal increase of 1–1.5 HRC for AISI H11 steel treated at −184 °C for 16–24 h [22,121,260,261]. When AISI H13 steel was tempered at a low temperature (100–110 °C) after CT at −185 °C for 8–32 h, there was a greater hardness increase: 5 HRC after 16 h [19] or 2.2 HRC after 32 h [263]. 

**Figure 34:**
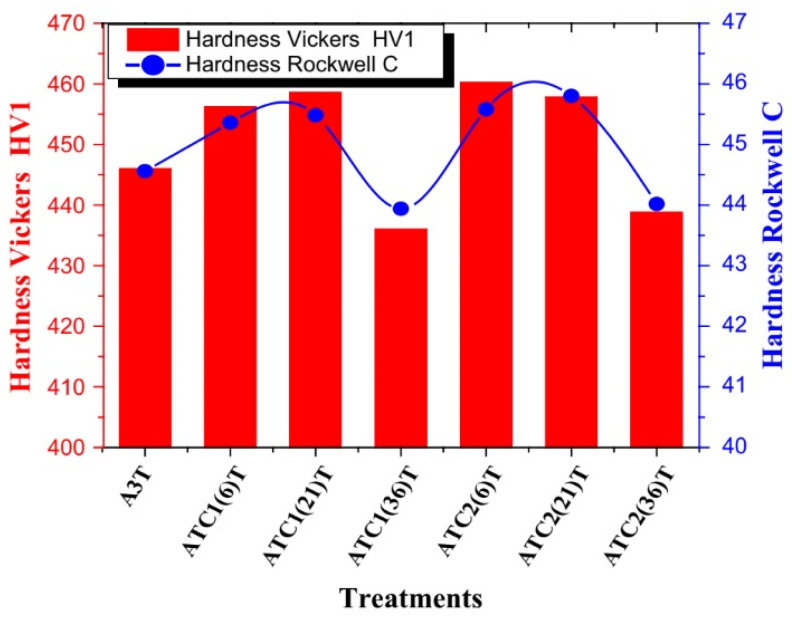
Bulk hardness of conventionally and varied cryogenically treated hot work die steel AISI-H11. Legend: A3T: austenitising at 1040 °C for 30 min, followed by two tempering cycles at 570 °C and 600 °C for 2 h (conventional heat treatment); ATC1(6)T: austenitising at 1040 °C for 30 min, followed by tempering at 550 °C for 2 h, CT at −154 °C for 6 h, and single tempering at 600 °C for 2 h; ATC1(21)T: the same as ATC1(6)T but CT was conducted at −154 °C for 21 h; ATC1(36)T: the same as ATC1(6)T but CT was conducted at −154 °C for 36 h; ATC2(6)T: the same as ATC1(6)T but CT was conducted at −184 °C for 6 h; ATC2(21)T: the same as ATC1(6)T but CT was conducted at −184 °C for 21 h; ATC2(36)T: the same as ATC1(6)T but CT was conducted at −184 °C for 36 h. Adapted from [22].

The diagram in Figure 34 shows changes in hardness of differently cryogenically treated (at −154 or −184 °C for 6, 21, or 36 h) AISI H11 steel following sequence *F* [22]. It is seen that the treatments for 6 or 21 h increase the hardness moderately, while 36 h treatments rather deteriorate this property. Since the retained austenite was stabilised by pre-tempering treatment (it is worth noting that diffraction peaks of this phase appeared in X-ray profiles of each specimen, according to [22]), the hardness variations can be attributed mainly to precipitation of carbides; treatments for 6 or 21 h produce fine and uniformly distributed precipitates, while 36 h treatments give non-uniformly distributed coarser particles.

Tempering must be applied after CT (sequence *A*) to improve hardness more remarkably. For AISI H13 steel treated at −196 °C for 24–35 h, the hardness increase was 3–3.2 HRC [50,125,258,264]. For H11 steel treated at either −80 or −196 °C (each for 24 h), there was a 2 and 4.5 HRC hardness increase, respectively [153]. A special hot stamping steel CR7V was treated at −196 °C for 3–12 h, and after 6 h of treatment, there was a maximum hardness increase of 80 HV [271]. 

Compared to hardness, the response of hot work tool steel to mechanical loading in tension and flexure is more susceptible to the stress–strain state; therefore, cryogenic treatment has a different effect on this property. Cryogenic treatment had almost no effect on the ultimate tensile strength of AISI H13 steel treated in liquid nitrogen for 12 h (following sequence *A*). The yield strength deteriorated slightly (by 40–50 MPa). The ductility was reduced when the material was gas quenched but improved when oil quenched [118,256]. However, when the H13 steel was cryogenically treated after pre-tempering at 560 °C for 2 h (following sequence *F*), the tensile strength was increased as follows: to 1640 and 1720 MPa for CT at −72 and −196 °C, respectively, compared to 1580 MPa for steel subjected to CHT [155]. The use of sequence *F* for the treatment of AISI H11 and H13 steels at either −154 or −184 °C for 6–36 h resulted in a slight deterioration in the ultimate tensile strength (by 5–12%) of the steel alongside significantly improved ductility (up to a 50% improvement) [22,265]. Han et al. [266] examined the tensile properties of selectively laser-printed H13 steel after cryogenic treatment at −196 °C for 24 h followed by 200 °C tempering in one cycle (sequence *A*) and established a 70 MPa enhanced ultimate tensile strength at almost doubled ductility.

The rotating bending fatigue endurance was also investigated for cryogenically treated (at −185 °C for 16 h, following sequence *F*) hot work tool H13 steel [52]. For a 660 MPa stress cycle, the steel completed 263,362 cycles before failure after CHT. After cryogenic treatment, the steel achieved more than 24 million cycles before failure in the high-cycle fatigue regime. There was a similar study for AISI H21 steel after CT at −185 °C for 24 h, according to sequence *A* or *F* [270]. The rotating fatigue limits of AISI H21 steel (at 1 × 10^7^ cycles) were 555, 648, and 740 MPa for the specimens subjected to CHT, sequence *A*, and sequence *F*, respectively. These values indicate a 17–33% improvement in the rotating fatigue limit.

**Figure 35:**
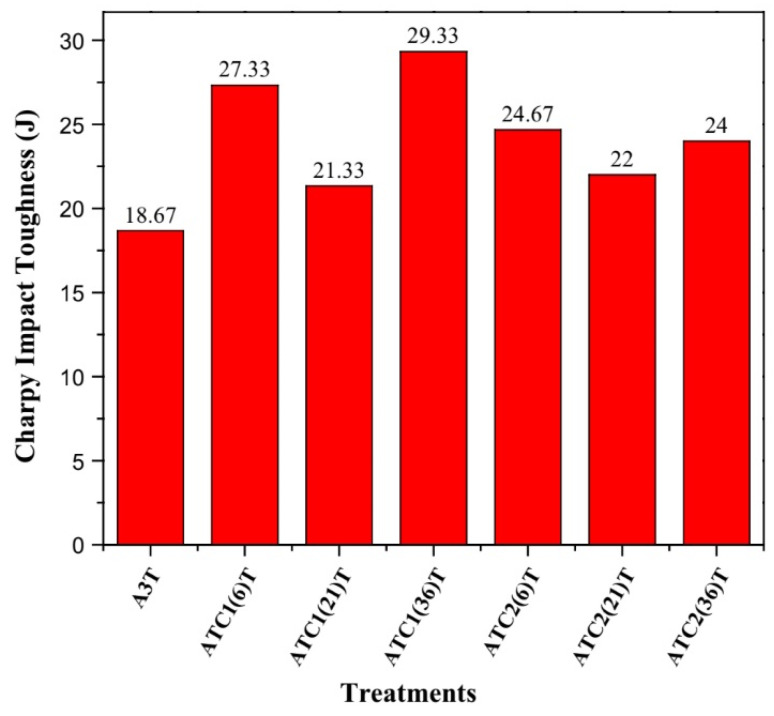
Trend of Charpy impact toughness of conventionally and varied cryogenically treated hot work die steel AISI H11. Legend: A3T: austenitising at 1040 °C for 30 min, followed by two tempering cycles at 570 °C and 600 °C for 2 h (conventional heat treatment); ATC1(6)T: austenitising at 1040 °C for 30 min, followed by tempering at 550 °C for 2 h, CT at −154 °C for 6 h, and single tempering at 600 °C for 2 h; ATC1(21)T: the same as ATC1(6)T but CT was conducted at −154 °C for 21 h; ATC1(36)T: the same as ATC1(6)T but CT was conducted at −154 °C for 36 h; ATC2(6)T: the same as ATC1(6)T but CT was conducted at −184 °C for 6 h; ATC2(21)T: the same as ATC1(6)T but CT was conducted at −184 °C for 21 h; ATC2(36)T: the same as ATC1(6)T but CT was conducted at −184 °C for 36 h. Adapted from [22].

Toughness and fracture toughness parameters reflect the effects of the applied CT sequence in a way similar to strength and ductility. Katoch et al. [22,23,265] treated H11 and H13 steels cryogenically in cold nitrogen gas at −154 or −184 °C for 6–36 h, following sequence *F*. For both steels, CVN impact energy was improved by cryogenic treatment. The improvement was moderate (up to 44%) for CT at −154 °C, while cryogenic treatment at −184 °C tended to slightly increase toughness [22,23,265], as shown in Figure 35. 

In another study [155], it was found that cryogenic treatment at −72 or −196 °C for 8 h, following sequence *F*, slightly improved the CVN impact toughness of H13 steel (by 1–2 J), with increased material hardness (by 6 HRC). It is worth noting that in [22,23,265], the steels were tempered at 600 °C after cryogenic treatment, while in [155], they were tempered at 560 °C after cryogenic treatment. The application of sequence *E* (CT at −185 °C for 35 h) produced only negligible changes in CVN impact toughness, while sequence *A* (with cryogenic temperatures of −185 or −196 °C and durations in the range of 24–35 h) reduced this property [50,125]. Note that sequence *E* led to an increase in hardness (of 1.5–3 HRC) without affecting toughness (a 0.2 J increase). Furthermore, due to the simultaneous effect of quantitatively different strengthening mechanisms, no clear rules can be generalised for cryogenically treated hot work tool steels in terms of CVN impact toughness and fracture toughness. The CVN impact toughness increased by more than 50% for cryogenically treated (at −196 °C for 6 h, sequence *A*) hot stamping CR7V steel, besides a remarkable hardness increase of 80 HV [271]. The result of cryogenically treated AISI H21 steel (at −185 °C for 6–30 h, following sequence *F*) was the opposite [269]. Cryogenic treatment led to a general toughness decrease, with the lowest value recorded after 24-h of treatment. To explain the contrasting results, it should be noted that the pre-tempering before CT left a certain (unspecified) amount of retained austenite in the microstructure [270]. This austenite transformed into martensite during CT, which increased the hardness of the steel. Subsequent “soft tempering” at 100 °C is not sufficient to reduce the brittleness of the newly formed martensite; therefore, reduced toughness could be a logical consequence.

The application of CT generally improves the fracture toughness of hot work tool steels. Using sequence *A* to treat H13 steel improved K_IC_ by either 22–24% (CT at −196 °C for 12 h) [118] or 6% (CT at −185 °C for 35 h) [50]. A direct comparison of the impacts of different sequences (*A* vs *E*) showed that sequence *E* improved K_IC_ more effectively (15%) than sequence *A* (6%) [50]. 

Fractographic observations of CVN impact toughness specimens of H11 or H13 steel [23,265] revealed that CHT materials mainly exhibited cleavage facets and microcracks along the cleavage facets, while cryogenically treated CT specimens (sequence *F*) showed dimples of different sizes and small zones of microvoid coalescence during crack propagation. The mentioned morphology of the fracture surfaces indicates better ductility of CT and post-CT high-temperature tempered steels, which was reflected in generally higher impact toughness. On the other hand, the generally lower CVN impact toughness of the specimens treated following sequence *A* could be related to less retained austenite content as this sequence is more effective in reducing this phase.

**Figure 36:**
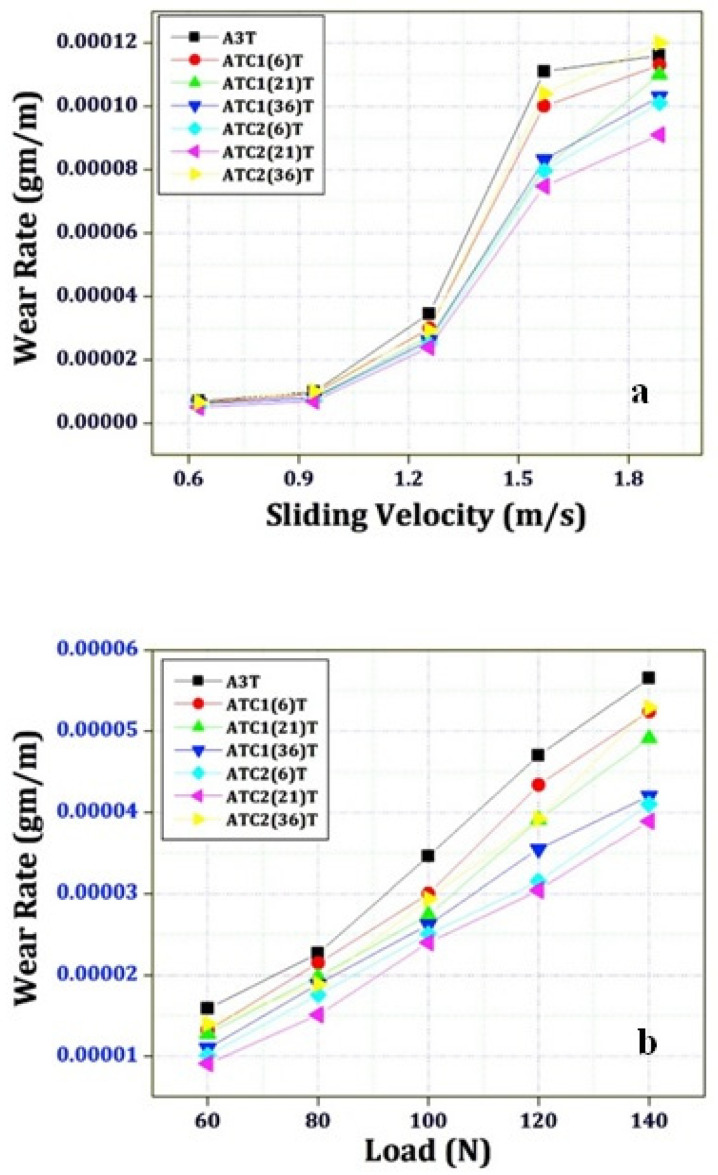
Effect of heat and cryogenic treatment strategies on wear rate: (**a**) wear rate vs. sliding velocity at 100 N load, and (**b**) wear rate vs. load at 1.257 ms^−1^ sliding velocity. Legend: A3T: austenitising at 1040 °C for 30 min, followed by two tempering cycles at 570 °C and 600 °C for 2 h (conventional heat treatment); ATC1(6)T: austenitising at 1040 °C for 30 min, followed by tempering at 550 °C for 2 h, CT at −154 °C for 6 h, and single tempering at 600 °C for 2 h; ATC1(21)T: the same as ATC1(6)T but CT was conducted at −154 °C for 21 h; ATC1(36)T: the same as ATC1(6)T but CT was conducted at −154 °C for 36 h; ATC2(6)T: the same as ATC1(6)T but CT was conducted at −184 °C for 6 h; ATC2(21)T: the same as ATC1(6)T but CT was conducted at −184 °C for 21 h; ATC2(36)T: the same as ATC1(6)T but CT was conducted at −184 °C for 36 h. Adapted from [259].

When examining the wear performance of hot work tool steels, most authors have used heat treatment with tempering before and after CT (sequence *F*). Despite considerable inconsistencies in the test conditions (counterpart nature, load, sliding speed, and distance), some general outcomes can be derived. For AISI H11, H13, and H21 steels, a 16–24 h treatment at −184 °C (or −185 °C) was recommended to achieve the lowest wear rate against steel counterparts [19,49,51,122,199,200,259,260,261,262,269]. However, when hard alumina counterparts were used for wear performance examination, after CT at −180 °C for 32 h, there was only a 12% wear performance improvement in H13 steel [267]. The application of CT at −185 °C for 32 h also had a beneficial effect on the hot wear resistance (testing at 400 °C) of AISI H13 steel [263]. The beneficial effect of cryogenic treatments on wear performance is demonstrated in two diagrams (Figure 36) where the wear rate (WR) is plotted against either sliding velocity (a) or applied load (b) [259]. A general trend of wear rate reduction with the application of cryogenic treatments is clearly shown. The minimum wear rate (maximum wear performance) is obtained by applying a −185 °C cryogenic treatment for 21 h.

The use of treatment according to sequences *A* and *C* (cyclic CT) also leads to better wear performance of hot work tool steels compared to the post-CHT state. Typical examples are the 24% improvement in abrasive wear performance of AISI H13 steel after treatment at −145 °C for 24 h [48], the 30–70% improvements after treatments of the same steel at −196 °C for 18–24 h [257,258,264], about a 35% improvement in the same property of X37CrMoV5 steel after treatment at −160 °C for 12 h [117], the 20–30% improvement in AISI H13 steel after treatments at −80 or −185 °C (for 24 h each) [257], the 62% improvement in hot stamping CR7V steel after treatment at −196 °C for 3–12 h [271], or the 14% wear resistance increase in AISI A8 steel after cyclic CT (five cycles with temperature changing between −172 and −73 °C) followed by two tempering cycles (at 500 °C for 2 h each) [272]. The hot wear resistance of H11 steel improved by 30–40% (at a test temperature of 550 °C) when H11 steel was treated at either −80 or −196 °C (for 24 h) [153]. 

Potentiodynamic corrosion tests of AISI H13 hot work tool steel after CT at −185 °C for 16 h (sequence *F*), on the other hand, indicated that this treatment did not benefit the corrosion resistance of the given steel [268].

To verify changes in mechanical properties, it should first be noted that all measurements were conducted after the steels had been high-temperature tempered, and the only difference between the processing methods used was whether the tempering occurred before CT (sequence *E* or *F*) or after CT (sequence *A*).

Based on the microstructural changes (described in Section 3), the hardness variations in hot work tool steels are mainly related to alterations in the amount of retained austenite. Sequence *A*, performed at or near −196 °C, reduces RA most effectively. The role of a possible increase in the number and population density of carbides as well as changes in the precipitation kinetics of nano-sized carbides is still unclear and deserves further careful investigation. One can only speculate whether applying sequence *F*, additional tempering (after CT), could induce a coarsening of precipitates already present (after pre-tempering before CT), which could have a slightly detrimental effect on hardness. On the other hand, no conclusive statement can be expressed with respect to the effect of CTs on the tensile properties of hot work tool steels. 

Variations in wear rate due to the application of cryogenic treatment could be attributed to the combined effects of greater RA-to-martensite transformation (although the extent of this can only be roughly estimated due to the ‘distortion’ caused by high-temperature tempering used in treatment sequences), more pronounced carbide precipitation, and martensite refinement [334]. As harder and finer martensite forms, along with a higher number and population density of nano-sized precipitates, the wear resistance of hot work tool steels is generally improved through CT. There have been opposite effects regarding corrosion resistance: the presence of carbide precipitates has a detrimental effect on corrosion resistance as microelectrochemical cells are formed at the carbide/matrix interfaces. This has been confirmed by many authors for ledeburitic steels containing lamellar eutectic mixtures [335] or high-Cr white cast irons [336,337,338,339,340]. Nano-sized precipitates in hot work tool steels are formed by diffusion processes at elevated temperatures. They differ chemically from the matrix and therefore form microcells at their interfaces with the matrix.

Concluding remarks: Most of the experimental work has been carried out using the sequences *E* or *F*, and only a small proportion of experiments (especially the most recent) have used sequence *A* for the CT of hot work tool steels. From a detailed review, it appears that the use of sequence *A* results in better hardness than the other sequences. The reason for this could be that in the quenched condition, more retained austenite is available for transformation during CT than in sequences with prior-to-CT tempering. Recommended parameters for CT are temperatures from −185 to −196 °C and a duration of 24–35 h. The changes in hardness are closely related to alterations in wear performance. If the aim is to enhance the wear resistance, then similar parameters of CT should be used. The results obtained for tensile strength show clear inconsistencies. Further systematic research on the effect of CT on this property is needed before drawing a decisive conclusion. It seems that CT has a beneficial effect on the fatigue resistance of hot work tool steels. This could be due to the increased hardness that results from transforming retained austenite into martensite. A higher hardness would certainly delay the initiation of cracks, leading to an increased number of cycles before failure. On the other hand, the results indicate a deterioration in the corrosion resistance after CT due to the increased precipitation of nano-sized carbides during the subsequent high-temperature tempering.

### 4.4. Ledeburitic Steels and Eutectic Iron Alloys

**Figure 37:**
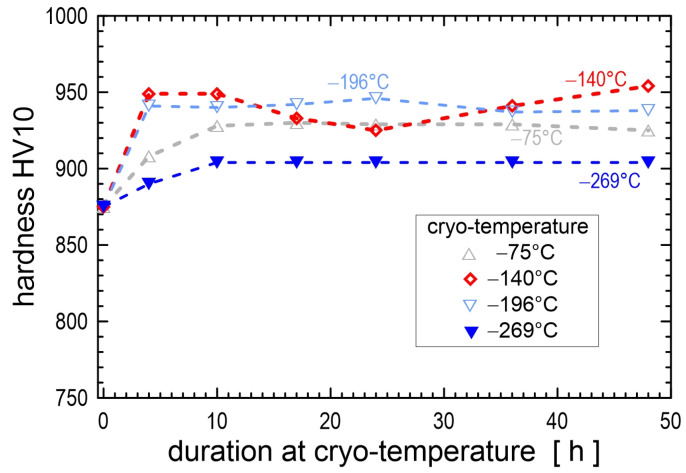
Hardness HV10 dependence on the cryogenic treatment duration considering four CT temperatures applied for the treatments of Vanadis 6 steel. The hardness values were taken from published studies [15,21,35,59,116,179].

There are significant differences in the hardness of ledeburitic steels exposed to cryogenic treatment or conventional heat treatment, as shown in Table 4. This is mainly observed in steel microstructures before tempering. For Cr-ledeburitic steels such as AISI D2, X290Cr12, and X210CrW12, an increase in hardness of 2–3 HRC was observed after CT in liquid nitrogen [32,78] or at −120 °C [157]. Moreover, the extent of hardness increase was practically the same for short (15 min) or long (24 h) treatments. For white cast iron, there was a 4–9 HRC hardness increase (CT at −196 °C) [307]. Vanadis 6 steel subjected to CHT had a hardness of 875 HV, but samples subjected to CT had a hardness of 920–950 HV. Treatment at −75, −140, or −196 °C for 17–24 h resulted in the greatest hardness [15,30,57,58,59,60]. The variations in hardness of cryogenically treated Vanadis 6 steel in an as-quenched state (before tempering) are summarised in Figure 37. The curves show that the CT temperature affects the hardness level more markedly than the duration at CT temperature after a duration of about 5 h. Finally, the application of austenitising temperatures higher than those recommended by steel manufacturers before CT (e.g., 1100 or 1200 °C for Cr-ledeburitic steels) resulted in a much more pronounced hardness increase (by 15–20 HRC) [32].

The higher hardness of cryogenically treated steels in the prior-to-tempered state could be simply related to a more complete RA-to-martensite transformation, additional small globular carbides, and accelerated precipitation of transient carbides. Both the extent of the RA-to-martensite transformation and the amount of additional SGCs were the greatest for the CT duration of 17–24 h. Therefore, the hardness of intrinsically non-homogeneous steels, such as ledeburitic steels, is maximised by using a treatment duration that maximises the above microstructural changes.

The hardness variations due to tempering of cryogenically treated ledeburitic steels (and also high-speed steels, see Section 4.5) are quite complex [59]. According to the modified Kulmburg’s consideration [341], the final tempering curve consists of four components: (a) martensite tempering, which reduces hardness. However, cryogenically treated steels contain more martensite, which can retard the decrease in overall hardness due to tempering. Therefore, low-temperature tempered CT steels have higher hardness than CHT steels. (b) Contribution of secondary retained austenite transformation to martensite. This leads to a significant increase in hardness during high-temperature tempering; however, CT steels contain considerably less retained austenite. Therefore, the contribution of the RA-to-martensite transformation to secondary hardening is small [202]. (c) The presence of additional small globular carbides in cryogenically treated steels. These carbides have a positive effect on hardness, but their number decreases moderately with increasing tempering temperature [15,21,116]. Therefore, the effect of these particles is more significant at lower tempering temperatures, while it is suppressed after high-temperature tempering. (d) Precipitation of nano-sized carbides. At low tempering temperatures, the precipitation of transient carbides is accelerated [21,116,179,194,201,202,203], and the contribution of these particles to the final hardness is positive. On the other hand, precipitation of stable carbides (at higher tempering temperatures) is suppressed due to CT [21,206]; therefore, the positive contribution of carbide precipitation at high temperatures is expected to be lower. More additional SGCs and martensite (in most cases) cannot fully compensate for the retained austenite reduction due to less intense precipitation of carbide nano-particles. Lower hardness (and loss of the secondary hardening) of cryogenically treated and high-temperature tempered ledeburitic steels is therefore logical.

Sequence *A* has mostly been used to examine the impact of cryogenic treatment on the hardness of tempered ledeburitic steels, and this sequence increases hardness most effectively. For conditions after low-temperature tempering (180–210 °C), cryogenic treatment improved hardness compared to conventional heat treatment. The extent of improvement depended on the CT temperature. The maximum increase for D-class steels occurred after CT at −196 °C for 36–60 h. Such treatment improved the hardness by 30–55 HV compared to CHT [12,14,25,26,29,53,54,55,78,158,160,162,212,278,290]. Considering AISI D2 steel after CT (at −185 °C for 36 h)—as well as the effect of the number of tempering cycles (at 210 °C for 2 h each, sequence *A*)—the hardness after the first, second, and third tempering cycles increased by 3, 1.8, and 0.8 HRC, respectively, compared to CHT [282]. Higher hardness after CT and low-temperature tempering, compared to CHT, was also reported for other ledeburitic and sub-ledeburitic steels that were also treated at a temperature above −196 °C: Vanadis 6 steel after CT at −75, −140, −196, or −269 °C (tempering in the range 170–450 °C) [15,58,59,298], sub-ledeburitic DC53 tool steel (tempered at 210 °C) [203], or Sleipner steel after CT at either −80 or −180 °C [248,306]. On the other hand, the use of sequence *E* or *F* resulted in almost no hardness improvement in D-class tool steels [283].

For the states after high-temperature tempering (or tempering for secondary hardening), the hardness values of cryogenically treated ledeburitic steels were mostly lower than those conventionally quenched to room temperature. Moreover, the steels lost the secondary hardness peak, as demonstrated for AISI D2 and 190CrVMo20-4 steels treated at −120, −160, or −196 °C for ≥5 h [156,211,284,288,289], HVAS steel [304], or Vanadis 6 steel after CT at −75, −140, −196, or −269 °C [57,58,59,60,299]. At a shorter CT duration, the secondary hardness peak did not disappear completely, but it shifted (by 20–30 °C) to a value lower than normally used (520–530 °C) tempering temperatures, as shown by the examples of three Cr-ledeburitic tool steels (X210CrW12, X165CrMoV12, and X155CrVMo12-1) [32]. In one of the most recent works, Mochtar et al. [285] treated AISI D2 steel at −196 °C for 5 min, and they arrived at a very similar finding. A small exception to the general trends mentioned is Vanadis 8 steel: hardness increased by 0.4 HRC after tempering cryogenically treated steel at 560 °C [303]. Finally, CT of Vanadis 6 steel at −140 °C for 17 h resulted in the loss of the secondary hardness peak, but the hardness in the tempered state (at 530 °C) was higher than that after CHT [59]. 

Vanadis 6 steel subjected to cryogenic treatments (at −75, −140, or −269 °C, following sequence *A*) showed slightly higher flexural strength compared to CHT, regardless of the tempering temperature (170–600 °C) [15,58,59,60,298]. Conversely, CT at −196 °C for 17 or 24 h reduced the flexural strength [28,299], while a shorter treatment (up to 10 h) produced better values of this material property [60]. For AISI D2 and 190CrVMo20-4 steels, CT followed by high-temperature tempering (following sequence *A*) had almost no effect on the flexural strength [288].

A drastic reduction in the CVN impact toughness of D-class ledeburitic steels, as well as of sub-ledeburitic tool steels following the application of CT followed by low-temperature tempering (sequence *A*), was demonstrated in many works [279,286,294,305]. The application of sequences *E* or *F* also had a detrimental effect on CVN impact toughness as reported by Li et al. for DC53 steel [305]. The extent of the toughness decrease depends on the CT temperature. For example, the minimum toughness of AISI D2 steel occurred when treated at −70 °C, followed by a moderate increase in toughness when a lower cryogenic temperature was used [81]. Furthermore, the reduction in toughness is more pronounced for longer CT durations. The only way to improve CVN toughness through CT, compared to CHT steels, is to temper the steels to the secondary hardness peak.

**Figure 38:**
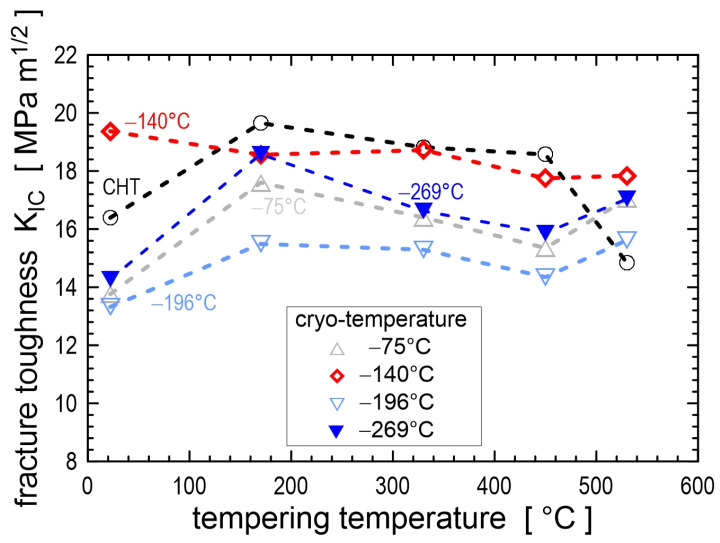
The effect of cryogenic treatment and tempering temperatures on fracture toughness of Vanadis 6 steel. The values used in this diagram are from published studies as follows: CHT—conventional heat treatment [15,59,298], −75 °C [15], −140 °C [59], −196 °C [30,58], and −269 °C [298].

Trends in CVN toughness associated with cryogenic treatment are closely related to variations in fracture toughness [342]. For AISI D2 steel, CT at 75, 125, or 196 °C (following sequence *A*, low-temperature tempering at 210 °C) resulted in a decrease in K_IC_ of 3.6, 7.7, and 2.5 MPa·m^1/2^, respectively, compared to CHT [278]. For CT at 196 °C for 4 h followed by tempering at 480 °C, the fracture toughness of the same steel grade was lower (22.7 MPa·m^1/2^) than for CHT (25.4 MPa·m^1/2^), a difference of about 8% [280]. Cryogenic treatment CT also reduced the fracture toughness of the prior-to-tempered Vanadis 6 steel compared to the post-CHT state; this trend was maintained after tempering at 170, 330, or 450 °C [15,28,58,59,298]. Figure 38 represents the fracture toughness values in the prior-to-tempered state and as a function of tempering temperature; only the state after CT at 140 °C shows K_IC_ values close to the conventional treatment. However, when tempered at 530 °C (secondary hardening peak), the cryogenically treated steel exhibited better fracture toughness than the steel after conventional quenching [58,59,299]. Also, another general trend can be derived. While cryogenic treatments at 75, 196, or 269 °C combined with low-temperature tempering significantly reduced fracture toughness, there was only a slight decrease in fracture toughness after CT at 140 °C (Figure 38). 

The main explanation for the very low toughness and fracture toughness is that CT and low-temperature tempered steels contain less retained austenite and, accordingly, a higher portion of hard and brittle martensite [329]. In addition, the martensite contains more nano-sized precipitates, which may tend to reduce its plasticity. More additional small globular carbides, which can essentially act as barriers to crack propagation, cannot fully compensate for the above microstructural phenomena. The possible increase in the CVN impact toughness of CT and high-temperature tempered steels could be due to the fact that high-temperature tempering leads to significant martensite softening and thus, lower hardness. Furthermore, cryogenic treatment suppresses the precipitation of stable carbides at high tempering temperatures, thus enhancing the plasticity of the matrix compared to the same steel after CHT.

Thanks to specific microstructural changes, cryogenic treatment provides ledeburitic steels with the possibility of simultaneous increasing hardness (strength) and toughness, albeit only to a very limited extent and within a very narrow processing window. Ghasemi-Nanesa [281] was the first to point out a simultaneous increase in the above mechanical properties, which are often in strong contradiction, in cryogenically treated AISI D2 steel. A very similar finding resulted from experimental works on cryogenically treated Vanadis 6 steel, as Figure 37 illustrates. 

Sequence *A* with low-temperature tempering as the post-CT treatment has mostly been used to investigate the abrasive or adhesive wear resistance of cryogenically treated ledeburitic cold work tool steels. For AISI D2 steel, maximum abrasive wear resistance was achieved by a 36 h treatment in liquid nitrogen (followed by tempering at 210 °C for 2 h) [27,29,54,55]. A longer dwell time in liquid nitrogen did not provide additional benefits [196]. Furthermore, CT at higher temperatures (e.g., 75 or 125 °C) led to less pronounced improvements in wear resistance. Improvements in wear resistance can be attributed to both the retained austenite reduction and the presence of more and a larger volume fraction of additional SGCs [56,343,344,345]. Moreover, variations in wear performance are associated with a change in the wear mechanism. At a low load, up to approximately 30 N, the wear resistance improvement was only 1.7 times, whereas the wear mechanism was identified as oxidative for both the CHT and CT steel specimens [27]. However, at a higher load, up to 69 N, the wear mechanism for the CHT specimens (and also for the specimens exposed to CT at 75 °C) changed early to a delamination mechanism, resulting in a significant difference in wear performance between the CHT and CT samples, up to 82-fold. A further increase in the applied load led to a transition of the wear mechanism from light to heavy for the cryogenically treated specimens, which reduced the improvement in wear performance to 2–3.3 [26,27]. In the high load range, with a load of about 100 N, the average improvement was further reduced to about 85%, while it slightly decreased with increasing contact load [55]. 

**Figure 39:**
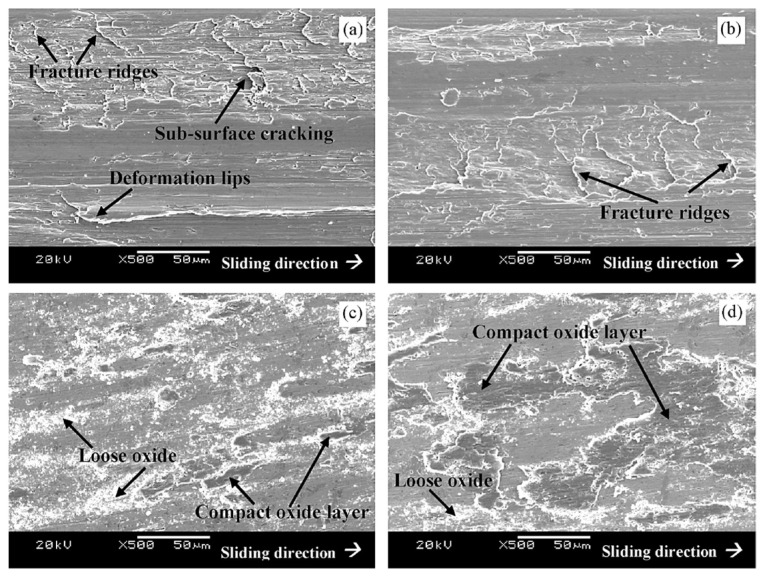
Scanning electron micrographs of worn surfaces at the end of wear tests of (**a**) conventionally heat treated, (**b**) cryogenically treated at −75 °C, (**c**) cryogenically treated at −125 °C, and (**d**) cryogenically treated at −196 °C AISI D2 steel specimens tested at a sliding velocity of 1.25 ms^−1^. Adapted from [54].

**Figure 40:**
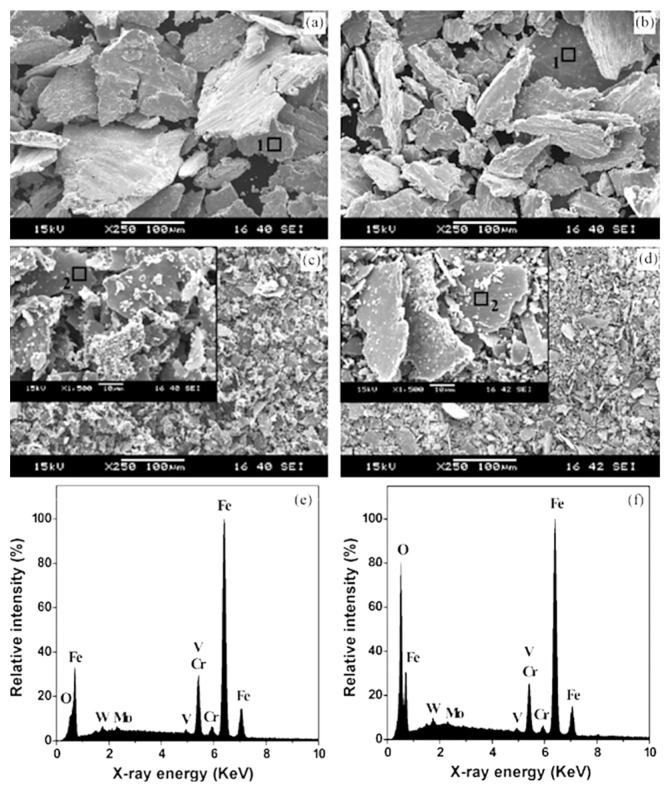
Scanning electron micrographs of wear debris generated corresponding to the steady-state wear regime of (**a**) conventionally heat treated, (**b**) cryogenically treated at −75 °C, (**c**) cryogenically treated at −125 °C, and (**d**) cryogenically treated at −196 °C AISI D2 steel specimens tested at a sliding velocity of 1.25 ms^−1^. All micrographs are at the same magnification of 250×, whereas the insets in (**c**,**d**) show details of the same micrographs (1500× magnification) and (**e**,**f**) energy-dispersive X-ray spectroscopy profiles taken from the rubbed surfaces of the wear debris as marked by location 1 in (**a**,**b**) and by location 2 in the inset of (**c**,**d**), respectively. Adapted from [54].

**Figure 41:**
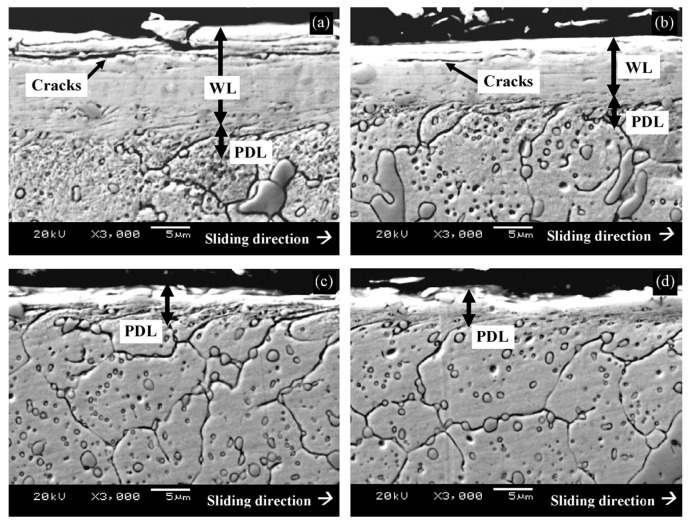
Backscattered scanning electron sub-surface micrographs of worn surfaces at the end of wear tests of (**a**) conventionally heat treated, (**b**) cryogenically treated at −75 °C, (**c**) cryogenically treated at −125 °C, and (**d**) cryogenically treated at −196 °C AISI D2 steel specimens tested at a sliding velocity of 1.25 ms^−1^. WL—white layer; PDL—plastically deformed layer. Note that average hardness values were 759, 778, 787, and 791 HV 60 for conventionally treated, −75 °C-treated, −125 °C-treated, and −196 °C-treated specimens. But the mean population densities of additional SGCs were 160 × 10^3^, 293 × 10^3^, 345 × 10^3^, and 485 × 10^3^ mm^−2^ for the specimens treated conventionally, at −75, −125, and −196 °C, respectively. Adapted from [54].

Figure 39 shows representative features on the worn surfaces of specimens subjected to CHT or CT at −75, −125, or −196 °C. The worn surface of the CHT steel (Figure 39a) appears relatively rough and exhibits fracture ridges and deformation lips stretched parallel to the sliding direction. The presence of deformation lips suggests that the CHT specimen has undergone heavy plastic deformation during the wear test, accompanied by delamination of the deformed material. Similar features are also typical for specimens subjected to CT at −75 °C (Figure 39b). In contrast, the worn surfaces of the other two specimens (Figure 39c,d) are much smoother and manifest the presence of more or less compact oxides. The changes in the wear mechanisms are closely related to the morphology and composition of the produced wear debris. While the wear debris of CHT specimens and specimens subjected to CT at −75 °C is almost fully metallic (Figure 40a,b,e), the wear debris of specimens subjected to CT at −125 or −196 °C is covered by oxides (Figure 40c,d,f). In addition, the near-surface region of specimens subjected to CHT or CT at −75 °C exhibit heavy plastic deformation accompanied by cracking and delamination (Figure 41a,b). On the other hand, the plastic deformation in the near-surface regions of specimens subjected to CT at −125 or −196 °C is much lower (Figure 41c,d), suggesting oxidative wear of the steels treated in this way.

Other cryogenically treated (at about −196 °C) and low-temperature tempered D-class ledeburitic tool steels such as AISI D6 [13,78,158,346], AISI D5 [296], or AISI D3 [159,161,292,293] were also found to show a significant improvement in wear performance (up to 68–80%). The optimum dwell time was found to be 24–48 h. Examination of the effect of the number of tempering cycles at 150 °C on the wear resistance of AISI D3 steel revealed a 93% improvement after the first tempering cycle; additional tempering cycles reduced the extent of wear resistance improvement. Investigation of the effect of treatment sequences (*A* vs. *F*) on the wear performance of AISI D2 steel (CT at −185 °C for 36 h) showed that sequence *A* provides better wear performance than sequence *F* [282]. Alternatively, the application of cryogenic treatments at either −80 or −180 °C, both for 12–36 h and followed by 200 °C tempering (sequence *A*), did not provide the Sleipner sub-ledeburitic tool steel with any benefit with respect to the abrasive wear resistance [306].

When high-temperature tempering was applied in treatment schedules, cryogenic treatment resulted in significantly less abrasive wear resistance improvement (up to 30%) for AISI D2 grade steel [287,304]. Marginal or no effects from CT followed by high-temperature tempering were also found if abrasive wear occurred (when hard counterparts such as alumina are used) in the cases of 190CrVMo20-4 and Vanadis 6 steels [283,300]. Alternatively, standardised (according to [347]) pin-on-disc tests of cryogenically treated Vanadis 6 steel (at −90 °C for 4 h, −196 °C for 4 h, or −196 °C for 10 h) tempered at 530 °C showed better resistance to adhesive wear against 100Cr6 steel or bronze counterparts. In two recent papers, Yarasu et al. [301,302] studied mixed abrasive–adhesive (against 100Cr6 ball) and anti-galling (against CuSn6 bronze) properties of different cryogenic treatments (−75, −140, and −196 °C) of Vanadis 6 steel followed by low- or high-temperature tempering. They recommended using −196 °C cryogenic treatment followed by 530 °C to maximise the galling resistance of the examined tool steel. In contrast, cryogenic treatment at −140 °C with 170 °C tempering provided the steel with the best abrasive/adhesive wear resistance.

**Figure 42:**
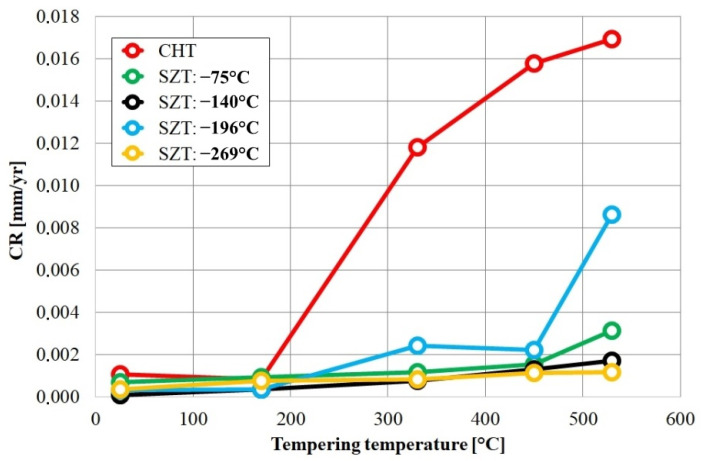
Corrosion rate (CR) dependence on the tempering temperature for conventionally heat-treated specimens made of Vanadis 6 steel and for specimens made from the same steel after application of different cryogenic treatment temperatures (the abbreviation SZT means sub-zero treatment in the original source). Adapted from [36].

For ledeburitic steel tools operating in harsh corrosive environments, corrosion resistance is a key parameter that determines their durability. Carbides are known to exhibit much more noble behaviour in a variety of corrosive environments and can effectively protect metallic surfaces from corrosion [348]. Cryogenically treated ledeburitic steels contain more carbides; therefore, a larger area fraction of their exposed surfaces is covered with phases that are comparatively more noble than ferrite or austenite. The corrosion resistance of ledeburitic steel could thus be improved by CT. This assumption was confirmed for X190CrVMo 20-4 steel subjected to CT (−196 °C for 15 min, sequence *A*, and tempering at 200 or 540 °C) and tested in a 0.5 M sulphuric acid solution [297]. Cryogenic treatment also improved the corrosion resistance of Vanadis 6 steel (in a 3.5% aqueous NaCl solution). When the steel was low-temperature tempered after CT, the improvement was most pronounced after treatment at −140 °C. On the other hand, CT in liquid helium gave the best corrosion resistance for high-temperature tempered steel (Figure 42) [36]. By contrast, the corrosion resistance of 1.2080 steel grade (AISI D3) in 3.5% NaCl solution was worse after CT in liquid nitrogen for 24–48 h [291,295]. This phenomenon was attributed to an increased carbide content [291], which reduces the number of dissolved Cr atoms in martensite and increases the number of martensite/carbide interfaces (galvanic cell areas). However, an opposite result has also been reported: a >50% improvement in the corrosion resistance of AISI D3 steel in borate buffer (pH 10) [247].

Based on published data, there is disagreement about the change in corrosion resistance in response to CT. So far, there is no general explanation for this phenomenon. It appears that only steels produced by powder metallurgy (X190CrVMo 20-4 or Vanadis 6) show improved corrosion resistance after CT. The effect of CT on the corrosion resistance of wrought steels (such as AISI D3) remains unclear.

Concluding remarks: Cryogenically treated and low-temperature tempered ledeburitic steels invariably exhibit a great increase in hardness due to a much more complete transformation of austenite to martensite, increased precipitation of transient carbides, and the formation of a large number of additional small globular carbides. To maximise hardness, treatment in the range from −140 to −196 °C for 17–36 h is the best choice. In this case, however, deterioration of toughness is inevitable; it is more pronounced in cast and wrought steels (D-class), while acceptable toughness can be achieved in newly developed PM grades. High-temperature tempering of cryogenically treated steel often results in the loss of the secondary hardness peak and thus in somewhat lower hardness than CHT steels. In this case, improved toughness is one of the advantages of this type of treatment. Wear resistance can be extremely improved by cryogenic treatments (from −140 to −196 °C for 17–60 h, depending on the steel grade) followed by low-temperature tempering. Cryogenically treated and high-temperature tempered steels also have better wear resistance than conventionally treated steels, but the extent of wear resistance improvement is relatively small. It appears that corrosion resistance can be improved through CT of PM grades with fine and more uniformly distributed carbides. On the other hand, there are unclear results when cast and wrought grades have been cryogenically treated. 

### 4.5. High-Speed Steels

High-speed steels, shown in Table 5, like ledeburitic cold work tool steels, are high-alloy and intrinsically non-homogeneous steels. They also contain martensite, retained austenite, and carbides in their as-quenched microstructures. It can be assumed that the application of cryogenic treatment to high-speed steels has effects similar to ledeburitic tool steels. High-speed steels are usually tempered at >500 °C to achieve so-called secondary hardness [349]. Therefore, unless otherwise stated, the experimental results described below refer to high-temperature tempering after CT (sequence *A*). 

**Figure 43:**
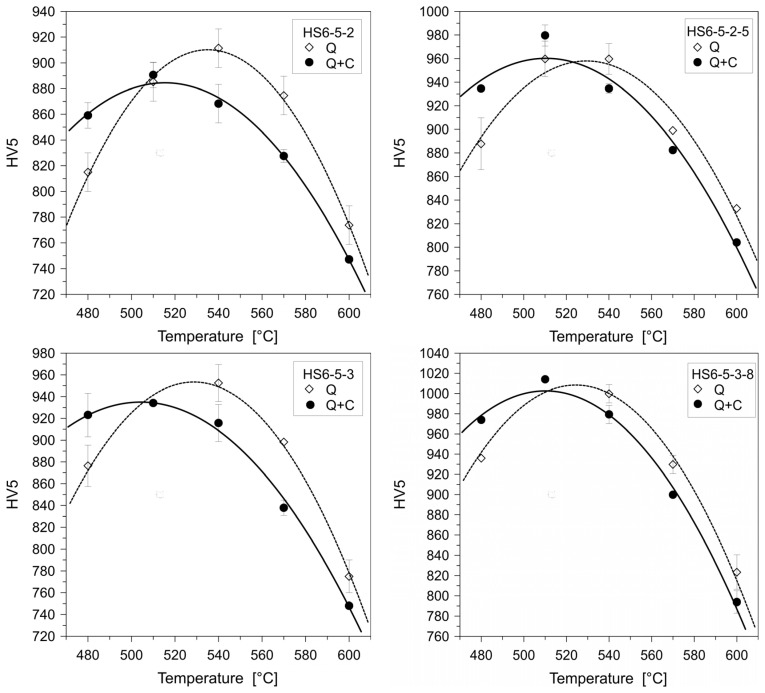
The influence of cryogenic treatment at −180 °C for 24 h on the tempering curves of four different high-speed steels, namely, HS 6-5-2 (AISI M2), HS 6-5-2-5 (AISI M35), HS 6-5-3 (AISI M3:2), and HS 6-5-3-8 grades. Adapted from [121]. Legend: Q—as-quenched, Q + C—as-quenched + cryogenically treated.

Tempering high-speed steels after cryogenic treatment (at temperatures close to −196 °C) reduced the maximum secondary hardness peak temperature by 15–30 °C [121] in a way similar to chromium ledeburitic steels, as reported by Berns [32]. The diagrams in Figure 43 show the extent of the shift of the maximum secondary hardness peak temperature to lower tempering temperatures as well as the fact that the maximum achievable hardness can be lower after cryogenic treatments for some high-speed steels grades [121]. However, opinions on the effect of post-CT tempering on the resulting hardness are different and often contradict one another, although obtained by examination of the same steel grade. A reduction in the maximum achievable hardness was experimentally demonstrated for AISI M2 or HS 6-5-3 (AISI M3:2) in the works [63,168]. No effect of CT at −196 °C on the hardness of AISI M35 and experimental (low-alloyed HSS with 2.8% Mo, 2.55% W, 2.1% V, and 4.5% Co) steels in the tempered condition was found in the works [63,164,168]. However, most of the experimental works led to hardness increments in various high-speed steels due to cryogenic treatment. This concerns the AISI M2 [50,62,64,65,115,350], AISI W9 [10], AISI M35 [170,315], HS6-5-3-8 [63,168], and S390 Microclean grades [166,167]. The extent of this hardness increase seems to depend on the CT temperature. For AISI M2 and AISI W9 steels, hardness increased as the CT temperature decreased from −80 to −196 °C [10,65]. The cryogenic treatment duration also affected the hardness, with a maximum value at 24 h of treatment (+3.8 HRC compared to CHT) for AISI M2 steel [65]. Xu et al. [186] pointed out that the hardness of AISI M35 steel increases up to 5 h of CT at −196 °C, and further duration of CT does not have a practical effect on hardness. There are also convincing experimental results demonstrating that sequence *A* has a greater effect on increasing hardness than sequence *E* or *F* for AISI W9 [10], M2, M35, or T1 steels [62,64,170,350]. The effectiveness of sequence *C* should not be neglected. This sequence increased hardness the most for both AISI M2 and T1 steels [64,350]. Interesting results were achieved through CT (at −196 °C for 16 or 24 h) of AISI M2 steels produced by different methods. While the treatments of cast steel had no effect on the steel hardness, this property was increased by 27 and 40 HV, respectively, after 16 and 24 h treatments of PM steel [316].

The effect of prior-to-CT treatment (especially the austenitisation temperature) was also examined. For AISI M2, M3:2, and M35 steels, the hardness increased due to CT when austenitising temperatures were lower than those recommended by the steel suppliers, but hardness tended to decrease when the steels were austenitised at higher temperatures [67,317]. PM steel S390 Microclean was treated at −196 °C for either 25 or 40 h according to sequence *A* [166,167]. CT after austenitising at 1130 °C did not change the hardness, but austenitising at 1230 °C increased the hardness by 1.5–2 HRC. 

Tempering at low temperatures as a final treatment step also increases hardness after cryogenic treatment. For AISI M2 steel, the hardness increased from 62.2 HRC (after CHT) to 67 and 68.2 HRC for treatments at −110 °C for 18 h and −196 °C for 38 h, respectively (following sequence *A*) [115].

In most papers dealing with the effects of CT on the toughness of high-speed steels (irrespective of the method used, e.g., CVN impact toughness, toughness measured on unnotched specimens, flexural strength, etc.), CT increased this property [50,62,64,68,114,186]. This change is due to the fact that the steels were tempered to their secondary hardness. High-temperature tempering induces martensite softening, making this phase more amenable to storing plastic deformation energy at the crack tip during crack propagation. In addition, more additional SGCs provide serious barriers to crack propagation, similar to ledeburitic steels subjected to CT. And finally, toughness improvement (with no hardness sacrifice at the same time) can be referred to as overall microstructural refinement, as Xu et al. have reported in their two recent works [186,315]. 

**Figure 44:**
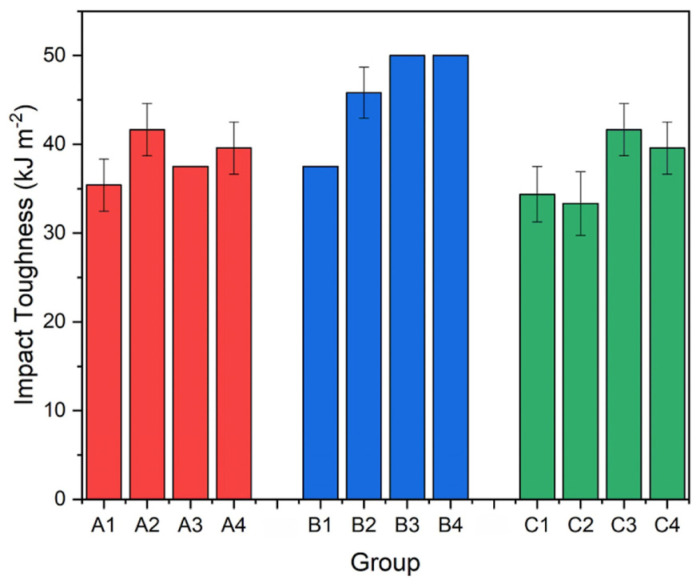
Charpy V-notch (CVN) impact toughness for three steel grades: A1–A4—AISI M2 steel; B1–B4—AISI M3:2 steel; C1–C4—AISI M35 steel. A1, A3, B1, B3, C1, and C3 were conventionally heat treated, and A2–A4, B2–B4, and C2–C4 were cryogenically treated at −196 °C for 24 h. The groups A1, A2; B1, B2; and C1, C2 were austenitised at 1230 (A), 1180 (B), and 1230 °C (C), respectively. The groups A3, A4; B3, B4; and C3, C4 were austenitised at 1180 (A), 1050 (B), and 1160 °C (C), respectively. Adapted from [67].

The effect of pre-treatment prior to CT, represented by the austenitisation temperature level, may also play a certain role in toughness variations. Unfortunately, the obtained results are quite contradictory. Fantinelli et al. [62], for instance, have reported that if the AISI M2 steel was austenitised at 1170 °C before cryogenic treatment at −190 °C for 24 h, then the toughness was improved, while austenitising at 1230 °C decreased this property. On the other hand, thorough investigations of the effects of austenitising temperatures and CT (−196 °C for 24 h) on the Charpy V-notch (CVN) impact toughness of three steel grades (AISI M2, AISI M3:2, and AISI M35) resulted in opposite results [67]. Better toughness (by 7–12%) was obtained for AISI M2 and AISI M3:2 steels after austenitising at higher temperatures, while the use of lower austenitising temperatures manifested almost no effect on these two grades. And finally, the toughness is always slightly reduced in the case of AISI M35 steel. The results obtained are summarised in Figure 44 [67]. Another parameter investigated in some research articles is the effect of pre-tempering prior to cryogenic treatment on toughness. It was found that this kind of treatment (sequence *E* or *F*) produces better toughness as compared with post-CT tempering (sequence *A*) for AISI M2 steel [62,66]. Very promising results in terms of toughness increase (measured by CVN or flexural strength methods) were also obtained by cyclic CT (−180 °C or −196 °C) combined with tempering (sequences *C* or *G*) for the treatment of AISI M2 or AISI T1 high-speed steels [64,66,114]. However, a slight toughness reduction due to CT (by sequences *A* or *F*) at −84 or −196 °C for 24 or 36 h has been reported for cobalt-containing wrought M35 steel [67,170].

**Figure 45:**
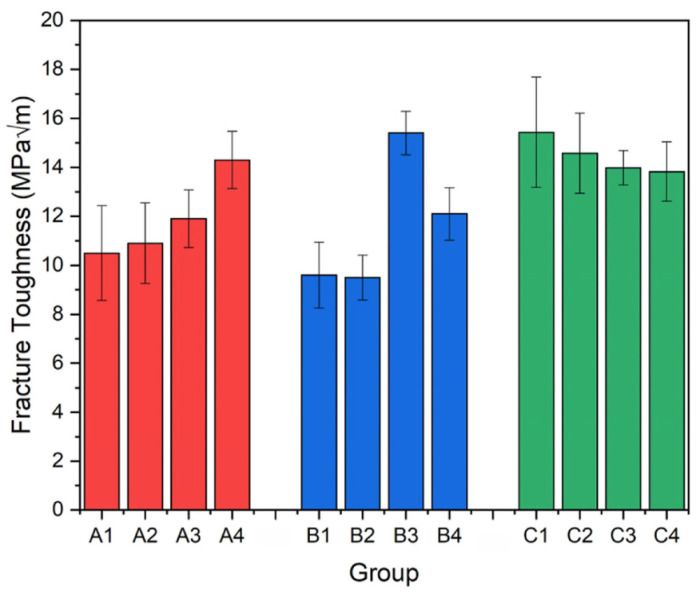
Fracture toughness for three steel grades: A1–A4—AISI M2 steel; B1–B4—AISI M3:2 steel; C1–C4—AISI M35 steel. A1, A3, B1, B3, C1, and C3 were conventionally heat treated, and A2–A4, B2–B4, and C2–C4 were cryogenically treated at −196 °C for 24 h. The groups A1, A2; B1, B2; and C1, C2 were austenitised at 1230, 1180, and 1230 °C, respectively. The groups A3, A4; B3, B4; and C3, C4 were austenitised at 1180, 1050, and 1160 °C, respectively. Adapted from [67].

Regarding the fracture toughness, a short treatment at −196 °C (1 h, sequence *A*) of AISI M2 steel had a detrimental effect on this property [63], but the K_IC_ was improved by 10% after a 24 h treatment at the same temperature (Figure 45) [67,168]. Conversely, other researchers observed only an improvement in K_IC_ in AISI M2 steel; this improvement was more pronounced when a higher austenitisation temperature was used [164]. For AISI M35 steel, the effect of austenitisation temperature prior to CT (sequence *A*) at −196 °C for 6 or 20 h on K_IC_ was investigated. Increases of 60%, 20%, and 15% were found for austenitising at 1070, 1100, and 1130 °C, respectively [164]. On the other hand, CT at −196 °C for 24 h deteriorated the fracture toughness of M3:2 and M35 steels; using a lower austenitisation temperature reduced K_IC_ more significantly than using a higher austenitisation temperature (Figure 45) [67].

Apparent fracture toughness (also known as K_a_) was studied for AISI M35 after cryogenic treatment at −180 °C for 24 h (sequence *A* or *E*) [350]. Only a marginal (1.4%) improvement in apparent fracture toughness was found for sequence *E*, while K_a_ increases of 3.5% and 4.6% were obtained for sequence *A*, with triple and double tempering, respectively. In another study [121], the effects of the same heat treatment schedules on the apparent fracture toughness of four different high-speed steels were examined–namely, M2 and M35 wrought steels and M3:2 and S6-5-3-8 PM steels. The wrought steels exhibited lower apparent fracture toughness than the PM steels at a certain hardness due to the non-uniform distribution of carbides arranged in strings. Cryogenic treatment caused an overall increase in toughness in Co-free grades (M2 or M3:2); a decrease was always observed for the two Co-containing grades.

When evaluating the wear resistance of high-speed steels, the specimens were subjected to high-temperature tempering (up to or around the secondary hardness temperature) unless otherwise designated. Among the investigated materials, the AISI M2 grade has been the most popular. 

**Figure 46:**
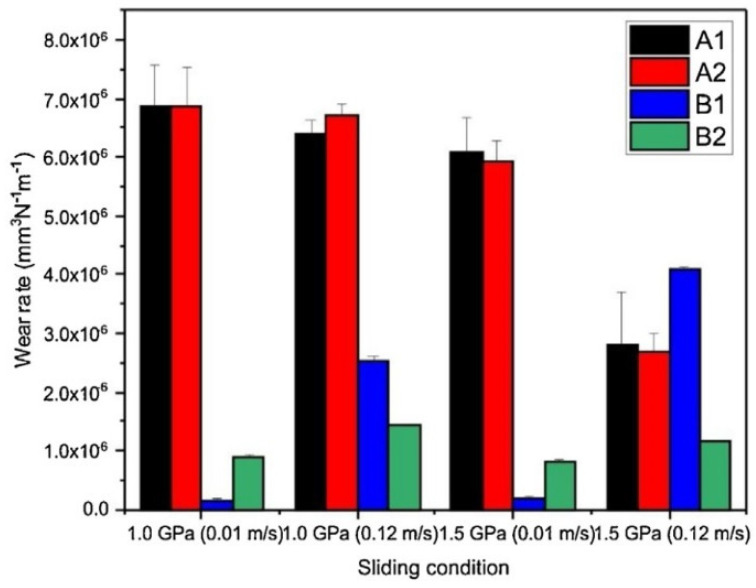
Effect of cryogenic treatment (at −196 °C for 24 h) and contact conditions (Hertzian pressures in GPa and sliding velocities in ms^−1^) on wear rate of AISI M2 (A1 and A2) and AISI M3:2 (B1 and B2) high-speed steels. The states A1, B1 represent conventional heat treatment (austenitising at 1230 °C, quenching, and tempering 3 times at 550 °C for 1 h for AISI M2; and austenitising at 1180 °C, quenching, and tempering 3 times at 540 °C for 2 h for AISI M3:2), and the states A2, B2 represent cryogenic treatments (following sequence *A*). Adapted from [168].

Leskovšek et al. [63] reported improved adhesive/abrasive wear resistance of AISI M2 steel after CT at −196 °C for 24 h and after tempering at 500, 550, or 600 °C, but tempering at 540 °C gave opposite results. This was related to the combined effect of hardness and fracture toughness, namely that the steel should have the highest possible fracture toughness (fulfilled after tempering at 550 and 600 °C) with sufficiently high hardness (64 HRC or more). Molinari et al. [50] and Li et al. [65] recommended CT at −196 °C for 35 or 24 h to achieve the best abrasive wear performance of AISI M2. Da Silva [114] and Jovičevič-Klug et al. [168] tested CHT and cryogenically treated (at −196 °C for 24 or 48 h) AISI M2 against alumina abrasives and found no effect of CT on abrasive wear resistance, as Figure 46 demonstrates. They noted that the reduction in retained austenite due to CT may not provide any benefit to wear performance under the given conditions, as retained austenite in CHT specimens may transform to martensite during testing. This could offset the improvement in wear performance due to more carbides in the cryogenically treated samples. Other works have used treatments at −180 or −190 °C (24 h) for CT, with moderately positive results [62,121,350]. The extent of the changes in wear performance can also be affected by the manufacturing route applied for steel preparation. Savas et al. [316] found only a small positive effect of cryogenic treatments at −196 °C on the wear resistance of cast AISI M2, while the same treatments on PM steel resulted in a significant reduction in wear rate. 

Low-temperature (150 °C) tempered AISI M2 steel subjected to cryogenic treatments showed significantly enhanced abrasive wear performance, up to 40% after CT at −110 °C for 18 h or up to 58% after CT at −196 °C for 38 h [115]. In another study, an optimisation experiment led to the final recommendation that 24 h treatment at −195 °C should be used to maximise the abrasive/adhesive wear performance of the given high-speed steel [11].

For other Co-free high-speed steels such as AISI W9 [10] and PM AISI M3:2 [121,168], cryo-temperatures of −180 to −196 °C were recommended to obtain better adhesive/abrasive wear performance. However, researchers also found that the effect of CT on wear performance is load-dependent; it is negative at low loads but exerts a strong positive influence at higher loads (>40%), as shown in Figure 46. Moreover, CT at −196 °C for 25 or 40 h (sequence *A*) had no effect on the abrasive wear resistance of S390 Microclean steel, but CT at −196 °C for 40 h after austenitising at 1130 °C (lower than the manufacturer’s recommended temperature) led to the maximum anti-galling performance of the given steel, as more undissolved carbides had been maintained in its microstructure [166,167]. Similarly, the anti-galling performance was also improved for AISI M3:2 steel [318].

For AISI M2, M3:2, and M35 high-speed steels, there was a moderate improvement in anti-galling performance after CT at −196 °C for 24 h (following sequence *A*) [121,318]. CT was not recommended for Co-containing high-speed steels because it reduced their wear performance [121]. However, this statement is not consistent with the results obtained by other investigators when testing Co-containing steel grades such as AISI T42 (at −185 °C for 8–24 h) or AISI M35. In these cases, a 38–50% adhesive/abrasive wear resistance improvement [10,170,312] and an anti-galling performance improvement (for AISI M35 after CT at −196 °C for 24 h) were reported.

Examination of the effects of different CT sequences has led to the conclusion that sequence *A* is most effective in the wear performance enhancement of most high-speed steels tested [11,62,115,121,318]. However, in selected trials, sequence *E*, with triple tempering at 570 °C before CT at −196 °C for 24 h, has also given promising results [170,267]. 

The effect of austenitisation temperature on the effectiveness of subsequent CT has also been evaluated. Pellizzari et al. [66] concluded that using an austenitisation temperature higher than that recommended by the steel manufacturer does not improve wear performance. Fantinelli et al. [62] found that for AISI M2 steel austenitised at either 1170 or 1230 °C before quenching and CT, sequence *E* gave the best wear performance, and sequence *A* resulted in the best wear performance when the samples were austenitised at 1200 °C. Voglar et al. [165] examined the corrosion resistance of three high-speed steel grades (AISI M2, AISI M3:2, and AISI M35) in different corrosive environments. They established that cast and wrought grades (AISI M2 and M35) did not respond to corrosive environments favourably after cryogenic treatment at −196 °C for 24 h, whereas the powder metallurgy produced AISI M3:2 high-speed steel with a very fine microstructure showed promising results.

Concluding remarks: The evaluation of the mechanical properties and wear behaviour of cryogenically treated high-speed steels has led to contradictory results. For example, an increase in hardness has been reported by some authors after CT (with the maximum at −196 °C for 24–40 h), but others have demonstrated no effect or a hardness decrease, even in analogous steels, due to CT. Improved toughness and fracture toughness were also observed in most cryogenically treated and high-temperature tempered steels, with the exception of the cobalt-containing grades. The sequence with tempering after CT (*A*) has a more effective influence on hardness than the sequences with pre-tempering (*E* or *F*). However, a rather opposite tendency was observed for the effect of these sequences on toughness. This is due to the fact that more austenite is subjected to CT in sequence *A,* in which it is transformed into martensite. This martensite contains more lattice defects and produces a greater number of nano-sized precipitates during subsequent tempering. Finally, the effect of additional small globular carbide formation should not be ignored. On the contrary, CT of steels before tempering can only affect a small part of the retained austenite (which remains in as-tempered steel microstructures). This must inevitably reduce the positive effect on hardness but have a negative effect on toughness. In some cases, the use of multiple CTs (sequences *C* or *G*) gave promising results in terms of mechanical properties. However, further research is needed to clarify this issue. So far, the effect of the austenitisation temperature on the mechanical properties of cryogenically treated high-speed steels is unclear. It can also be suggested that there is a ‘processing window’ (a combination of austenitising temperature, CT temperature, and dwell time at that temperature) where hardness and toughness can be improved simultaneously, albeit to a limited extent. A typical example is AISI M2 steel. In this steel, the use of austenitising at 1170 °C, followed by CT at −190 °C/24 h and tempering, resulted in an increase in the hardness of several HRCs and a simultaneous increase in CVN toughness.

The final wear performance of high-speed steels is affected by the content of the martensite, retained austenite, and carbides in their microstructure. Since the high-speed steels studied are mostly high-temperature tempered, they contain very little RA. Therefore, the effect of RA on their wear behaviour is minimal. The quenched and low-temperature tempered steels are an exception. In these cases, large improvements in abrasive/adhesive wear performance can be attributed to the reduction in RA due to CT. The number of carbides plays a crucial role in both abrasive/adhesive wear performance and anti-galling properties. It is noteworthy that the number of additional SGCs in most high-speed steels reached a maximum after CT at −196 °C (or close to this temperature) for 24 h following sequence *A* [61,67,115,318]. Bergmann et al. and Badisch and Mitterer [351,352] reported that it is desirable to maximise the number of hard carbides in steels if the highest possible wear performance is to be achieved. Therefore, treatment at −196 °C for 24 h resulted in the best wear performance of Co-free steels. In contrast, CT gave contradictory results for Co-containing HSS; therefore, this issue deserves further careful consideration. Finally, it appears that powder metallurgy produced HSS reacts much better to corrosion than forged grades after applying the same CT strategies.

### 4.6. Martensitic Stainless Steels

Martensitic stainless steels, shown in Table 6, are mostly alloys with a high content of Cr and other alloying elements. Therefore, they tend to retain high amounts of retained austenite in their microstructures when quenched from temperatures higher than the austenitising temperature recommended by the manufacturer. Some martensitic stainless steels exhibit a secondary hardness peak when tempered at around 500 °C. This is due to the complementary effect of the secondary RA-to-martensite transformation and carbide precipitation. However, to obtain high hardness in the tempered state, the amount of retained austenite should be carefully controlled before the steels are tempered. The application of cryogenic treatment is one way to regulate the amount of retained austenite in the steel before tempering.

For X30 CrMoN 15 1 steel, CT (at −198 °C for 24 h, sequence *A*) reduced the temperature of the secondary hardness peak by 70–100 °C after quenching in two different media—air or an aqueous solution of polyoxyethylene glycol [71]. However, the hardness of samples subjected to CT increased by 15–20 HRC compared to CHT when austenitised at 1100–1200 °C. A similar hardness increase was also found for cryogenically treated (−196 °C/24 h, sequence *A*) AISI 420 steel [210]. The use of a quenching medium had almost no effect on the final steel hardness, probably due to the excellent hardenability of the steel. The hardness of 0.15% C–14% Cr–13% Co–4.8% Mo–2.4% Ni steel austenitised at 1020 °C increased by 7 HRC by subjecting it to CT at −196 °C for 2 or 10 h (following sequence *A*) [69,70]. Very consistent results were obtained by cryogenic treatments of 0.17% C, 15% Cr, 11% Co, 3.3% Mo, 2.5% Ni, and 2% W steel at −196 °C for 20 h [321]. Cryogenic treatment at −196 °C for 10 h increased the hardness by 12 HRC after hardening from 1050 °C, indicating that the given CT effectively reduced the RA [69]. Furthermore, sequence *A* increased the hardness more effectively than post-tempering CT according to sequence *E* (a 3 HRC increase). Yildiz et al. [322] have tried to treat AISI 431 steel following sequence *F*. They have found only a very limited (of 17 HV) hardness increase due to cryogenic treatment at −180 °C for 6 h. The effect of cryogenic treatment temperature on the hardness of AISI 420 grade was investigated by Prieto et al. [68]. Cryogenic treatment at −196 °C for 2 h (following sequence *A*) increased the hardness from 560 HV10 (after CHT) to 585 HV10, but treatments at −40 or −80 °C did not affect hardness. For martensitic steel AISI 440C, CT at −80 °C for 5 h increased the final hardness by 2 HRC; cryogenic treatment at −196 °C resulted in a hardness increase of 4 HRC compared to the state after CHT [16]. The results on hardness variations indicate that temperatures above −80 °C are insufficient to transform most retained austenite to martensite. Thus, much lower temperatures, close to the boiling point of liquid nitrogen, are required to produce the maximum possible hardness increase. 

Yildiz et al. [322] have also examined the tensile properties of AISI 431 steel treated at −180 °C for 6 h, following sequence *F*. They have found that this kind of treatment leads to only a negligible ultimate tensile strength increase (20 MPa) but more pronounced yield strength increase (of 70 MPa). On the other hand, however, a 50% reduction in wear rate has been reported in the same work for the same steel treated in the same way. Yang et al. [323] have reported an almost 300 MPa yield strength increase for Ferrium S53 ultra-high strength stainless steel after CT following sequence *A*. 

Retained austenite reduction has a detrimental effect on the toughness of martensitic stainless steels. For example, the CVN impact toughness of AISI 420 steel after CT showed a general decrease in toughness, except for CT at −196 °C for 2 h, where there was a 4 J increase in CVN toughness [68]. For martensitic steel AISI 440C, CT at −196 °C for 24 h considerably reduced CVN impact toughness compared with CHT [16]. There were similar results on impact toughness for 13% Cr–12% Co–4% Mo–1.7% Ni–0.15% C martensitic stainless steel after CT at −75 °C (following sequence *A* or *G*) [17].

Potentiodynamic tests of cryogenically treated (−196 °C/24 h, sequence *A*) AISI 420 steel [210] showed almost no effect on corrosion resistance.

Concluding remarks: Overall, the results show that it is possible to increase the hardness of martensitic steels through cryogenic treatments. The maximum hardness increase can be obtained by CT at −196 °C by using sequence *A*, while higher temperatures have limited effects on this property. In most cases, however, the increase in hardness is accompanied by a significant loss in toughness. These changes are primarily due to a significant RA reduction. This reduction can be as much as 90% of the original amount of retained austenite obtained by conventional room-temperature quenching (see Section 3.6). The use of sequences *E* or *F* should generally be avoided in a heat treatment strategy since they have caused only negligible changes in hardness and tensile properties of martensitic stainless steels.

## 5. Conclusions, Recommendations, and Outlook

This review summarises the most typical steel classes for which cryogenic treatment has been investigated. A wide range of temperatures, from −40 to −269 °C, were used for the CT of steels. The duration of these treatments ranged from a few minutes to 1 week. Different heat treatment sequences were applied, such as CT before tempering, CT after tempering, CT between tempering cycles, cyclic CT followed by tempering, cyclic CT and tempering treatment, and others. Low- or high-temperature tempering treatments were also investigated for ledeburitic steels and high-speed steels. All of these treatments resulted in varying degrees of microstructural changes, which can be summarised as follows:

(i) Cryogenic treatment markedly reduces the retained austenite content. In this phase, there are high compressive stresses and more lattice defects. These factors also influence the thermal stability of retained austenite.
(ii) The martensite formed during cryogenic treatment is much finer than the athermally formed martensite. It also contains more lattice defects, such as dislocations or twins, and is non-homogeneous in its composition.
(iii) The above-mentioned changes in martensite increase its metastability; therefore, it tends to decompose under thermal exposure. This leads to accelerated precipitation of transient carbides, either when heated to ambient temperature after the low-temperature phase or when tempered at a low temperature. However, precipitation of stable carbides at high tempering temperatures is suppressed.
(iv) In ledeburitic tool steels, additional small globular carbides are formed by a quasi-diffusionless mechanism during the cryogenic period. The same may be valid for high-speed steels, ball bearing steels, and martensitic stainless steels, but further careful investigations are needed to make a conclusive statement. There is no evidence on presence of these particles in the case of carburised steels and hot work steels.

The heat treatment sequences involving CT after tempering should generally be avoided for ball bearing steels, carburised steels, ledeburitic cold work steels, high-speed steels, and martensitic stainless steels. These sequences reduce the influence of CT on the above microstructural changes compared to tempering after CT. This issue has not yet been clearly clarified for hot work steels, so further systematic studies on this topic are required. Attention should also be paid to the use of multiple CT cycles (combined or not combined with tempering). The same applies to the use of ageing (natural or artificial) before CT. 

Cryogenic treatment from −140 °C to −196 °C leads to the best microstructural improvements. The required CT duration varies greatly for the different classes of steel. It should be at least 8 h for carburised and ball bearing steels, about 20 h for hot work tool steels, 17–40 h for ledeburitic cold work tool steels, at least 24 h for high-speed steels, and at least 10 h for martensitic stainless steels.

The use of helium as a cryogenic medium cannot be recommended because the temperature of −269 °C is too low, and the phenomena leading to the desired microstructural changes take a long time.

The use of a higher than recommended austenitising temperature is generally discouraged for steels. However, in these cases, CT is an effective tool to reduce the amount of retained austenite to an acceptable level.

The following summary of mechanical and application properties has emerged from the review:

Cryogenic treatment significantly increases the prior-to-tempered hardness for all classes of steels discussed in this review. However, for proper use, the steels must be tempered. The application of CT prior to tempering (sequence *A*) is recommended for almost all classes of steels discussed in this review because it always results in a higher hardness than the other sequences. For ball bearing or ledeburitic steels, cyclic treatment (sequence *C*) has also given promising results. There is no conclusive recommendation for the heat treatment sequence of hot work tool steels because few studies have looked at sequences other than *F*; this issue remains open for future detailed investigation. A temperature of about −196 °C results in the greatest increase in hardness for all of the steel classes reported. To maximise hardness, the optimum treatment duration is close to 24 h for carburised steels, between 16 and 24 h for hot work steels, between 17 and 36 h for Cr-V ledeburitic steels, and between 36 and 48 h for D-class ledeburitic steels and bearing steels. Further systematic research is needed to determine the optimum time for high-speed steels and martensitic stainless steels. 

A common disadvantage of applying CT for ledeburitic, high-speed, and martensitic stainless steels is the loss of secondary hardness after high-temperature tempering. This cannot be completely avoided when using CT. 

To improve the tensile strength of carburised steels, sequence *E* with post-tempering CT is recommended, with a cryo-temperature < −180 °C. No knowledge is yet available on the effect of CT on the tensile strength of hot work steels. This issue should be the subject of comprehensive research.

The fatigue performance of carburised steels is slightly improved by using sequence *E* or *F,* but it is worsened by other treatment sequences. For this purpose, the use of low temperatures, around −196 °C, is recommended. For carburised gear parts, the use of CT always leads to a moderate deterioration in fatigue endurance in the bending of the tooth root. Tempering prior to CT (at −185 °C for 24 h) provides maximum fatigue performance for hot work tool steels like H11 or H13 grades. 

For hot work tool steels, pre-tempering prior to CT also appears to be useful for achieving maximum fatigue performance. However, the optimum CT temperature and duration remain unknown.

Increased hardness due to CT is accompanied by the reduced CVN toughness of carburised steels (in many cases) and ledeburitic tool steels (especially after low-temperature tempering). For martensitic stainless steel, the CVN impact toughness also deteriorates after high-temperature tempering. Heat treaters and end-users of the products should accept lower toughness of these steel classes after CT. The only exception is CT followed by high-temperature tempering for ledeburitic tool and high-speed steels, where toughness could be slightly improved. 

For ball bearing steels, CT may enhance the CVN impact toughness. However, due to the limited number of studies on this issue, it is premature to draw definitive conclusions. It is possible to improve the CVN toughness of H-class steels (except H21 grade) by applying cryogenic treatment. This improvement is greatest for CT at −154 or −184 °C, irrespective of the treatment duration. Regarding toughness improvement, tempering prior to CT (sequence *E* or *F*) or cyclic CT treatments (sequence *C*) have a more favourable effect than the ‘classical’ sequence (*A*). Note that the H-grade steels are high-temperature tempered. Moreover, hardness and toughness could be improved simultaneously for most H-class steels.

The flexural strength (also applied as a measure of toughness) of Cr-V ledeburitic steels could be improved slightly by CT if this treatment produces a sufficient number of additional small globular carbides, which act as barriers for crack propagation. Both the CVN impact toughness and flexural strength of Co-free high-speed steels could be improved by CT. Cyclic CT appears to be the best regime to increase toughness, applying a temperature of −196 °C for at least 24 h. The appropriate conditions for Co-containing steels are unclear and require further investigation. 

Although the available literature indicates an improvement in fracture toughness of H-class steels through CT, this topic needs further systematic research. For D-class cold work tool steel and Cr-V ledeburitic steels, a general deterioration in fracture toughness is inevitable; it can only be reduced by producing as many carbides as possible through CT. For Co-free grades of high-speed steels, fracture toughness could be improved through cryogenic treatment at −196 °C for 24–48 h, albeit to a limited extent. However, in this case, three tempering cycles, each at the secondary hardening temperature, must be performed after cryogenic treatment. The variations in fracture toughness of Co-containing grades remain unclear and require further research.

To maximise wear performance, the use of CT after quenching and before tempering is strongly recommended for most grades of steels. The cryo-temperature should be around −196 °C and the duration should be 24–36 h for carburised and ball bearing steels. Proper CT of H-class steels requires sequence *F*, with tempering, CT, and post-tempering. It is recommended to treat the steels at around −185 °C for 21–24 h to obtain the best wear performance. Due to the limited data available, additional investigations are recommended, particularly on the application of either the sequence with post-CT tempering or cyclic CT. 

Cryogenic treatment at the boiling temperature of liquid nitrogen for 24–48 h followed by low-temperature tempering produces the best wear performance of Cr-ledeburitic cold work tool steels. Tempering should be carried out at temperatures up to 210 °C. High-temperature tempering after CT could also provide better wear performance than CHT, especially for Cr-V steels, but this issue needs further investigation.

Although many studies have been carried out on the wear performance of cryogenically treated high-speed steels, the results are inconsistent. Indeed, a number of studies have recommended treatment at −196 °C for 24–40 h followed by tempering to maximise wear behaviour for Co-free steels (M2, M3:2, and others), while others have claimed that such treatment has no effect. Similar inconsistencies exist for Co-containing high-speed steels. Therefore, further research is needed in this particular area before a final recommendation can be formulated.

The use of CT for wrought ledeburitic and high-speed steels cannot be recommended if the tools are to be used in corrosive environments. On the other hand, CT could improve the corrosion resistance of steels produced by the powder metallurgy route. To maximise this property, treatments that produce a maximum number of additional small globular carbides are recommended. Nevertheless, the question of corrosion resistance of cryogenically treated steels requires further research to draw decisive conclusions.

Despite the great effort in examining cryogenically treated martensitic steels over the last three decades, some of impacts of this kind of treatment on resulting microstructures and mechanical properties are unclear to date. For the treatment of hot work tool steels, for instance, different sequences with pre-tempering prior to CT were used in most of the works. This pre-tempering treatment seriously distorts the obtained results. It concerns, for instance, the determination of the possible presence of additional small globular carbides in cryogenically treated steels. Therefore, it is highly recommended to conduct comprehensive research focussed on the impact of CT on resulting microstructures of hot work tool steels where sequence *A* should be used. For the treatment of high-speed steels, the tempering was mostly carried out at temperatures corresponding to the presence of the secondary hardness peak. There is not a relevant study available in the scientific databases that is devoted to the examination of resulting microstructures of any particular high-speed steel after CT and different tempering temperatures, from, for instance, 100 up to 600 °C. This makes a challenge for further research since such a study would enable us to determine the development of carbide counts, retained austenite amounts, and other characteristics as a function of tempering temperature for any particular cryogenic treatment regime.

The impact of cryogenic treatments on the corrosion resistance of several steel classes is also unclear to date. It only seems that powder metallurgy steels respond better to CT with respect to the corrosion performance than wrought steels. A more detailed and thorough investigation in this respect is desirable. As pointed out before, the impact of CT on the resulting microstructures of hot work tool steels is not completely clear yet. The same concerns mechanical properties such as strength, toughness, or wear performance. Further systematic research in this respect, mainly by using sequence *A* for the treatment, is recommended. The results obtained from the examination of cryogenically treated high-speed steels are often contradictory. This concerns almost all mechanical properties and wear and corrosion performance, irrespective of the examined steel grade(s). The main reason is, as above written, that tempering after CT was mostly carried out at temperatures corresponding to the presence of the secondary hardness peak. The above remark on the recommended further research focussed on the obtained microstructures concerns also the obtained mechanical properties and their development with tempering temperature for particular CTs and steel grades.

## Figures and Tables

**Table 1:** Overview of carburised steels and their cryogenic treatment covered by this review showing key investigations carried out: M—microstructure; A—retained austenite; H—hardness (t in the column includes tensile properties); R—residual stresses; N—notch/tooth root fracture resistance; K—fracture toughness; F—fatigue; W—wear resistance and tribology. The designation “x” means that the particular microstructural feature/mechanical property was investigated in the referenced paper.

Steel Grade/Designation	Main Element Content (wt.%)	Conditions of Cryogenic Treatment	M	A	H	R	N	K	F	W	Reference
En 353	0.17 C, 0.92 Mn, 1.09 Cr, 1.05 Ni, and 0.11 Mo	−196 °C/24 h	x		x					x	[18]
20MnCr5	0.19 C, 1.25 Mn, and 1.1 Cr	−70 °C/1 h; −196 °C/24 h; −269 °C/168 h	x		x					x	[33]
1.7131	0.15 C, 1.06 Mn, and 0.95 Cr	−196 °C/24 h	x							x	[46]
815M17	0.17 C, 0.92 Mn, 1.09 Cr, and 1.05 Ni	−80 °C/5 h or −196 °C/24 h			t					x	[47]
AISI 8620	0.17 C, 0.7–0.9 Mn, 0.4–0.6 Cr, and 0.4–0.7 Ni	−185 °C/16 h	x		x					x	[181]
SNCM 415	0.14 C, 0.53 Mn, 0.53 Cr, and 1.79 Ni	−85 °C/1, 12, or 24 h	x							x	[225]
18CrNiMo7-6	0.18 C, 0.7 Mn, 1.65 Cr, and 1.55 Ni	−30, −60, −80, or −196 °C; duration N/A		x	x	x	x				[226]
En353	0.15 C, 0.68 Mn, 0.76 Cr, and 1.19 Ni	−80 °C/5 h or −196 °C/24 h				x					[227]
AISI 8620H	0.2 C, 0.75 Mn, 0.52 Cr, 0.45 Ni, and 0.17 Mo	−100 °C/3.5 h	x							x	[228]
16MnCr5	0.18 C, 1.15 Mn, and 0.95 Cr	−100 °C/3.5 h	x							x	[228]
20Cr2Ni4A	0.21 C, 0.48 Mn, 1.45 Cr, and 3.55 Ni	−196 °C/1 h	x	x	x					x	[229]
17Cr2Ni2MoVNb	0.17 C, 0.77 Mn, 1.68 Cr, 1.6 Ni, 0.29 Mo, 0.04 Nb, and 0.1 V	−196 °C/1 h; −80 °C/1 h; −150 °C/1 h	x	x	x					x	[229,230]
AISI 8620	0.2 C, 0.7–0.9 Mn, 0.4–0.6 Cr, and 0.4–0.7 Ni	−40 °C/1 h	x							x	[231]
20CrNi2MoV	0.2 C, 0.61 Mn, 0.56 Cr, and 1.77 Ni	−80 °C/4 h or −196 °C/4 h	x							x	[180,232]
IS 2062	0.13 C and 0.88 Mn	−77 °C/3–24 h or −196 °C/3–24 h	x		x		x			x	[233]
21NiCrMo2	0.2 C, 0.77 Mn, 0.43 Ni, 0.55 Cr, and 0.18 Mo	−120 °C/2 h	x		x		x			x	[234]
18NiCrMo5	0.18 C, 0.6–0.9 Mn, 0.7–1 Cr, and 1.2 –1.5 Ni	−185 °C/1 or 24 h	x		t				x		[235,236]
SAE-4320	0.19 C, 0.55 Mn, 0.5 Cr, 1.8 Ni, and 0.25 Mo	−73 or −196 °C; duration N/A	x		x				x		[237]
SAE-9310	0.11 C, 0.55 Mn, 1.2 Cr, 3.25 Ni, and 0.1 Mo	−73 or −196 °C; duration N/A	x		x				x		[237]
16MnCr5	0.16 C, 1.1 Mn, and 1.12 Cr	−190 °C/24 h	x	x	x						[238]
16MnCr5	0.17 C, 1.14 Mn, and 1.1 Cr	−103 or −186 °C/3–24 h	x		x			x		x	[239]

**Table 3:** Overview of hot work tool steels and their cryogenic treatment covered by this review showing key investigations carried out: M—microstructure (p in the column—includes phase transformations); A—retained austenite; C—carbide precipitation; H—hardness (t in the column includes tensile properties); K—fracture toughness; F—fatigue; W—wear resistance and tribology; O—corrosion resistance. The designation “x” means that the particular microstructural feature/mechanical property was investigated in the referenced paper.

Steel Grade/Designation	Main Element Content (wt.%)	Conditions of Cryogenic Treatment	M	A	C	H	K	F	W	O	Reference
AISI H11	0.37 C, 5.32 Cr, 1.31 Mo, and 0.34 V	−154 or −184 °C/6, 21, or 36 h for both	x			x			x		[22]
AISI H11	0.37 C, 5.32 Cr, 1.31 Mo, and 0.44 V	−154 °C/6, 21, or 36 h	x			t	x				[23]
AISI H11	0.37 C, 5.32 Cr, 1.31 Mo, and 0.34 V	−154 or −184 °C/6–36 h for both	x			x			x		[122]
AISI H11	0.41 C, 4.86 Cr, 1.30 Mo, and 0.29 V	−80 or −196 °C/24 h for both	x						x		[153]
AISI H11	0.40 C, 5.05 Cr, 1.30 Mo, and 0.97 V	−154 or −184 °C/6, 21, or 36 h for both	x						x		[259,260]
AISI H11	0.43 C, 4.78 Cr, 1.40 Mo, and 0.47 V	−185 °C/8–32 h	x			x			x		[261]
AISI H13	0.4 C, 5.5 Cr, 1.4 Mo, and 1 V	−185 °C/8–32 h	x			x			x		[19]
AISI H13	0.40 C, 5.18 Cr, 1.44 Mo, and 0.92 V	−145 °C/24 h	x			t			x		[48]
AISI H13	0.39 C, 5.11 Cr, 1.28 Mo, and 0.99 V	−185 °C/32 h	x			x			x		[49]
AISI H13	N/A	−196 °C/35 h	x				x				[50]
AISI H13	0.4 C, 5.5 Cr, 1.4 Mo, and 1 V	−185 °C/8–32 h	x						x		[51]
AISI H13	0.39 C, 5 Cr, 1.27 Mo, and 0.93 V	−154 or −184 °C/6, 21, or 36 h for both	x			t	x		x		[52,262]
AISI H13	0.39 C, 5.42 Cr, 1.4 Mo, and 1 V	−185 °C/16 h	x					x			[52]
AISI H13	0.39 C, 5.2 Cr, 1.4 Mo, and 0.9 V	−196 °C/12 h				t	x				[118,256]
AISI H13	0.35 C, 5 Cr, 1.5 Mo, and 1 V	−196 °C/24 h	x	x		x			x		[125]
AISI H13	0.36 C, 4.82 Cr, 1.19 Mo, and 0.86 V	−72 or −196 °C/8 h both	x			t			x		[155]
AISI H13	0.40 C, 5.05 Cr, 1.30 Mo, and 0.98 V	−155 °C/6 h	x			x			x		[199]
AISI H13	0.38 C, 5.21 Cr, 1.12 Mo, and 0.9 V	−80 °C/24 h; −185 °C/24 h	x	x		x			x		[257]
AISI H13	0.38 C, 5.1 Cr, 1.4 Mo, and 1 V	−196 °C/24 h	x	x	x	x					[258]
AISI H13	0.39 C, 5.11 Cr, 1.28 Mo, and 0.99 V	−185 °C/32 h	x			x			x		[263]
AISI H13	0.44 C, 5.19 Cr, 1.42 Mo, 0.87 V, and 0.2 Cu	−72 °C/18 h or −196 °C/18 h	x			x			x		[264]
AISI H13	0.4 C, 5 Cr, 1 Mo, and 1 V	−185 °C/8–32 h	x						x		[265]
AISI H13	N/A	−196 °C/24 h	x	x	x	t					[266]
AISI H13	0.38 C, 5 Cr, 1.3 Mo, and 0.4 V	−180 °C/32 h	x						x		[267]
AISI H13	0.39 C, 5.4 Cr, 1.4 Mo, and 1 V	−185 °C/16 h	x							x	[268]
X37CrMoV5	0.38 C, 5 Cr, 1.2 Mo, and 0.4 V	−160 °C/12 or 30 h	p		x				x		[117]
X37CrMoV5	0.41 C, 5.01 Cr, 1.2 Mo, and 0.43 V	−160 °C/6, 12, or 24 h	x	x	x	x					[154]
AISI H21	0.3 C, 3.5 Cr, 0.41 V, and 9.75 W	−185 °C/6–30 h	x			x			x		[200,269]
AISI H21	0.3 C, 3.5 Cr, 0.41 V, and 9.75 W	−185 °C/24 h	x					x			[270]
CR7V	0.4 C, 6.1 Cr, 1.24 Mo, and 0.72 V	−196 °C/3, 6, or 12 h	x	x		x			x		[271]
AISI A8	0.55 C, 5 C, 1.4 Mo, and 1.25 W	Cyclic treatment; 5 cycles at −172 °C/−73 °C; a total of 15 h of treatment	x			x			x		[272]

**Table 5:** Overview of high-speed steels and their cryogenic treatment covered by this review showing key investigations carried out: M—microstructure; A—retained austenite; C—carbide precipitation; H—hardness; N—notch/tooth root fracture resistance; K—fracture toughness; W—wear resistance and tribology; O—corrosion resistance. The designation “x” means that the particular microstructural feature/mechanical property was investigated in the referenced paper.

Steel Grade/Designation	Main Element Content (wt.%)	Conditions of Cryogenic Treatment	M	A	C	H	N	K	W	O	Reference
AISI W9	0.81 C, 3.92 Cr, 3.1 Mo, 9.25 W, and 1.35 V	−80, −120, −160, or −196 °C/4 h each	x			x	x		x		[10]
AISI M2	0.86 C, 4.19 Cr, 4.55 Mo, 6.4 W, and 1.91 V	−196 °C/12, 24, or 36 h	x	x					x		[11]
AISI M2	0.86 C, 4.2 Cr, 6 W, 5 Mo, and 1.8 V	−196 °C/35 h	x			x	x	x	x		[50]
AISI M2	0.9 C, 4 Cr, 4.7 Mo, 6 W, and 1.7 V	−196 °C/24 h	x								[61]
AISI M3:2	1.29 C, 3.9 Cr, 4.8 Mo, 5.9 W, and 3 V	−196 °C/24 h	x								[61]
AISI M35	0.9 C, 4.1 Cr, 5.2 Mo, 6.2 W, 2 V, and 4.5 Co	−196 °C/24 h	x								[61]
AISI M2	0.87 C, 3.75 Cr, 7.65 W, 4.71 Mo, and 2.05 V	−190 °C/24 h	x	x		x	x		x		[62]
AISI M2	0.89 C, 3.91 Cr, 4.74 Mo, 1.74% V, and 6.1 W	−196 °C/1 h	x	x		x			x		[63]
AISI M2	0.85 C, 4.2 Cr, 5 Mo, 6 W, and 1.8 V	−196 °C/24 or 48 h, or −196 °C/3 times for 16 h	x		x	x	x				[64]
AISI T1	0.8 C, 4 Cr, 18 W, and 1 V	−196 °C/24 or 48 h, or −196 °C/3 times for 16 h	x		x	x	x				[64]
AISI M2	0.85 C, 4.1 Cr, 5 Mo, 6.15 W, and 1.95 V	−80, −120, −160, or −196 °C/1–24 h each	x			x			x		[65]
AISI M2	0.85 C, 4.2 Cr, 5 Mo, 6 W, and 1.8 V	−185 °C/6 or 20 h	x	x		x	x		x		[66]
AISI M35	0.9 C, 4.1 Cr, 6.2 W, 5.2 Mo, 2 V, and 4.5 Co	−196 °C/24 h	x			x	x	x	x		[67]
AISI M3:2	1.29 C, 3.9 Cr, 5.9 W, 4.8 Mo, and 3 V	−196 °C/24 h	x			x	x	x	x		[67]
AISI M2	0.9 C, 4 Cr, 6 W, 4.7 Mo, and 1.7 V	−196 °C/24 h	x			x	x	x	x		[67]
AISI M2	0.85 C, 4.1 Cr, 6.15 W, 5 Mo, and 1.95 V	−196 °C/4 h	x			x	x				[114]
AISI M2	0.88 C, 4.5 Cr, 6.55 W, 5.45 Mo, and 2.1 V	−110 °C/18 h or −196 °C/38 h	x	x		x			x		[115]
AISI M2	0.87 C, 4.3 Cr, 6.4 W, 5 Mo, and 1.9 V	−180 °C/24 h	x			x					[121]
AISI M35	0.93 C, 4.2 Cr, 6.4 W, 5 Mo, 1.8 V, and 4.8 Co	−180 °C/24 h	x			x					[121]
AISI M3:2	1.28 C, 4 Cr, 6.4 W, 5 Mo, and 3.1 V	−180 °C/24 h	x			x					[121]
HS6-5-3-8	1.3 C, 4.2 Cr, 6.3 W, 5 Mo, 3 V, and 8.4 Co	−180 °C/24 h	x			x		x	x		[121]
Exp. steel	0.85 C, 4.35 Cr, 2.8 Mo, 2.55 W, 2.1 V, and 4.5 Co	−196 °C/25 h	x	x		x		x	x		[164]
AISI M35	0.9 C, 4.1 Cr, 6.2 W, 5.2 Mo, 2 V, and 4.5 Co	−196 °C/24 h	x			x				x	[165]
AISI M3:2	1.29 C, 3.9 Cr, 5.9 W, 4.8 Mo, and 3 V	−196 °C/24 h	x			x				x	[165]
AISI M2	0.9 C, 4 Cr, 6 W, 4.7 Mo, and 1.7 V	−196 °C/24 h	x			x				x	[165]
S390 Microclean	1.47 C, 4.83 Cr, 10.05 W, 1.89% Mo, 4.77% V, and 8.25% Co	−196 °C/25 or 40 h	x			x		x	x		[166]
S390 Microclean	1.47 C, 4.83 Cr, 10.05 W, 1.89% Mo, 4.77% V, and 8.25% Co	−196 °C/25 or 40 h	x			x			x		[167]
AISI M3:2	1.29 C, 3.9 Cr, 5.9 W, 4.8 Mo, and 3 V	−196 °C/24 h	x			x		x	x		[168]
AISI M2	0.9 C, 4 Cr, 6 W, 4.7 Mo, and 1.7 V	−196 °C/24 h	x			x		x	x		[168]
AISI M2	0.92 C, 4 Cr, 6 W, 5 Mo, and 2 V	−196 °C/168 h	x								[169]
AISI M35	0.89 C, 4.17 Cr, 6.09 W, 4.66 Mo, 1.79 V, and 4.55 Co	−80 or −196 °C/24 or 36 h	x	x		x	x		x		[170]
AISI M2	0.83 C, 4.25 Cr, 6.08 W, 4.2 Mo, and 1.78 V	−180 °C/24 h	x		x						[185]
AISI M35	0.92 C, 3.82 Cr, 5.97 W, 5.13 Mo, 1.84 V, and 5 Co	−196 °C/3 min–48 h	x	x	x	x	x				[186]
AISI M2	0.9 C, 4 Cr, 6 W, 4.7 Mo, and 1.7 V	−196 °C/24 h	x								[208]
AISI M3:2	1.29 C, 3.9 Cr, 5.9 W, 4.8 Mo, and 3 V	−196 °C/24 h	x								[208]
AISI M35	0.9 C, 4.1 Cr, 6.2 W, 5.2 Mo, 2 V, and 4.5 Co	−196 °C/24 h	x								[208]
AISI M2	0.83 C, 4.1 Cr, 6.3 W, 5.1 Mo, and 1.92 V	−70 °C/duration N/A	x	x		x					[209]
AISI M2	0.82 C, 4.1 Cr, 6.1 W, 4.5 Mo, and 2.1 V	−155 °C/6 h	x						x		[310]
AISI M35	0.9 C, 4 Cr, 6 W, 5.2 Mo, 1.7 V, and 4.7 Co	−185 °C/16, 32, or 48 h	x			x					[311]
AISI T42	1.27 C, 4 Cr, 9.5 W, 3.6 Mo, 3.2 V, and 10 Co	−185 °C/8, 16, or 24 h	x						x		[312]
AISI T42	1.27 C, 4 Cr, 9.5 W, 3.6 Mo, 3.2 V, and 10 Co	−185 °C/8, 16, or 24 h	x								[313]
AISI M3:2	1.29 C, 3.9 Cr, 5.9 W, 4.8 Mo, and 3 V	−196 °C/24 h	x	x	x						[314]
AISI M2	0.85 C, 4.2 Cr, 5 Mo, 6 W, and 1.8 V	−180 °C/24 h	x			x		x	x		[315]
AISI M35	0.92 C, 3.82 Cr, 5.97 W, 5.13 Mo, 1.84 V, and 5 Co	−196 °C/5 h	x	x	x	x	x				[315]
AISI M2	Cast: 0.81 C, 4.87 Cr, 5.41 Mo, 6.12 W, and 2.15 VPM: 0.72 C, 4.15 Cr, 5.04 Mo, 6.59 W, and 1.89 V	−196 °C/16 or 24 h	x			x			x		[316]
AISI M2	0.87 C, 4 Cr, 6 W, 4.9 Mo, and 1.9 V	−196 °C/24 h	x			x					[317]
AISI M2	0.9 C, 4 Cr, 6 W, 4.7 Mo, and 1.7 V	−196 °C/24 h	x			x	x	x	x		[318]
AISI M3:2	1.29 C, 3.9 Cr, 5.9 W, 4.8 Mo, and 3 V	−196 °C/24 h	x			x	x	x	x		[318]
AISI M35	0.9 C, 4.1 Cr, 6.2 W, 5.2 Mo, 2 V, and 4.5 Co	−196 °C/24 h	x			x	x	x	x		[318]

**Table 6:** Overview of martensitic stainless steels and their cryogenic treatment covered by this review showing key investigations carried out: M—microstructure (p in the column—includes phase transformations); A—retained austenite; C—carbide precipitation; H—hardness; N—notch/tooth root fracture resistance; T—tensile properties; W—wear resistance and tribology. The designation “x” means that the particular microstructural feature/mechanical property was investigated in the referenced paper.

Steel Grade/Designation	Main Element Content (wt.%)	Conditions of Cryogenic Treatment	M	A	C	H	N	T	W	Reference
AISI 440C	0.93 C, 16.94 Cr, and 0.45 Mo	−80 °C/5 h or −196 °C/24 h	x			x	x			[16]
exp. steel	0.15 C, 14 Cr, 12.5 Co, 4 Mo, and 1.7 Ni	−75 °C/2 h	x	x		x				[17]
AISI 420	0.17 C and 12.83 Cr	−40, −80, or 196 °C/1 or 2 h each	x			x	x			[68]
exp. steel	0.15 C, 14 Cr, 13 Co, and 4.8 Mo	−196 °C/10 h	p			x				[69]
exp. Steel	0.15 C, 14 Cr, 12.5 Co, 4.5 Mo, and 2 Ni	−196 °C/2 h	x	x	x	x				[70]
X30 CrMoN 15 1	0.34 C, 16.2 Cr, 1.1 Mo, 0.04 V, and 0.33 N	−198 °C/24 h	x	x		x				[71]
AISI 440C	0.9 C, 18 Cr, and 1 Mo	−80, −120, −150, or −184 °C/6–36 h							x	[72]
AISI 420	0.17 C and 12.83 Cr	−196 °C/24 h	x	x	x	x				[210]
exp. steel	0.17 C, 15 Cr, 11 Co, 3.3 Mo, 2.5 Ni, and 2 W	−196 °C/20 h	x	x	x	x				[321]
AISI 431	0.188 C, 15.597 Cr, 1.53 Ni, 0.148 Mo, and 0.113 Cu	−180 °C/6 h	x			x		x		[322]
Ferrium 353	0.21 C, 10 Cr, 14 Co, 5.5 Ni, 1 W, and 0.3 V	−196 °C/12 h	x					x		[323]

## Data Availability

Data will be made available on request.

## Abbreviations

AISI	American Iron and Steel Institute
CHT	Conventional heat treatment
CT	Cryogenic treatment; cryo-treatment
SZT	Sub-zero treatment
EDX	Energy dispersive X-ray spectroscopy
PM	Powder metallurgy
HSS	High-speed steel
ECs	Eutectic carbides
FCC	Face-centred cubic
SCs	Secondary carbides
SGCs	Small globular carbides
RA	Retained austenite
CVN	Charpy V-notch (impact energy). Note: the values of CVN toughness were obtained by room-temperature testing unless otherwise indicated.
K_IC_	Fracture toughness (critical stress intensity factor)
K_a_	Apparent fracture toughness
HRC	Rockwell hardness
HV	Vickers hardness
